# Grey and white matter metrics demonstrate distinct and complementary prediction of differences in cognitive performance in children: Findings from ABCD (N= 11 876)

**DOI:** 10.1101/2023.03.06.529634

**Published:** 2023-03-08

**Authors:** Lea C. Michel, Ethan M. McCormick, Rogier A. Kievit

**Affiliations:** 1. Cognitive Neuroscience Department, Radboud University Medical Center, 6525 GA Nijmegen, The Netherlands; 2. Methodology and Statistics, Institute of Psychology, Leiden University, Leiden, Netherlands; 3. Department of Psychology and Neuroscience, University of North Carolina, Chapel Hill, United States

## Abstract

Individual differences in cognitive performance in childhood are a key predictor of significant life outcomes such as educational attainment and physical and mental health. Differences in cognitive ability are governed at least in part by variations in brain structure. However, studies commonly focus on either grey or white matter metrics, leaving open the key question as to whether grey or white matter microstructure play distinct roles supporting cognitive performance or if they are two ways to look at the same system complementary.

To compare the role of grey and white matter in supporting cognitive performance, we used regularized structural equation models to predict cognitive performance with grey and white matter measures. Specifically, we compared how morphometric grey matter measures (volume, cortical thickness and surface area) and indices of white matter microstructure (volume, fractional anisotropy and mean diffusivity) predicted individual differences in general cognitive performance. The models were tested in a large cohort of children (ABCD Study, N=11876) at 10 years old.

We found that grey and white matter metrics bring partly different information to predict cognitive performance. Indeed, model selection approaches consistently demonstrated both tissues were needed, compared to simpler models with only grey or only white that explained respectively 12.3% and 10.9% of the variance in cognitive performance, the combined models explained 15.4% of the variance. Zooming in we additionally found different metrics within grey and white matter had different predictive power, and different regions for grey and white matter had the strongest association with cognitive performance differences.

These results show that studies focusing on a single metric in either grey or white matter to study the link between brain structure and cognitive performance are missing a key part of the equation.

## Introduction

The field of cognitive neuroscience is premised on the hypothesis that differences in cognitive performance can be understood in part by studying differences in brain structure and function (Basten et al., 2015). Although much is known about the relation between grey and white matter structure and cognitive performance (Tamnes, Østby, Walhovd, et al., 2010; Tamnes, Østby, Fjell, et al., 2010; Magistro et al., 2015; Muetzel et al., 2015; Schnack et al., 2015; Deoni et al., 2016), less is known about how the structure of the two tissues *together* explain differences in cognitive performance.

One way to look at this challenge is to consider grey and white matter as two sides of the same coin. At the cellular level, they are both composed of neurons, although different parts (cell bodies, dendrites, synapses and axons for grey matter and (un)myelinated axons for white matter), glial cells and vasculature (Wandell, 2016; Purves et al., 2019). Moreover, recent findings observe even more overlap, namely that differences in one tissue (white matter myelination) may affect the other tissue (cortical thickness) (Natu et al., 2019), suggesting they may capture the same neurobiological properties using different techniques.

A counter argument would be that grey and white matter are two distinct brain properties with distinct mechanistic roles. Using a twin study design, Baaré and colleagues reported that grey and white matter volume shared 68% heritability, suggesting both overlapping and distinct genetic mechanisms (Baaré et al., 2001). Recent attention has focused on their different transcriptome patterns, highlighting the differentiation of their cells and their specific functional roles (Mills et al., 2013). Dendritic trees enable the neurons to receive a significant amount of information from distinct synapses at the same time (Hawkins & Ahmad, 2016), which will be integrated within the cell bodies and propagate to other regions through white matter tracts (Suminaite et al., 2019). Studies have shown the importance of both dendritic network and myelinated axons to provide faster and more efficient cognitive performance (Kail, 1997; Tamnes et al., 2012; Rolls & Deco, 2015), thus illustrating the potentially complementary roles of grey and white matter to explain differences in cognitive performance.

A key issue to address is the fact that neuroimaging techniques are, at best, indirect proxies of underlying biological differences. Technical limitations due to scanning, impact of software, resolution, atlas, partial volume effects and other challenges may complicate both the identification of the grey matter/white matter boundary, as well as the direct measurement of these two tissues (Murgasova et al., 2007; Cabezas et al., 2011; Tohka, 2014).

In summary, there is compelling evidence to suggest that grey and white matter play complementary roles in explaining differences in cognitive performance. To date, few studies investigated the combining effects of grey and white matter differences on cognitive performance and so far the literature point towards a complementary roles of the two tissues (Østby et al., 2011; Kievit et al., 2014; Ritchie et al., 2015).

The causes of this paucity are manifold. First, study designs tend to focus on either classical MRI sequences (T1,T2,T2*) or diffusion weighted imaging data. These are sequences with distinct scanner demands and require dedicated analysis expertise, which often leads to papers focusing on one of the two tissues. Moreover, the standard implementation in neuroimaging software, where a brain region/voxel is the *outcome* of a regression equation, makes it considerably more challenging to implement models where multiple brain metrics predict a single phenotypic outcome (e.g. cognitive performance).

To overcome these challenges, we investigated the different roles of grey and white matter metrics in predicting cognitive performance in childhood. By conducting a multimodal analysis with a latent variable in a large sample we overcome many existent weaknesses in previous work, and it allowed us to maximize the likelihood of distinguishing the competing hypotheses of interest. Doing so, this study provides new insights into the complementary information provided by grey and white matter in supporting cognitive performance.

## Methods

### Participants

The ABCD study (https://abcdstudy.org/) is an ongoing longitudinal study across 21 data acquisition sites enrolling 11 876 children from 9 years old to 16 years old. For more information on ABCD protocols and inclusion/exclusion criteria see Volkow and colleagues (Volkow et al., 2018).

This paper analysed the baseline sample (9–11 years old) from release 4.0 (https://abcdstudy.org/; http://doi.org/10.15154/1523041) that include a sample of 11,876 children. Data entry outliers for 10 participants in the Little Man task were replaced with NA and included in the full information maximum likelihood (FIML) estimation. We included participants with partial data across cognitive tasks and the neuroimaging data in our models.

**Figure 1:**
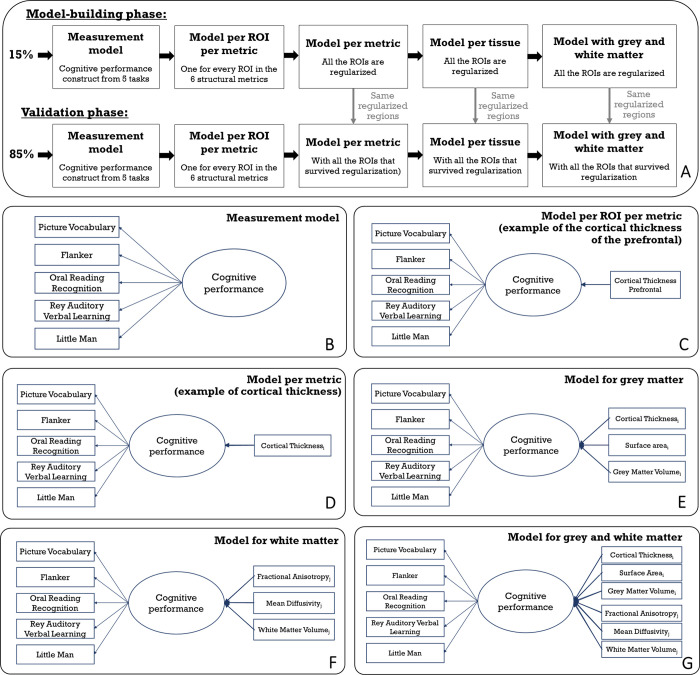
(A) Steps followed during the model-building and the validation phases of the study. The regions of interest fitted into the models of the validation phases resulted from an initial regularisation made in the model-building phase. (B) Measurement model of cognitive performance based on five cognitive tasks. (C) Example of a MIMIC model path diagram of a model per ROI per metric where the one metric of one region of interest (cortical thickness of the prefrontal) predict cognitive performance. (D) Example of a MIMIC model path diagram of a model per metric where all regions of interest of one metric (here cortical thickness) that survived regularization predict cognitive performance. (E) MIMIC model path diagram for the grey matter model with cortical thickness, surface area and grey matter volume predicting cognitive performance. i represents the regions of interest from each metric in grey matter that survived regularization in the model and can go from 1 to 34 (as we use the Desikan-Killiany atlas and average bilaterally). (F) MIMIC model path diagram for the white matter model with fractional anisotropy, mean diffusivity and white matter volume predicting cognitive performance. j represents the regions of interest from each metric in white matter that survived regularization in the model and can go from 1 to 20 (as we use the AtlasTrack atlas and average bilaterally). (G) Path diagram for the grey and white matter model with cortical thickness, surface area, grey matter, fractional anisotropy, mean diffusivity and white matter volume of the regions that survived regularization predicting cognitive performance.

Given the complexity of the analysis and the a priori challenges associated with high dimensional regularized structural equation modeling approaches, a fully preregistered analysis was not possible. However, to ensure robustness of our findings, we divided the sample into two subsets, allowing us to balance exploratory model optimisation and validation in a non-overlapping sample. In our study, a random sample of 15% of the data were used to optimize the model-building and estimation steps, and the other 85% of the sample was used as a validation sample (Srivastava, 2018) (Figure 1). The set of regions used in the validation sample was derived from the best predictive regions identified in regularized models estimated using the model-building sample (Schwarz, 1978) (the regularisation techniques will be explained more in the Experimental Design and Statistical Analysis section). This strategy allows us to jointly optimise robustness and flexibility in cases where model estimation, adaptation and convergence are non-trivial, whilst maintaining sufficient power in the validation set to be sufficiently well powered.

The model-building sample consisted of 1,781 children (49.1% female, mean age=9.9, SD=0.6, range=8.9–11.1) and the validation sample included 10,095 children (47.6% female, mean age=9.9, SD=0.6, range=8.9–11).

### Cognitive performance

In our modelling approaches, we want to focus on construct capturing a broad sampling of cognitive ability. To this end, we use the same procedure as identified in Sauce et al. (2022), by selecting five cognitive tasks from the NIH Toolbox Cognition Battery were selected: Picture Vocabulary, Flanker, Oral Reading Recognition, Rey Auditory Verbal Learning and Little Man (Sauce et al., 2022). They measure respectively language vocabulary knowledge, attention and inhibitory control, reading decoding skill, verbal learning and memory, visuospatial processing flexibility and attention. All of the tasks were administered using an iPad with support or scoring from a research assistant where needed. For more information on each task, see Luciana et al. (2018).

Next we specified a confirmatory factor model which posits a single latent factor for cognitive performance, reducing measurement error and increasing precision. It enables the model to compute the common variance between these five tasks and thus to have a more accurate representation of cognitive performance (Figure 1B). The measurement model has already been validated in another study, for more information on the rationale for the choice of cognitive tasks and the measurement model see Sauce and colleagues (Sauce et al., 2022). Note, the model chosen here should not be interpreted as a commitment to a strong ‘causal g’ (see Kievit et al., 2017), but rather as a way to specify a broad cognitive factor which will maximize our statistical power to capture the patterns of interest. A fruitful avenue of future work will be to examine the degree (or lack of) overlap in the neural mechanisms predicting each individual cognitive domain – a goal beyond the present paper.

### Brain structure measures

MRI and DTI scans were collected across the 21 research sites using Siemens Prisma, GE 750 and Philips 3T scanners. Scanning protocols were harmonized across sites. Full details of all the imaging acquisition protocols and the processing methods used in ABCD are outlined elsewhere (Casey et al., 2018; Hagler et al., 2019).

To determine the importance of grey and white matter we chose six structural metrics available in the ABCD study: cortical thickness (CT), surface area (SA) and volume (GMV) for grey matter and fractional anisotropy (FA), mean diffusivity (MD) and volume (WMV) for white matter.

#### Grey Matter

Grey matter measures were estimated from MRI scans with Free Surfer and include volume, cortical thickness and surface area (Casey et al., 2018). To allow regional analyses, the parcellation for the regions of interest (ROIs) has been made with the Desikan-Killiany atlas which generates 34 regions per hemisphere (Desikan et al., 2006). Considering the challenges of dimensionality in our SEM, we decided to average every regions of interest bilaterally.

#### White Matter

White matter structure can be measured through diffusion weighted imaging (DWI) and structural MRI (Wandell, 2016). DWI is a technique that allows researchers to record the diffusion of water in the brain using diffusion tensor imaging (DTI) to model the diffusion within the axons and myelin sheath, thus to analyse the directionality of the white matter tracts (Le Bihan, 2003; Assaf & Pasternak, 2008). Structural MRI provides a measure of volume while DTI computes fractional anisotropy, mean diffusivity, radial diffusivity and axial diffusivity (AD). White matter measures have been computed with Free-Surfer and include white matter volume, fractional anisotropy and mean diffusivity. The tracts were divided with the AtlasTrack atlas which created 37 regions of interest (17 regions per hemisphere and 3 regions aside) (Hagler et al., 2019). As for the grey matter metrics, we decided to average every regions of interest bilaterally.

### Experimental Design and Statistical Analysis

Next, we specified the core questions investigated by our analyses.

- Do the metrics of grey matter and white matter demonstrate complementary roles predicting cognitive performance?
- Do the different metrics in each tissue have unique predictive roles? If so, which metric is most important to predict cognitive performance?
- For each metric, do the different regions of interest have unique predictive roles?
- Do the same regions of interest have the strongest predictive power across different metrics in each tissue?

Our focus will be on both the tissue scale (grey and white matter) and the metrics scale (cortical thickness, surface area, grey matter volume, fractional anisotropy, mean diffusivity and white matter volume). For each scale, we will study if the predictors of cognitive performance each have a unique role (i.e. there is no overlap in the information contributed by the different variables to predict cognitive performance) or if they have complementary roles (i.e. there is a partial or total overlap in the information contributed by the different variables to predict cognitive performance).

The study uses structural equation model approach to evaluate the hypothesis that brain structural metrics predict the latent variable, cognitive performance. Structural equation models, and more particularly MIMIC models (Multiple Indicator Multiple Cause) offer an effective way to model cognition as a latent variable and to estimate the contribution of multiple simultaneous hypothesized causes to explain individual differences in cognitive performance (Jöreskog & Goldberger, 1975; Kievit et al., 2014).

First, we estimated a series of structural equation models to test how each metric in each individual region/tract predicted cognitive performance models (i.e., every region in all the six metrics have been computed independently). The key question of interest is the strength of the key parameter highlighted in bold (Figure 1C).

Next, to assess how each metric predicted cognitive performance we uses a regularized structural equation modelling (SEM) approach (Jacobucci et al., 2019) which incorporates a penalty on key parameters of interest. Specifically, it allows us to have many regions/metrics simultaneously predict the outcome, with a penalty on the path estimates from brain metrics to the cognitive latent variable, that induces sparsity. Regularization is a method that imposes a penalty in order to decrease the complexity of the model while keeping the variables that are the most important in predicting the outcome. For instance, it allows us to include the cortical thickness measures from all 34 regions of interest as simultaneously predicting cognitive performance with a lasso penalty which pushes parameter estimates of small or absent effects to 0, and retains only those regions which contribute meaningfully in predicting the outcome for the validation sample.

We developed a procedure to select a subset of regions/tracts based on their predictive ability in the model-building sample (e.g., 15% of the total sample). The output of each regularized model estimation was a set of all the regions/tracts with a regularized beta different from zero, or considered ‘important’ in a regularized framework. These regions were entered into the models predicting cognitive performance in the validation sample (e.g., 85% of the total sample). We implemented this process across six models per metric (Figure 1D), two models per tissue (Figures 1E–F) and the model combining grey and white matter metrics (Figure 1G). The benefit of this approach is to have a parsimonious representation of the key regions which help predict cognitive performance in each metric. We also examined ‘full’ models combining every region of interest within a metric (e.g. the 34 regions for cortical thickness), within a tissue (e.g. the 102 regions for the three grey matter metrics and the 60 regions for the three white matter metrics) and within grey and white matter metric (e.g. the 162 regions across the six metrics).

To compare the predictive information of grey and white matter, three models were fitted: one model with the regions extracted from the regularization of the three grey matter metrics, one model with the regions extracted from the regularization of the three white matter metrics and a final model with the regions extracted from the regularization of the grey and white matter metrics (e.g., as displayed in Figure 1A, the regions were selected because they survived the regularisation in the model-building sample). For each model, we use a likelihood ratio test to examine whether the inclusion of a tissue (grey/white matter) or a metric within a tissue (e.g. cortical thickness) improves the model compared to a model where the paths corresponding to an additional metric/tissue are constrained to 0. For each final model, we extracted the (adjusted) R-square to assess the proportion of variance in cognitive performance explained by each model.

**Figure 2:**
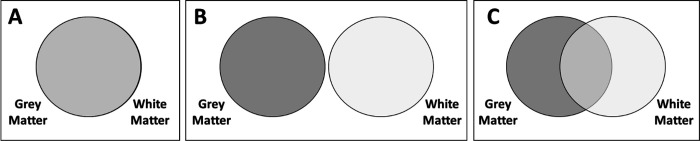
Hypotheses regarding the information provided by grey and white matter metrics. (A) Redundant, grey and white matter give the same information. (B) Independent, grey and white matter give completely different information. (C) Partially overlapping, grey and white matter give both similar and different information.

From the analyses, we can imagine our results being captured by one of three (simplified) scenarios:

- Grey and white matter metrics give the same, non-complementary information to predict cognitive performance. The model with only grey matter, only white matter or both will have a similar adjusted R-squared (Figure 2A), and model selection would favour a model with only one tissue.
- Grey and white matter metrics give fully distinct/complementary information to predict cognitive performance. Under this scenario, model selection would favour a model including both tissues and the joint R-squared would approximate the sum of the R-squared of each metric in isolation (Figure 2B).
- Grey and white matter metrics give complementary, but partially overlapping, information to predict cognitive performance. In this case, model selection would favour a model with both tissues, and the joint R-squared will be higher than the one of the most predictive tissue, but lower than the sum of the R-squared of each metric in isolation (i.e. the R-squareds are neither interchangeable nor fully additive) (Figure 2C).

We used the following guidelines to assess the good fit of the models: RMSEA<0.05 (acceptable: 0.05–0.08), CFI>0.97 (acceptable: 0.95–0.97) and SRMR<0.05 (acceptable: 0.05–0.10) (Mueller et al, 2008; Schermelleh-engel et al, 2003). All analyses were carried out for the data of the first wave using R, version 4.1.0 (http://www.r-project.org/) and the lavaan package (Rosseel, 2012). All models were fit using Maximum Likelihood Estimation, with FIML to account for missing data and robust estimation with adjusted standard errors to deal with deviations from (multivariate) normality.

### Data and Code Accessibility

Data can be requested through [https://nda.nih.gov/], and the code to reproduce our analyses is available on [https://osf.io/ryskf/].

## Results

### Measurement model (validation, parameters, invariance)

To assess cognitive performance, we used the same measurement model built by Sauce and colleagues (Sauce et al, 2021) and in a slightly different sample. Model estimates were highly similar.

The confirmatory factor model fitted the data well in the validation sample, x^²^ = 156.404, degrees of freedom (df)= 5, p<0.001, root mean square error of approximation (RMSEA) = 0.055 (0.00 – 0.070), comparative fit index (CFI)= 0.980, standardized root mean square residual (SRMR)= 0.020. This result demonstrates that the common variance among all five cognitive tasks can be captured by one latent variable that we call here “cognitive performance”.

**Figure 3:**
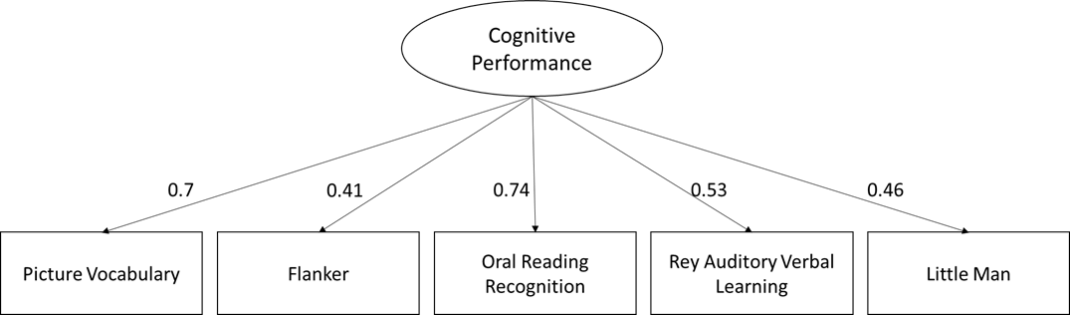
Path diagram of a measurement model of cognitive performance based on five cognitive tasks. All variables are standardized and significant.

Oral Reading Recognition task and Picture Vocabulary task have the strongest standardized factor loadings (0.74 and 0.7 respectively). Rey Auditory Verbal Learning task, Little Man task and Flanker task are mildly predicted by the construct between 0.41 and 0.53. (Figure 3). The results show little change when we add age as a predictor of the latent variable.

#### 1. Grey and white matter give both overlapping and unique information to predict cognitive performance

We fitted the full model including all regions/tracts that survived regularisation in grey & white matter metrics, and compared it to a model which includes the same predictors, but constrains either all grey or all white matter predictors to 0. This comparison allows us to test if the metrics in grey matter and white matter have complementary roles predicting cognitive performance.

The model with both grey and white matter fitted the data well (x^²^ = 798.945, df= 205, p<0.001, RMSEA = 0.017 (0.016 – 0.019), CFI= 0.933, TLI=0.915 SRMR= 0.011) and showed the best performance among the three models (AICdiff > 209, BICdiff > 69 in favour of the model with both grey and white matter), and explained 15,4% of the variance in cognitive performance. The model with only grey matter explained 12,3% of the variance and the one with only white matter explained 10,9% of the variance (Table 1). These findings demonstrate that grey and white matter bring both overlapping and unique information to predict the difference in cognitive performance in line with hypothesis C – ‘partially overlapping’ – in Figure 2.

#### 2. Some metrics are more predictive of cognitive performance than others

**Figure 4:**
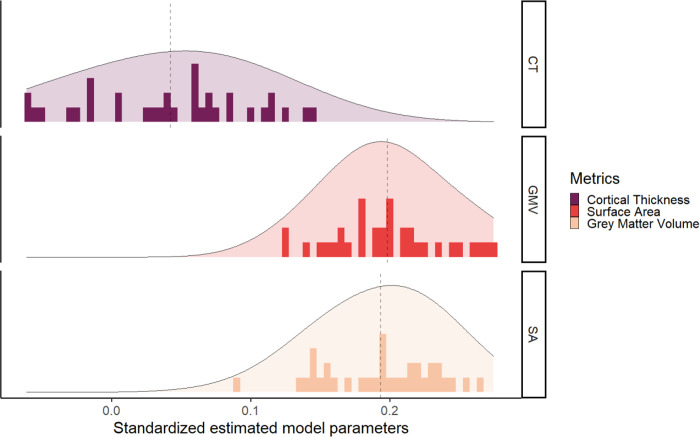
Graph of the standardized estimated model parameters on how one region of interest in one metric predicts cognitive performance in the models per ROI per metric for the three grey matter metrics.

Next, we investigated if the different metrics in each tissue have a unique predictive role, and thus if the choice of the metric was important when you want to predict cognitive performance. Comparing the predictive power of the different metrics, we plotted the standardized estimate model parameters of every regions across metrics. Figure 4 shows the range of observed values in the path estimates for each metrics, within the grey matter metrics surface area (range *β_std_* = [0.092;0.265]) and volume (range *β_std_* = [0.123;0.275]) were overall stronger predictors of cognitive performance compared with cortical thickness (range *β_std_* = [−0.06;0.145]). This is somewhat surprising given the prominence of cortical thickness as the metric of choice in (developmental) cognitive neuroimaging studies of individual differences. For instance, since 2016, the number of papers using cortical thickness is 5 times greater than the number of papers using grey matter volume (N=4808 for cortical thickness and N=823 for grey matter volume in a PubMed search as of 16/09/2022).

**Figure 5:**
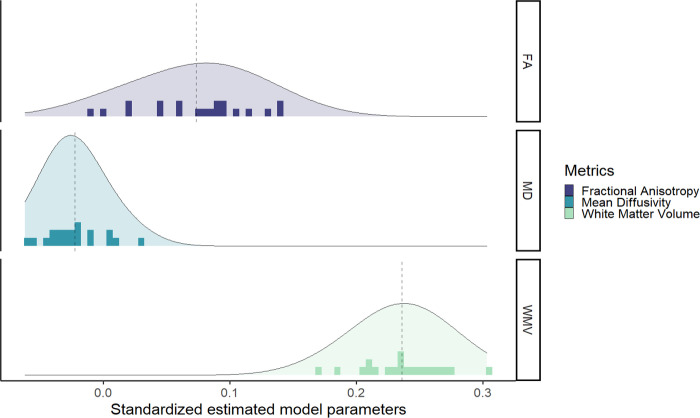
Graph of the standardized estimated model parameters on how one region of interest in one metric predicts cognitive performance in the models per ROI per metric for the three white matter metrics.

The results for the white matter metrics is represented in Figure 5. Surprisingly, white matter volume is overall the strongest predictor of cognitive performance (range *β_std_* = [0.169;0.275]), followed by fractional anisotropy (range *β_std_* = [−0.010;0.142]) and mean diffusivity (range *β_std_* = [−0.062;0.031]).

These results provide evidence that different metrics within grey and white matter better predict cognitive performance.

#### 3. Within a metric, not every region gives the same information to the prediction of cognitive performance

In addition to different metrics showing distinct predictive strengths as observed above, it is also highly plausible (e.g. Basten et al., 2015) that there is regional specificity – in other words, that different regions of interest might have a unique predictive role, and that this role depends on the metrics being studied. To assess the effects of the different regions within a metric, we compared a model with freely estimated parameters to a model where all the parameters are equality constrained, which captures the hypotheses that each region contributes equally. For every metrics in grey and white matter the free model showed the best fit to the data (AICdiff > 50, BICdiff > 2.5 in favour of the models with freely estimated parameters, both for the models with all the regions and the ones with the regularized regions).

In addition, the regularized models, which favour sparse models with only few predictors, always retained multiple regions even within the same metric. If a single ‘key’ region contains all relevant predictive information, we would not expect to observe this pattern. Across the nine models that underwent regularization, each model estimation showed at least 25% of all regions to have a regularized beta different from zero, demonstrating the regional specificity and complementarity hypothesized above. These converging findings demonstrate that to have a better picture of cognitive performance we would need to assess different regions in a metric simultaneously.

#### 4. Across metrics, different regions give unique information to the prediction of cognitive performance

Finally, we investigated if the same regions of interest bring unique information to predict cognitive performance across different metrics in each tissue (e.g. if the fractional anisotropy and white matter in a specific tract contain similar information then the regularization will likely regularize one of the metric to 0 for that given tract). A lasso regression was estimated with every region of interest in each grey or white matter metrics, the remaining regions were entered in a model as predictors of cognitive performance. This regression allows us to check which regions were nonessential to the prediction of cognitive performance when taking into account all the regions in the three metrics.

**Figure 6:**
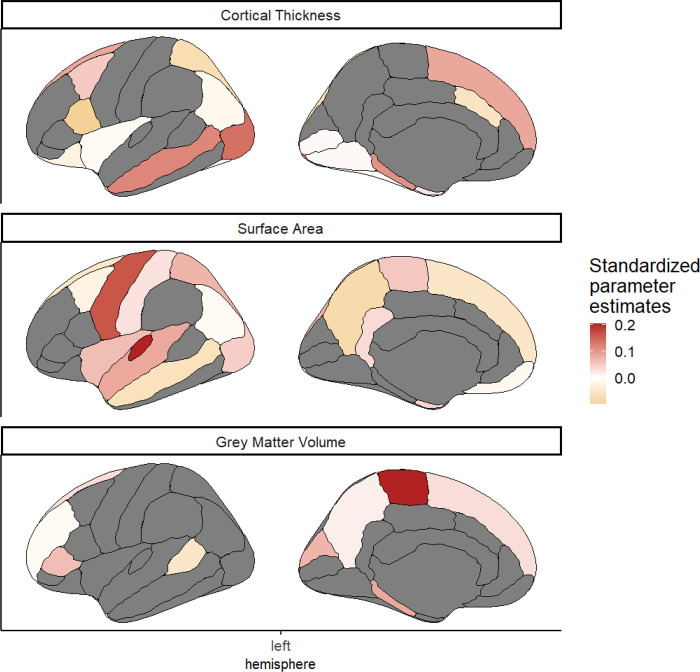
Graph of the regions of interest in each metric that survived the regularisation including the three metrics of grey matter. The colour indicated the standardized estimated model parameters

We found that despite the strong penalty include in the regularisations, the model still retained regions from each of the grey matter metrics, as well as each of the three white matter metrics, to be significantly predictive of cognitive performance (Figure 6). This suggests that different regions give unique information to the prediction of cognitive performance.

The complete results of all the models studies in this paper are available on OSF [https://osf.io/eygwz].

## Discussion

In a large developmental sample, we examined the role of grey and white matter in supporting cognitive performance. Using regularized structural equation model, we observed that the variance in cognitive performance explained by both grey and white matter (15.4%) is considerably greater than either one in isolation (12.3/10.9%), but not fully additive. This demonstrates that grey and white matter structure bring both unique as well as shared information in predicting individual differences in cognitive abilities in children.

In accordance with the present results, some studies have reported a partial overlap in the effect of grey and white matter measures on cognition (Kochunov et al., 2011; Hoagey et al., 2019) while others studies have described a more complete overlap (Tamnes, Østby, Fjell, et al., 2010). Zhao and colleagues proposed that these divergences in the literature are due to the different age of the samples and that the metrics are also capturing different mechanisms depending on the region (Zhao et al., 2021). Therefore, our results might vary depending on the age of the sample, the relative maturation of their brain and the regions of interest. This would be consistent with the findings from Østby et al. (2011), who detected a different importance of grey and white matter metrics across age for working memory performance and Fuhrmann et al. (2020) who observed age-dependent differences in the nature and strength of the relationship between white matter and cognitive performance (Østby et al., 2011; Fuhrmann et al., 2020).

Regarding the secondary goals of the study, we found evidence that different metrics within grey matter and white matter have distinct predictive power in this sample. For grey matter, surface area and volume show the best predictive power compared to cortical thickness, a surprising finding given the prevalence of studies which (only) use cortical thickness as a structural predictor of cognitive performance. However, this result is consistent with several studies reporting that surface area is more related to cognitive performance than cortical thickness (Borgeest et al., 2021; Pulli et al., 2023). Moreover, recent evidence within the same sample suggests that cortical thickness has lower reliability than surface area or volume, which may differentially attenuate the effects a given study observes (Parsons et al., 2023).

For white matter, we find (surprisingly) that in this sample white matter volume is the strongest predictor compared to more ‘advanced’ diffusion-based metrics such as fractional anisotropy and mean diffusivity. This is also unexpected since tract integrity measures are seen as more closely associated with the underlying physiology of tracts and their lesions (Fjell et al., 2008; Xing et al., 2021).

Hence, studies attempting to unveil the link between brain structure and cognitive performance by focusing solely on one tissue or one metric are at risk of overlooking the explanatory information conveyed only by the other tissue/metrics. These findings demonstrate the importance of the choice of the metrics when working with brain structure and future investigations regarding this question should use several metrics in both grey matter and white matter. However, this is not always a viable possibility (e.g. availability of the metrics, modelling approach). In that case, the choice of one metric over the other should be rationalized according to the other variables in the model, the regions of interest and the age of the sample – or the authors should make explicit that the findings are likely contingent on the metrics used in a study.

The study has several strengths. The uniquely large childhood sample of ABCD allows us to both explore the optimal model in an exploratory sample of only 15% of the data, and validate it in a test set. As such the sample size enables us to optimize exploration and confirmation, as well as increase parameter precision and power in line with recent recommendations (Marek et al., 2022). Moreover, by using a previously validated measurement model for the latent cognitive variable we further decrease measurement error and increase power and precision (Sauce et al., 2022).

However, the main strength of our study is what is commonly absent in the empirical literature: a multimodal analysis including several measures of grey and white matter and the comparison of their explanatory performance. We used three measures of grey matter structure (volume, cortical thickness and surface area) and three measures of white matter (volume, fractional anisotropy and mean diffusivity) and linked them to the latent measure of cognitive performance. Although studies using independent component analysis, partial least square regression and similar approaches have simultaneously integrated multimodal imaging data (Richard et al., 2020; Rasero et al., 2021), those more commonly examine the individual covariances between multimodal components or factors rather than examine whether distinct metrics provide unique complementary predictive information.

Despite these strengths, our study also has several limitations. First and foremost, the present analysis focuses on cross-sectional differences, studying the link between brain structure and cognitive performance at 10 years old. A longitudinal approach would allow us to better tease apart leading and lagging effects and provide provisional support in line with causal hypotheses (Wang & Cheng, 2020).

The ABCD sample also comes with its limitations. The study collected a representative sample of the USA, however this reflects a relatively over-studied subset of the world (often described as WERID, Garavan et al., 2018; Ghai, 2021). Given the impact of demographic characteristics on neuroimaging findings (Henrich et al., 2010; Falk et al., 2013; LeWinn et al., 2017), we cannot generalize our results outside of a WEIRD sample. The results are also specific to the studied age range (9–11 years old). This is a unique developmental period for the brain during which grey matter starts to decrease while white matter continues to increase (Bethlehem et al., 2022), so we expect the precise pattern of brain-behaviour relations to be contingent on this specific developmental period.

Moreover, our findings will be affected by certain pragmatic methodological choices. For example, bilaterally averaging the regions of interest across the two hemispheres allowed the possibility to do both regularization and maximum likelihood with a large number of regions. However, it precludes us observing any hemispheric specificity on the prediction of cognitive performance. The metrics used to reflect grey and white matter structure have been selected as commonly used neuroimaging measures, but the models might benefit from metrics that demonstrate a better representation of underlying cellular mechanisms.

Finally, the definition of grey and white matter as two different tissues measured by different metrics is inherently an oversimplification. MRI derived measures are proxies of the underlying brain structure and are not able (yet) to isolate specific biophysiological components of grey or white matter. For instance, cortical thickness is known to be sensitive to myelinisation of adjacent white matter (Natu et al., 2019). Therefore, overlap in statistical contributions of grey and white matter may be as much methodological artefact as biological reality.

To develop a full picture of the complementary roles of grey and white matter to the prediction of cognitive performance, future studies will need to examine large, multimodal, longitudinal and crucially diverse samples across the lifespan. Moreover, to produce a fuller picture of brain structure (and to explain a larger portion of the variance in cognitive performance), researchers will need to disentangle the neurobiological processes leading to changes in grey and white matter. In this regard, numerous new measures are developed to estimate cellular mechanisms more accurately. For instance, the analysis of sulcal depth and curvature would enable a detailed mapping of cortical structure in grey matter, while myelin water fraction (MWF) or magnetisation transfer ratio (MTR) offer more direct approaches to study axons microstructure and myelinisation (Timmers et al., 2016; Lipp et al., 2019). Beyond the metrics, the emergence of new scanners with higher resolution like the 11.7 Tesla or the Connectome scanners will unveil the structure within the six layers of the cortex and regional cortico-cortical connectivity (Nowogrodzki, 2018; Huang et al., 2021).

Although neuroimaging research is progressing rapidly, other techniques in complement to neuroimaging can shed a new light on brain structure and cognition, such as genetics (Grasby et al., 2020), in vivo resection (Heyer et al., 2022), computational synthesis of neurons (Kanari et al., 2022) or simulation neuroscience (Markram, 2012).

The present study was designed to evaluate the extent of the overlap between grey and white matter metrics in the prediction of cognitive performance. The main finding shows that grey and white matter metrics bring partially different information to predict cognitive performance. Within each tissue (i.e. grey or white matter), different metrics better predict cognitive performance and this also changes depending on the regions of interest. Taken together, these findings highlight the importance of the choice of the tissue, the metric and the area of the brain. Most importantly, the study provides evidence for both the unique and overlapping roles of grey and white matter to predict differences in cognitive performance in children. Therefore, future studies should consider both grey and white matter metrics or, if not possible, pay close attention to the choice of the metrics.

## Supplementary Material

Supplement 1

## Figures and Tables

**Table 1:** Estimates of the models fit and the variance explained by the predictors within each model.

	Grey Matter model	White Matter model	Grey and White Matter model
**R-square (adjusted)**	0.123	0.109	**0.154**
**AIC**	123164.3	123253.3	**122955.0**
**BIC**	123500.6	123489.4	**123420.1**

The best model is shown in bold.

## Significance Statement:

This paper enriches the recent debates on the challenges of linking variation in brain structure to phenotypic differences (Marek et al., 2022). We demonstrate that using latent variables (to improve power), structural equation modelling (to allow greater flexibility in linking brain to behaviour), and by simultaneously incorporating multiple measures of grey and white matter in a large sample, we demonstrate relatively strong and robust brain-behaviour associations, which highlight the complementarity of grey and white matter metrics in predicting cognitive performance as well as the importance of incorporating the full complexity of these associations over 1-to-1 linkages. This finding should lead researchers to consider integrating both grey and white matter measures when demonstrating a more comprehensive picture of brain-cognition relationships.
