# Supplementary material for: Grey and white matter metrics demonstrate distinct and complementary prediction of differences in cognitive performance in children: Findings from ABCD (N= 11 876)
Source: bioRxiv. 2023 Nov 6:2023.03.06.529634. Originally published 2023 Mar 8. Preprint. [Version 3] doi: 10.1101/2023.03.06.529634 (PMC10028815; doi:10.1101/2023.03.06.529634)

Supplementary materials

Grey and white matter metrics demonstrate distinct and complementary prediction of differences in cognitive performance in children: Findings from ABCD (N= 11 876)

Lea C. Michel[1], Ethan M. McCormick[1,2,3] & Rogier A. Kievit[1]

Contents

I. Summary of the variables
II. Correlations between the 5 cognitive tasks
III. Confirmatory Factor Analysis
IV. Individual models estimating cognitive factor from one region in one metric
   i. Cortical Thickness
   ii. Surface Area
   iii. Grey Matter Volume
   iv. Fractional Anisotropy
   v. Mean Diffusivity
   vi. White Matter Volume
V. Models per metric estimating cognitive factor from several regions in one metric
   i. Cortical Thickness (all regions and regularized regions)
   ii. Surface Area (all regions and regularized regions)
   iii. Grey Matter Volume (all regions and regularized regions)
   iv. Fractional Anisotropy (all regions and regularized regions)
   v. Mean Diffusivity (all regions and regularized regions)
   vi. White Matter Volume (all regions and regularized regions)
VI. Models per tissue estimating cognitive factor from several regions in three metrics
   i. Grey Matter Metrics (all regions and regularized regions)
   ii. White Matter Metrics (all regions and regularized regions)
VII. Models with grey and white matter metrics estimating cognitive factor from several regions in the six metrics
VIII. Comparison between models with grey & white matter metrics and with grey & white matter metrics & TIV

Summary of the variables

Supplementary Table 1. Characteristics of the variables used in the models. All the measures of grey and white matter share the same frequency and number of missing data per tissue.

Variable	Stats / Values	Freqs (% of Valid)	Graph	Missing
Subjectkey [character]		11 876 distinct values		0
interview_age [numeric]	Mean (sd) : 119 (7.5)<br>min ≤ med ≤ max:<br>107 ≤ 119 ≤ 133<br>IQR (CV) : 14 (0.1)	27 distinct values	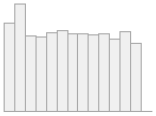	0 (0.0%)
sex [factor]	1. F<br>2. M	5680 (47.8%)<br>6196 (52.2%)	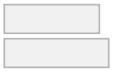	0 (0.0%)
Picture_Vocabulary [numeric]	Mean (sd) : 84.5 (8.1)<br>min ≤ med ≤ max:<br>29 ≤ 84 ≤ 119<br>IQR (CV) : 11 (0.1)	70 distinct values	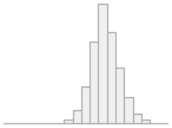	147 (1.2%)
Flanker [numeric]	Mean (sd) : 94 (9.1)<br>min ≤ med ≤ max:<br>51 ≤ 95 ≤ 116<br>IQR (CV) : 11 (0.1)	68 distinct values	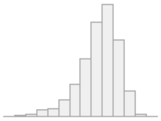	153 (1.3%)
Oral_Reading_Recognition [numeric]	Mean (sd) : 90.9 (6.9)<br>min ≤ med ≤ max:<br>59 ≤ 91 ≤ 119<br>IQR (CV) : 8 (0.1)	63 distinct values	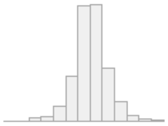	161 (1.4%)
Rey_Auditory_Verbal_Learning [numeric]	Mean (sd) : 44.2 (10)<br>min ≤ med ≤ max:<br>0 ≤ 45 ≤ 72<br>IQR (CV) : 13 (0.2)	69 distinct values	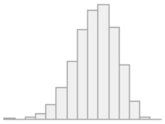	292 (2.5%)
Little_Man [numeric]	Mean (sd) : 58.9 (17)<br>min ≤ med ≤ max:<br>0 ≤ 56.2 ≤ 100<br>IQR (CV) : 25 (0.3)	32 distinct values	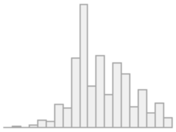	337 (2.8%)
bankssts_ct [numeric]	Mean (sd) : 0.1 (0.8)<br>min ≤ med ≤ max:<br>-6.8 ≤ 0.1 ≤ 4.3<br>IQR (CV) : 1.1 (7.9)	11521 distinct values	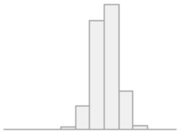	116 (1.0%)
forecepsmajor_fa [numeric]	Mean (sd) : -0.1 (1)<br>min ≤ med ≤ max:<br>-9.3 ≤ 0 ≤ 9.4<br>IQR (CV) : 1.2 (-8.2)	11107 distinct values	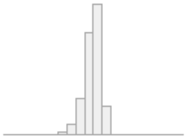	739 (6.2%)

Correlations between the 5 cognitive tasks

Supplementary Figure 1. Correlation plot of the five measures of cognition: Picture Vocabulary, Flanker, Oral Reading Recognition, Rey Auditory Verbal Learning and Little Man in the whole sample.

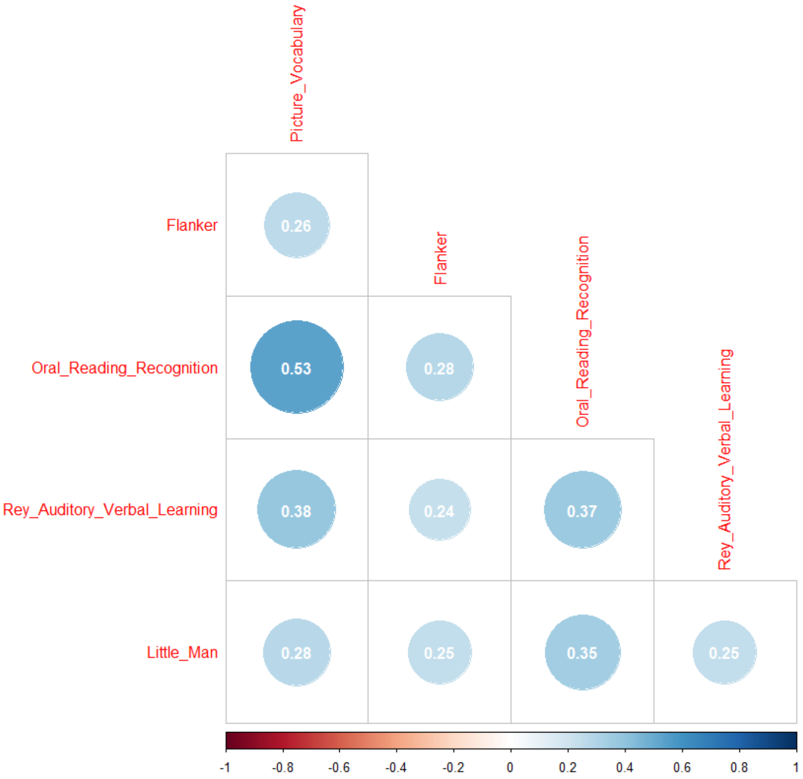

Confirmatory Factor Analysis

Supplementary Table 2. Latent variable loadings for the confirmatory factor analysis estimating the cognitive factor

Path	Estimate	SE	*p*	Standardized Estimate
Cognitive_factor =~ Picture_Vocabulary	1.000	0.000	NA	0.699
Cognitive_factor =~ Flanker	0.582	0.020	*p*<0.001	0.406
Cognitive_factor =~ Oral_Reading_Recognition	1.056	0.022	*p*<0.001	0.738
Cognitive_factor =~ Rey_Auditory_Verbal_Learning	0.753	0.018	*p*<0.001	0.527
Cognitive_factor =~ Little_Man	0.651	0.019	*p*<0.001	0.455

Individual models estimating cognitive factor from one region in one metric

Note: Every regions is bilaterally averaged. The results reported here are based on the validation sample (85%).

Cortical Thickness

Supplementary Table 3. Regression estimates for the model estimating how cortical thickness of one region predict the cognitive factor

**Path**			**Estimate**	**SE**	***p***	**Standardized Estimate**
Cognitive_factor	~	bankssts_ct	0.052	0.010	*p<0.001*	0.062
Cognitive_factor	~	caudalanteriorcingulate_ct	-0.049	0.010	*p<0.001*	-0.059
Cognitive_factor	~	caudalmiddlefrontal_ct	0.030	0.009	*0.001*	0.038
Cognitive_factor	~	cuneus_ct	0.088	0.009	*p<0.001*	0.114
Cognitive_factor	~	entorhinal_ct	0.049	0.009	*p<0.001*	0.061
Cognitive_factor	~	fusiform_ct	0.077	0.009	*p<0.001*	0.100
Cognitive_factor	~	inferiorparietal_ct	0.032	0.009	*p<0.001*	0.042
Cognitive_factor	~	inferiortemporal_ct	0.065	0.009	*p<0.001*	0.085
Cognitive_factor	~	isthmuscingulate_ct	0.003	0.010	0.778	0.003
Cognitive_factor	~	lateraloccipital_ct	0.103	0.009	*p<0.001*	0.139
Cognitive_factor	~	lateralorbitofrontal_ct	0.020	0.009	*0.027*	0.026
Cognitive_factor	~	lingual_ct	0.109	0.009	*p<0.001*	0.145
Cognitive_factor	~	medialorbitofrontal_ct	-0.042	0.009	*p<0.001*	-0.053
Cognitive_factor	~	middletemporal_ct	0.058	0.009	*p<0.001*	0.076
Cognitive_factor	~	parahippocampal_ct	0.098	0.009	*p<0.001*	0.127
Cognitive_factor	~	paracentral_ct	0.055	0.009	*p<0.001*	0.072
Cognitive_factor	~	parsopercularis_ct	-0.026	0.009	*0.006*	-0.032
Cognitive_factor	~	parsorbitalis_ct	-0.012	0.010	0.238	-0.014
Cognitive_factor	~	parstriangularis_ct	-0.021	0.009	*0.029*	-0.026
Cognitive_factor	~	pericalcarine_ct	0.086	0.009	*p<0.001*	0.113
Cognitive_factor	~	postcentral_ct	0.066	0.009	*p<0.001*	0.087
Cognitive_factor	~	posteriorcingulate_ct	-0.011	0.010	0.268	-0.014
Cognitive_factor	~	precentral_ct	0.081	0.009	*p<0.001*	0.108
Cognitive_factor	~	precuneus_ct	0.027	0.009	*0.002*	0.036
Cognitive_factor	~	rostralanteriorcingulate_ct	-0.044	0.010	*p<0.001*	-0.052
Cognitive_factor	~	rostralmiddlefrontal_ct	0.004	0.009	0.661	0.005
Cognitive_factor	~	superiorfrontal_ct	-0.011	0.009	0.220	-0.014
Cognitive_factor	~	superiorparietal_ct	0.032	0.009	*p<0.001*	0.044
Cognitive_factor	~	superiortemporal_ct	0.044	0.009	*p<0.001*	0.059
Cognitive_factor	~	supramarginal_ct	0.045	0.009	*p<0.001*	0.059
Cognitive_factor	~	frontalpole_ct	-0.051	0.010	*p<0.001*	-0.061
Cognitive_factor	~	temporalpole_ct	0.057	0.009	*p<0.001*	0.071
Cognitive_factor	~	transversetemporal_ct	0.053	0.009	*p<0.001*	0.066
Cognitive_factor	~	insula_ct	0.026	0.009	*0.006*	0.032

Supplementary Figure 2. Standardized parameter estimates of how the cortical thickness of each region of interest predict the cognitive factor

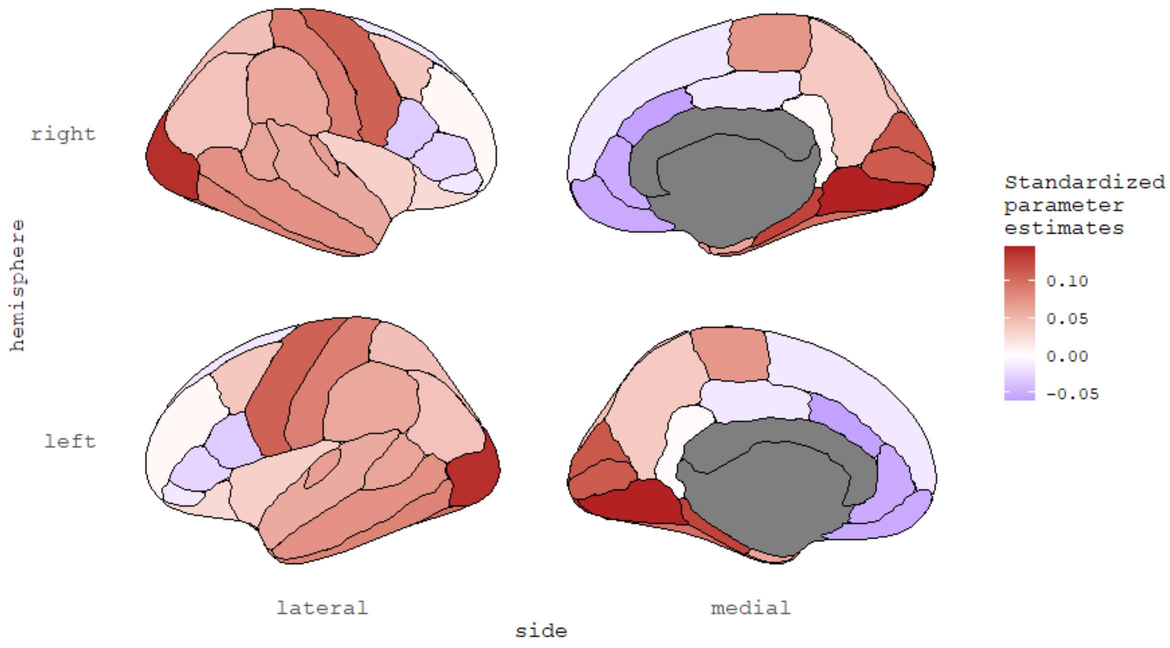

Surface Area

Supplementary Table 4. Regression estimates for the model estimating how surface area of one region predict the cognitive factor

Path			Estimate	SE	*p*	Standardized Estimate
Cognitive_factor	~	bankssts_sa	0.123	0.010	*p<0.001*	0.152
Cognitive_factor	~	caudalanteriorcingulate_sa	0.172	0.011	*p<0.001*	0.196
Cognitive_factor	~	caudalmiddlefrontal_sa	0.170	0.010	*p<0.001*	0.218
Cognitive_factor	~	cuneus_sa	0.099	0.009	*p<0.001*	0.133
Cognitive_factor	~	entorhinal_sa	0.134	0.009	*p<0.001*	0.170
Cognitive_factor	~	fusiform_sa	0.193	0.009	*p<0.001*	0.255
Cognitive_factor	~	inferiorparietal_sa	0.141	0.009	*p<0.001*	0.185
Cognitive_factor	~	inferiortemporal_sa	0.201	0.009	*p<0.001*	0.265
Cognitive_factor	~	isthmuscingulate_sa	0.118	0.011	*p<0.001*	0.153
Cognitive_factor	~	lateraloccipital_sa	0.147	0.009	*p<0.001*	0.196
Cognitive_factor	~	lateralorbitofrontal_sa	0.181	0.009	*p<0.001*	0.244
Cognitive_factor	~	lingual_sa	0.107	0.009	*p<0.001*	0.145
Cognitive_factor	~	medialorbitofrontal_sa	0.176	0.009	*p<0.001*	0.230
Cognitive_factor	~	middletemporal_sa	0.174	0.009	*p<0.001*	0.236
Cognitive_factor	~	parahippocampal_sa	0.123	0.009	*p<0.001*	0.157
Cognitive_factor	~	paracentral_sa	0.127	0.009	*p<0.001*	0.162

Cognitive_factor	~	parsopercularis_sa	0.154	0.010	*p<0.001*	0.193
Cognitive_factor	~	parsorbitalis_sa	0.168	0.010	*p<0.001*	0.219
Cognitive_factor	~	parstriangularis_sa	0.116	0.009	*p<0.001*	0.147
Cognitive_factor	~	pericalcarine_sa	0.067	0.009	*p<0.001*	0.092
Cognitive_factor	~	postcentral_sa	0.150	0.009	*p<0.001*	0.199
Cognitive_factor	~	posteriorcingulate_sa	0.157	0.011	*p<0.001*	0.195
Cognitive_factor	~	precentral_sa	0.173	0.009	*p<0.001*	0.230
Cognitive_factor	~	precuneus_sa	0.159	0.009	*p<0.001*	0.216
Cognitive_factor	~	rostralanteriorcingulate_sa	0.179	0.010	*p<0.001*	0.224
Cognitive_factor	~	rostralmiddlefrontal_sa	0.161	0.009	*p<0.001*	0.215
Cognitive_factor	~	superiorfrontal_sa	0.177	0.009	*p<0.001*	0.239
Cognitive_factor	~	superiorparietal_sa	0.142	0.009	*p<0.001*	0.190
Cognitive_factor	~	superiortemporal_sa	0.154	0.009	*p<0.001*	0.207
Cognitive_factor	~	supramarginal_sa	0.141	0.009	*p<0.001*	0.181
Cognitive_factor	~	frontalpole_sa	0.186	0.010	*p<0.001*	0.233
Cognitive_factor	~	temporalpole_sa	0.115	0.009	*p<0.001*	0.147
Cognitive_factor	~	transversetemporal_sa	0.111	0.010	*p<0.001*	0.142
Cognitive_factor	~	insula_sa	0.158	0.009	*p<0.001*	0.211

Supplementary Figure 3. Standardized parameter estimates of how the surface area of each region of interest predict the cognitive factor

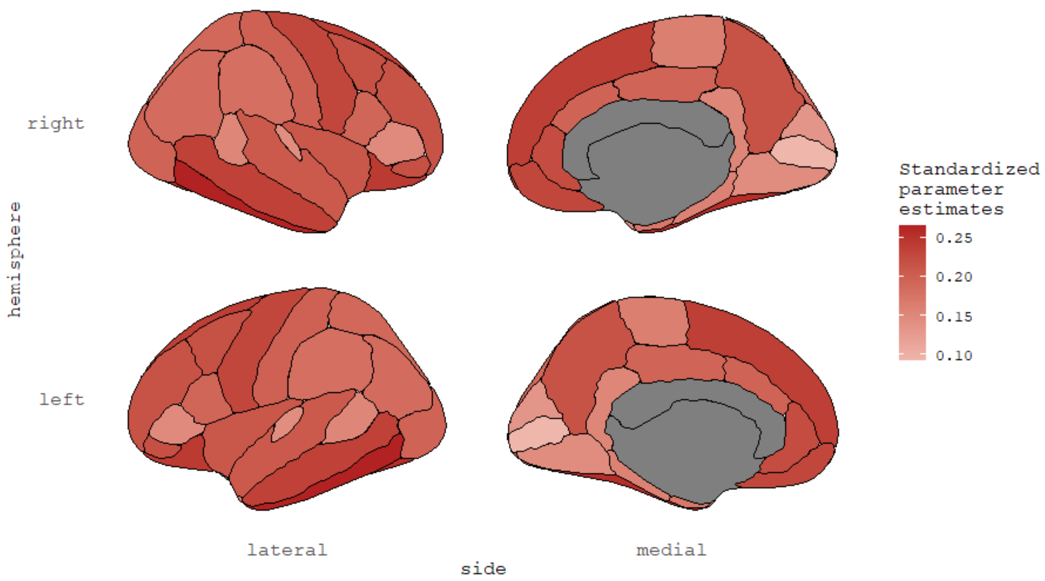

Grey Matter Volume

Supplementary Table 5. Regression estimates for the model estimating how grey matter volume of one region predict the cognitive factor

Path			Estimate	SE	*p*	Standardized Estimate
Cognitive_factor	~	bankssts_gmv	0.136	0.010	*p<0.001*	0.165
Cognitive_factor	~	caudalanteriorcingulate_gmv	0.158	0.011	*p<0.001*	0.171
Cognitive_factor	~	caudalmiddlefrontal_gmv	0.165	0.009	*p<0.001*	0.209
Cognitive_factor	~	cuneus_gmv	0.118	0.009	*p<0.001*	0.157
Cognitive_factor	~	entorhinal_gmv	0.152	0.010	*p<0.001*	0.191
Cognitive_factor	~	fusiform_gmv	0.200	0.009	*p<0.001*	0.261
Cognitive_factor	~	inferiorparietal_gmv	0.151	0.009	*p<0.001*	0.196
Cognitive_factor	~	inferiortemporal_gmv	0.203	0.009	*p<0.001*	0.265
Cognitive_factor	~	isthmuscingulate_gmv	0.117	0.010	*p<0.001*	0.150
Cognitive_factor	~	lateraloccipital_gmv	0.184	0.009	*p<0.001*	0.245
Cognitive_factor	~	lateralorbitofrontal_gmv	0.185	0.009	*p<0.001*	0.251
Cognitive_factor	~	lingual_gmv	0.133	0.009	*p<0.001*	0.178
Cognitive_factor	~	medialorbitofrontal_gmv	0.138	0.009	*p<0.001*	0.178
Cognitive_factor	~	middletemporal_gmv	0.205	0.009	*p<0.001*	0.275
Cognitive_factor	~	parahippocampal_gmv	0.166	0.010	*p<0.001*	0.208
Cognitive_factor	~	paracentral_gmv	0.142	0.009	*p<0.001*	0.179
Cognitive_factor	~	parsopercularis_gmv	0.134	0.010	*p<0.001*	0.165
Cognitive_factor	~	parsorbitalis_gmv	0.159	0.010	*p<0.001*	0.202
Cognitive_factor	~	parstriangularis_gmv	0.099	0.009	*p<0.001*	0.123
Cognitive_factor	~	pericalcarine_gmv	0.090	0.009	*p<0.001*	0.123
Cognitive_factor	~	postcentral_gmv	0.168	0.009	*p<0.001*	0.221
Cognitive_factor	~	posteriorcingulate_gmv	0.153	0.010	*p<0.001*	0.189
Cognitive_factor	~	precentral_gmv	0.205	0.009	*p<0.001*	0.269
Cognitive_factor	~	precuneus_gmv	0.159	0.009	*p<0.001*	0.216
Cognitive_factor	~	rostralanteriorcingulate_gmv	0.166	0.010	*p<0.001*	0.200
Cognitive_factor	~	rostralmiddlefrontal_gmv	0.152	0.009	*p<0.001*	0.201
Cognitive_factor	~	superiorfrontal_gmv	0.174	0.009	*p<0.001*	0.233
Cognitive_factor	~	superiorparietal_gmv	0.148	0.009	*p<0.001*	0.197
Cognitive_factor	~	superiortemporal_gmv	0.170	0.009	*p<0.001*	0.225
Cognitive_factor	~	supramarginal_gmv	0.158	0.009	*p<0.001*	0.201
Cognitive_factor	~	frontalpole_gmv	0.114	0.010	*p<0.001*	0.140
Cognitive_factor	~	temporalpole_gmv	0.129	0.010	*p<0.001*	0.160
Cognitive_factor	~	transversetemporal_gmv	0.142	0.010	*p<0.001*	0.179
Cognitive_factor	~	insula_gmv	0.156	0.009	*p<0.001*	0.213

Supplementary Figure 4. Standardized parameter estimates of how the grey matter volume of each region of interest predict the cognitive factor

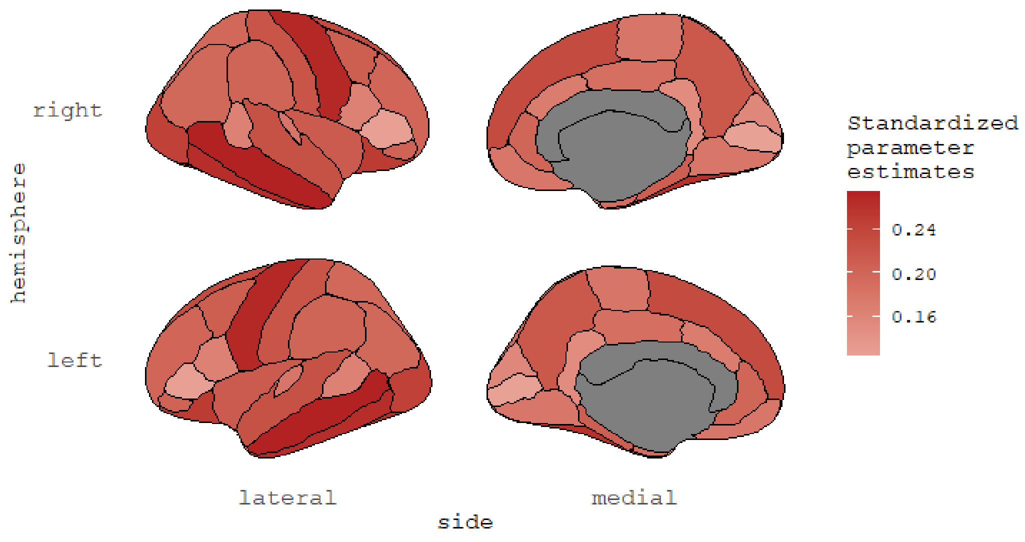

Fractional Anisotropy

Supplementary Table 6. Regression estimates for the model estimating how fractional anisotropy of one region predict the cognitive factor

Path			Estimate	SE	*p*	Standardized Estimate
Cognitive_factor	~	fornix_fa	0.063	0.009	*p<0.001*	0.085
Cognitive_factor	~	cingulatecingulum_fa	0.001	0.009	0.889	0.002
Cognitive_factor	~	parahippocampalcingulum_fa	0.077	0.009	*p<0.001*	0.103
Cognitive_factor	~	corticospinalpyramidal_fa	0.068	0.009	*p<0.001*	0.093
Cognitive_factor	~	anteriorthalamicradiations_fa	0.032	0.009	*p<0.001*	0.043
Cognitive_factor	~	uncinate_fa	0.033	0.009	*p<0.001*	0.044
Cognitive_factor	~	inferiorlongitudinalfasiculus_fa	0.085	0.010	*p<0.001*	0.114
Cognitive_factor	~	inferiorfrontooccipitalfasiculus_fa	0.067	0.009	*p<0.001*	0.092
Cognitive_factor	~	forcepsmajor_fa	0.064	0.009	*p<0.001*	0.093
Cognitive_factor	~	forcepsminor_fa	-0.007	0.008	0.400	-0.010
Cognitive_factor	~	corpuscallosum_fa	0.040	0.009	*p<0.001*	0.059
Cognitive_factor	~	superiorlongitudinalfasiculus_fa	0.102	0.009	*p<0.001*	0.141
Cognitive_factor	~	temporalsuperiorlongitudinalfasiculus_fa	0.094	0.009	*p<0.001*	0.128
Cognitive_factor	~	parietalsuperiorlongitudinalfasiculus_fa	0.104	0.009	*p<0.001*	0.142
Cognitive_factor	~	superiorcorticostriate_fa	0.054	0.009	*p<0.001*	0.073
Cognitive_factor	~	superiorcorticostriatefrontalcortex_fa	0.057	0.009	*p<0.001*	0.078
Cognitive_factor	~	superiorcorticostriateparietalcortex_fa	0.044	0.009	*p<0.001*	0.059

Cognitive_factor	~	striatalinferiorfrontalcortex_fa	0.014	0.009	0.121	0.019
Cognitive_factor	~	inferiorfrontalsuperiorfrontalcortex_fa	0.066	0.009	*p<0.001*	0.090
Cognitive_factor	~	fornix_exfimbria_fa	0.015	0.009	0.102	0.020

Mean Diffusivity

Supplementary Table 7. Regression estimates for the model estimating how mean diffusivity of one region predict the cognitive factor

Path			Estimate	SE	*p*	Standardized Estimate
Cognitive_factor	~	fornix_md	-0.021	0.010	0.038	-0.029
Cognitive_factor	~	cingulatecingulum_md	-0.020	0.010	*0.033*	-0.025
Cognitive_factor	~	parahippocampalcingulum_md	-0.024	0.009	*0.005*	-0.033
Cognitive_factor	~	corticospinalpyramidal_md	-0.014	0.008	0.068	-0.019
Cognitive_factor	~	anteriorthalamicradiations_md	0.007	0.008	0.386	0.010
Cognitive_factor	~	uncinate_md	-0.006	0.007	0.404	-0.008
Cognitive_factor	~	inferiorlongitudinalfasiculus_md	-0.021	0.009	*0.015*	-0.028
Cognitive_factor	~	inferiorfrontooccipitalfasiculus_md	0.003	0.009	0.711	0.004
Cognitive_factor	~	forcepsmajor_md	-0.027	0.010	*0.007*	-0.039
Cognitive_factor	~	forcepsminor_md	0.021	0.008	*0.012*	0.031
Cognitive_factor	~	corpuscallosum_md	-0.013	0.009	0.131	-0.019
Cognitive_factor	~	superiorlongitudinalfasiculus_md	-0.040	0.009	*p<0.001*	-0.056
Cognitive_factor	~	temporalsuperiorlongitudinalfasiculus_md	-0.032	0.009	*p<0.001*	-0.045
Cognitive_factor	~	parietalsuperiorlongitudinalfasiculus_md	-0.044	0.009	*p<0.001*	-0.062
Cognitive_factor	~	superiorcorticostriate_md	-0.024	0.008	*0.004*	-0.033
Cognitive_factor	~	superiorcorticostriatefrontalcortex_md	-0.015	0.008	0.062	-0.022
Cognitive_factor	~	superiorcorticostriateparietalcortex_md	-0.030	0.008	*p<0.001*	-0.042
Cognitive_factor	~	striatalinferiorfrontalcortex_md	0.003	0.009	0.760	0.003
Cognitive_factor	~	inferiorfrontalsuperiorfrontalcortex_md	-0.017	0.009	*0.044*	-0.024
Cognitive_factor	~	fornix_exfimbria_md	-0.007	0.010	0.481	-0.010

White Matter Volume

Supplementary Table 8. Regression estimates for the model estimating how white matter volume of one region predict the cognitive factor

Path			Estimate	SE	*p*	Standardized Estimate
Cognitive_factor	~	fornix_wmv	0.219	0.010	*p<0.001*	0.303
Cognitive_factor	~	cingulatecingulum_wmv	0.124	0.009	*p<0.001*	0.169
Cognitive_factor	~	parahippocampalcingulum_wmv	0.155	0.010	*p<0.001*	0.209
Cognitive_factor	~	corticospinalpyramidal_wmv	0.198	0.010	*p<0.001*	0.274
Cognitive_factor	~	anteriorthalamicradiations_wmv	0.153	0.009	*p<0.001*	0.215
Cognitive_factor	~	uncinate_wmv	0.136	0.009	*p<0.001*	0.187

Cognitive_factor	~	inferiorlongitudinalfasiculus_wmv	0.166	0.010	*p<0.001*	0.226
Cognitive_factor	~	inferiorfrontooccipitalfasiculus_wmv	0.174	0.010	*p<0.001*	0.240
Cognitive_factor	~	forcepsmajor_wmv	0.161	0.009	*p<0.001*	0.230
Cognitive_factor	~	forcepsminor_wmv	0.146	0.009	*p<0.001*	0.209
Cognitive_factor	~	corpuscallosum_wmv	0.179	0.009	*p<0.001*	0.256
Cognitive_factor	~	superiorlongitudinalfasiculus_wmv	0.191	0.010	*p<0.001*	0.261
Cognitive_factor	~	temporalsuperiorlongitudinalfasiculus_wmv	0.197	0.010	*p<0.001*	0.266
Cognitive_factor	~	parietalsuperiorlongitudinalfasiculus_wmv	0.183	0.010	*p<0.001*	0.251
Cognitive_factor	~	superiorcorticostriate_wmv	0.172	0.009	*p<0.001*	0.237
Cognitive_factor	~	superiorcorticostriatefrontalcortex_wmv	0.169	0.009	*p<0.001*	0.234
Cognitive_factor	~	superiorcorticostriateparietalcortex_wmv	0.172	0.009	*p<0.001*	0.233
Cognitive_factor	~	striatalinferiorfrontalcortex_wmv	0.149	0.009	*p<0.001*	0.203
Cognitive_factor	~	inferiorfrontalsuperiorfrontalcortex_wmv	0.181	0.009	*p<0.001*	0.247
Cognitive_factor	~	fornix_exfimbria_wmv	0.201	0.010	*p<0.001*	0.268

Models per metric estimating cognitive factor from several regions in one metric

For every metric, we reported the table for the models with all the regions included as predictors and the models with only the regions that survived the regularization (the one that we used for the analyses).

Cortical Thickness (model with all the regions)

Supplementary Table 9. Regression estimates for the model estimating how cortical thickness of all the regions predict the cognitive factor

Path			Estimate	SE	*p*	Standardized Estimate
Cognitive_factor	~	bankssts_ct	0.018	0.013	0.164	0.021
Cognitive_factor	~	caudalanteriorcingulate_ct	-0.049	0.012	*p<0.001*	-0.058
Cognitive_factor	~	caudalmiddlefrontal_ct	0.025	0.016	0.124	0.032
Cognitive_factor	~	cuneus_ct	0.021	0.014	0.120	0.028
Cognitive_factor	~	entorhinal_ct	0.006	0.010	0.560	0.008
Cognitive_factor	~	fusiform_ct	0.007	0.015	0.641	0.009
Cognitive_factor	~	inferiorparietal_ct	-0.053	0.018	*0.003*	-0.069
Cognitive_factor	~	inferiortemporal_ct	0.023	0.014	0.090	0.030
Cognitive_factor	~	isthmuscingulate_ct	-0.008	0.010	0.436	-0.010
Cognitive_factor	~	lateraloccipital_ct	0.091	0.015	*p<0.001*	0.123
Cognitive_factor	~	lateralorbitofrontal_ct	0.017	0.013	0.184	0.022
Cognitive_factor	~	lingual_ct	0.062	0.013	*p<0.001*	0.082
Cognitive_factor	~	medialorbitofrontal_ct	-0.036	0.012	*0.004*	-0.044
Cognitive_factor	~	middletemporal_ct	0.030	0.015	0.046	0.039
Cognitive_factor	~	parahippocampal_ct	0.076	0.010	*p<0.001*	0.098
Cognitive_factor	~	paracentral_ct	0.002	0.015	0.882	0.003
Cognitive_factor	~	parsopercularis_ct	-0.087	0.014	*p<0.001*	-0.107
Cognitive_factor	~	parsorbitalis_ct	-0.005	0.013	0.670	-0.007
Cognitive_factor	~	parstriangularis_ct	-0.045	0.014	*0.002*	-0.055
Cognitive_factor	~	pericalcarine_ct	-0.007	0.012	0.562	-0.009
Cognitive_factor	~	postcentral_ct	0.055	0.013	*p<0.001*	0.072
Cognitive_factor	~	posteriorcingulate_ct	0.010	0.013	0.429	0.012
Cognitive_factor	~	precentral_ct	0.116	0.016	*p<0.001*	0.152
Cognitive_factor	~	precuneus_ct	-0.024	0.015	0.112	-0.032
Cognitive_factor	~	rostralanteriorcingulate_ct	-0.035	0.012	*0.004*	-0.042
Cognitive_factor	~	rostralmiddlefrontal_ct	0.036	0.017	0.033	0.047
Cognitive_factor	~	superiorfrontal_ct	-0.074	0.017	*p<0.001*	-0.101
Cognitive_factor	~	superiorparietal_ct	-0.087	0.018	*p<0.001*	-0.117
Cognitive_factor	~	superiortemporal_ct	-0.015	0.015	0.321	-0.020
Cognitive_factor	~	supramarginal_ct	-0.009	0.017	0.608	-0.011
Cognitive_factor	~	frontalpole_ct	-0.029	0.012	*0.013*	-0.034
Cognitive_factor	~	temporalpole_ct	0.022	0.012	0.064	0.027
Cognitive_factor	~	transversetemporal_ct	0.020	0.012	0.085	0.025
Cognitive_factor	~	insula_ct	0.014	0.012	0.219	0.018

Supplementary Figure 5. Standardized parameter estimates of how cortical thickness of each region of interest together predict the cognitive factor

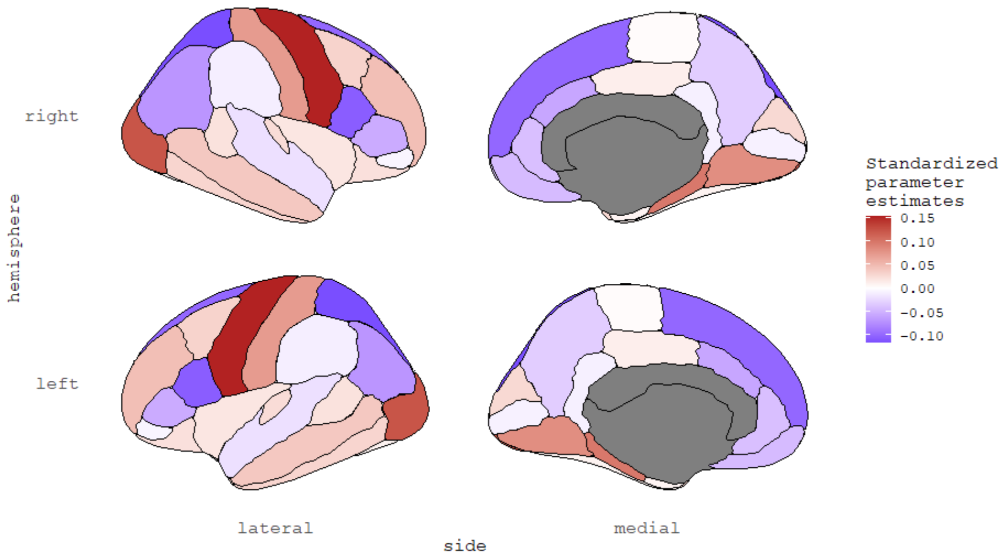

Supplementary Table 10. Comparison of the model with free parameters and the model with parameters constrained to a same value for the cortical thickness model with all the regions. AIC/BIC are two information criterions. Pr(>Chisq): p-value of the chi-square ratio test.

	**Degree of freedom**	**AIC**	**BIC**	**Difference in degree of freedom**	**Pr (>Chisq)**
Free model	141	130758.9	131112.2	NA	NA
Constrained model	174	131314.0	131429.4	33	*p<0.001*

Cortical Thickness (model with the regularized regions)

Supplementary Table 11. Regression estimates for the model estimating how cortical thickness of the regularized regions predict the cognitive factor

**Path**			**Estimate**	**SE**	***p***	**Standardized Estimate**
Cognitive_factor	~	bankssts_ct	0.010	0.013	0.439	0.011
Cognitive_factor	~	caudalanteriorcingulate_ct	-0.046	0.011	*p<0.001*	-0.054
Cognitive_factor	~	entorhinal_ct	0.013	0.010	0.210	0.016
Cognitive_factor	~	inferiortemporal_ct	0.018	0.013	0.164	0.024
Cognitive_factor	~	isthmuscingulate_ct	-0.007	0.010	0.488	-0.009
Cognitive_factor	~	lateraloccipital_ct	0.090	0.014	*p<0.001*	0.121
Cognitive_factor	~	lingual_ct	0.071	0.012	*p<0.001*	0.094
Cognitive_factor	~	middletemporal_ct	0.016	0.014	0.242	0.021
Cognitive_factor	~	parahippocampal_ct	0.074	0.010	*p<0.001*	0.095

Cognitive_factor	~	paracentral_ct	0.009	0.014	0.553	0.011
Cognitive_factor	~	parsopercularis_ct	-0.085	0.013	*p<0.001*	-0.105
Cognitive_factor	~	parstriangularis_ct	-0.051	0.014	*p<0.001*	-0.063
Cognitive_factor	~	pericalcarine_ct	0.002	0.012	0.852	0.003
Cognitive_factor	~	postcentral_ct	0.057	0.012	*p<0.001*	0.075
Cognitive_factor	~	precentral_ct	0.128	0.015	*p<0.001*	0.167
Cognitive_factor	~	precuneus_ct	-0.032	0.015	*0.031*	-0.042
Cognitive_factor	~	rostralanteriorcingulate_ct	-0.042	0.011	*p<0.001*	-0.049
Cognitive_factor	~	rostralmiddlefrontal_ct	0.023	0.016	0.139	0.031
Cognitive_factor	~	superiorfrontal_ct	-0.074	0.016	*p<0.001*	-0.099
Cognitive_factor	~	superiorparietal_ct	-0.107	0.016	*p<0.001*	-0.144
Cognitive_factor	~	temporalpole_ct	0.021	0.011	0.060	0.027
Cognitive_factor	~	transversetemporal_ct	0.016	0.011	0.146	0.020

Supplementary Figure 6. Standardized parameter estimates of how the cortical thickness of each regularized region of interest together predict the cognitive factor

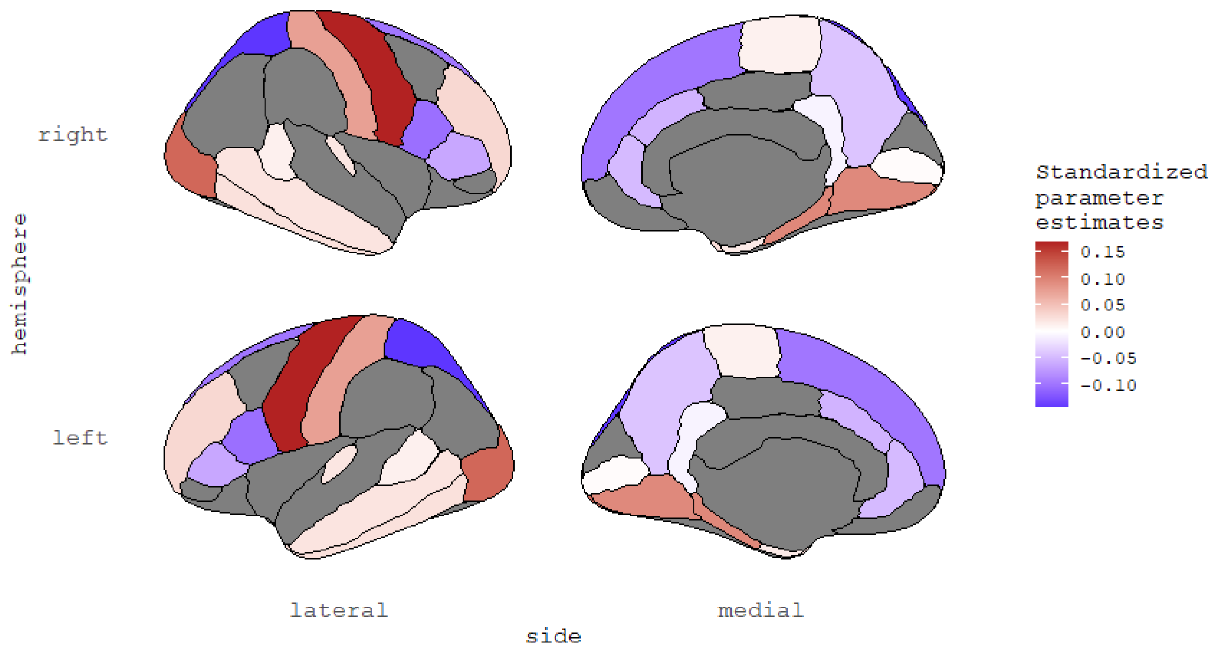

Supplementary Table 12. Comparison of the model with free parameters and the model with parameters constrained to a same value for the cortical thickness model with the regularized regions. AIC/BIC are two information criterions. Pr(>Chisq): p-value of the chi-square ratio test.

	**Degree of freedom**	**AIC**	**BIC**	**Difference in degree of freedom**	**Pr (>Chisq)**
Free model	93	130773.4	131040.1	NA	NA
Constrained model	114	131299.2	131414.5	21	*p<0.001*

Surface Area (model with all the regions)

Supplementary Table 13. Regression estimates for the model estimating how surface area of all the regions predict the cognitive factor

Path			Estimate	SE	*p*	Standardized Estimate
Cognitive_factor	~	bankssts_sa	-0.038	0.016	*0.014*	-0.047
Cognitive_factor	~	caudalanteriorcingulate_sa	0.041	0.017	*0.018*	0.046
Cognitive_factor	~	caudalmiddlefrontal_sa	0.032	0.015	*0.036*	0.041
Cognitive_factor	~	cuneus_sa	-0.025	0.018	0.151	-0.033
Cognitive_factor	~	entorhinal_sa	0.071	0.012	*p<0.001*	0.089
Cognitive_factor	~	fusiform_sa	0.054	0.017	*0.001*	0.071
Cognitive_factor	~	inferiorparietal_sa	-0.028	0.015	0.070	-0.035
Cognitive_factor	~	inferiortemporal_sa	0.090	0.020	*p<0.001*	0.118
Cognitive_factor	~	isthmuscingulate_sa	-0.040	0.015	*0.006*	-0.051
Cognitive_factor	~	lateraloccipital_sa	0.010	0.015	0.514	0.013
Cognitive_factor	~	lateralorbitofrontal_sa	0.059	0.017	*0.001*	0.079
Cognitive_factor	~	lingual_sa	0.030	0.017	0.077	0.039
Cognitive_factor	~	medialorbitofrontal_sa	0.001	0.016	0.967	0.001
Cognitive_factor	~	middletemporal_sa	0.013	0.023	0.568	0.018
Cognitive_factor	~	parahippocampal_sa	-0.001	0.012	0.955	-0.001
Cognitive_factor	~	paracentral_sa	-0.040	0.014	*0.004*	-0.051
Cognitive_factor	~	parsopercularis_sa	0.045	0.014	*0.002*	0.056
Cognitive_factor	~	parsorbitalis_sa	0.035	0.016	*0.032*	0.045
Cognitive_factor	~	parstriangularis_sa	-0.062	0.016	*p<0.001*	-0.078
Cognitive_factor	~	pericalcarine_sa	-0.004	0.018	0.828	-0.005
Cognitive_factor	~	postcentral_sa	-0.043	0.017	*0.011*	-0.057
Cognitive_factor	~	posteriorcingulate_sa	0.005	0.017	0.743	0.007
Cognitive_factor	~	precentral_sa	0.029	0.017	0.084	0.039
Cognitive_factor	~	precuneus_sa	0.020	0.016	0.202	0.027
Cognitive_factor	~	rostralanteriorcingulate_sa	0.008	0.016	0.634	0.010
Cognitive_factor	~	rostralmiddlefrontal_sa	-0.013	0.016	0.421	-0.017
Cognitive_factor	~	superiorfrontal_sa	0.024	0.018	0.195	0.032
Cognitive_factor	~	superiorparietal_sa	0.021	0.015	0.160	0.028
Cognitive_factor	~	superiortemporal_sa	0.016	0.021	0.442	0.021
Cognitive_factor	~	supramarginal_sa	-0.008	0.015	0.620	-0.010
Cognitive_factor	~	frontalpole_sa	0.050	0.014	*p<0.001*	0.062
Cognitive_factor	~	temporalpole_sa	-0.096	0.014	*p<0.001*	-0.121
Cognitive_factor	~	transversetemporal_sa	-0.033	0.015	*0.029*	-0.041
Cognitive_factor	~	insula_sa	0.039	0.015	*0.007*	0.052

Supplementary Figure 7. Standardized parameter estimates of how the surface area of each region of interest together predict the cognitive factor

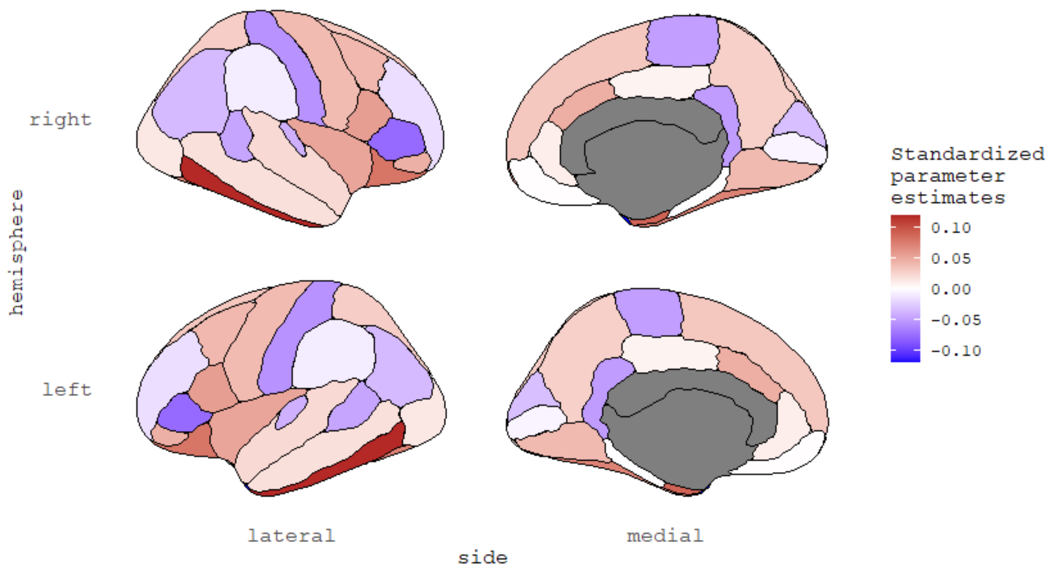

Supplementary Table 14. Comparison of the model with free parameters and the model with parameters constrained to a same value for the surface area model with all the regions. AIC/BIC are two information criterions. Pr(>Chisq): p-value of the chi-square ratio test.

	Degree of freedom	AIC	BIC	Difference in degree of freedom	Pr (>Chisq)
Free model	141	130573.0	130926.3	NA	NA
Constrained model	174	130779.8	130895.1	33	*p<0.001*

Surface Area (model with the regularized regions)

Supplementary Table 15. Regression estimates for the model estimating how surface area of the regularized regions predict the cognitive factor

Path			Estimate	SE	*p*	Standardized Estimate
Cognitive_factor	~	bankssts_sa	-0.049	0.014	*0.001*	-0.060
Cognitive_factor	~	caudalmiddlefrontal_sa	0.035	0.014	*0.015*	0.044
Cognitive_factor	~	cuneus_sa	-0.014	0.016	0.366	-0.019
Cognitive_factor	~	entorhinal_sa	0.071	0.012	*p<0.001*	0.089
Cognitive_factor	~	fusiform_sa	0.068	0.016	*p<0.001*	0.089
Cognitive_factor	~	inferiortemporal_sa	0.088	0.020	*p<0.001*	0.116
Cognitive_factor	~	isthmuscingulate_sa	-0.035	0.014	*0.011*	-0.044
Cognitive_factor	~	lateralorbitofrontal_sa	0.074	0.014	*p<0.001*	0.099
Cognitive_factor	~	middletemporal_sa	0.020	0.021	0.337	0.027
Cognitive_factor	~	parahippocampal_sa	0.003	0.012	0.828	0.003
Cognitive_factor	~	paracentral_sa	-0.034	0.013	*0.010*	-0.043
Cognitive_factor	~	parsopercularis_sa	0.045	0.014	*0.001*	0.055
Cognitive_factor	~	parstriangularis_sa	-0.045	0.014	*0.001*	-0.056

Cognitive_factor	~	pericalcarine_sa	0.019	0.014	0.181	0.026
Cognitive_factor	~	posteriorcingulate_sa	0.016	0.015	0.292	0.020
Cognitive_factor	~	precentral_sa	0.023	0.015	0.135	0.030
Cognitive_factor	~	rostralanteriorcingulate_sa	0.036	0.014	*0.011*	0.044
Cognitive_factor	~	temporalpole_sa	-0.088	0.013	*p<0.001*	-0.110
Cognitive_factor	~	insula_sa	0.031	0.014	*0.022*	0.041

Supplementary Figure 8. Standardized parameter estimates of how the surface area of each regularized region of interest together predict the cognitive factor

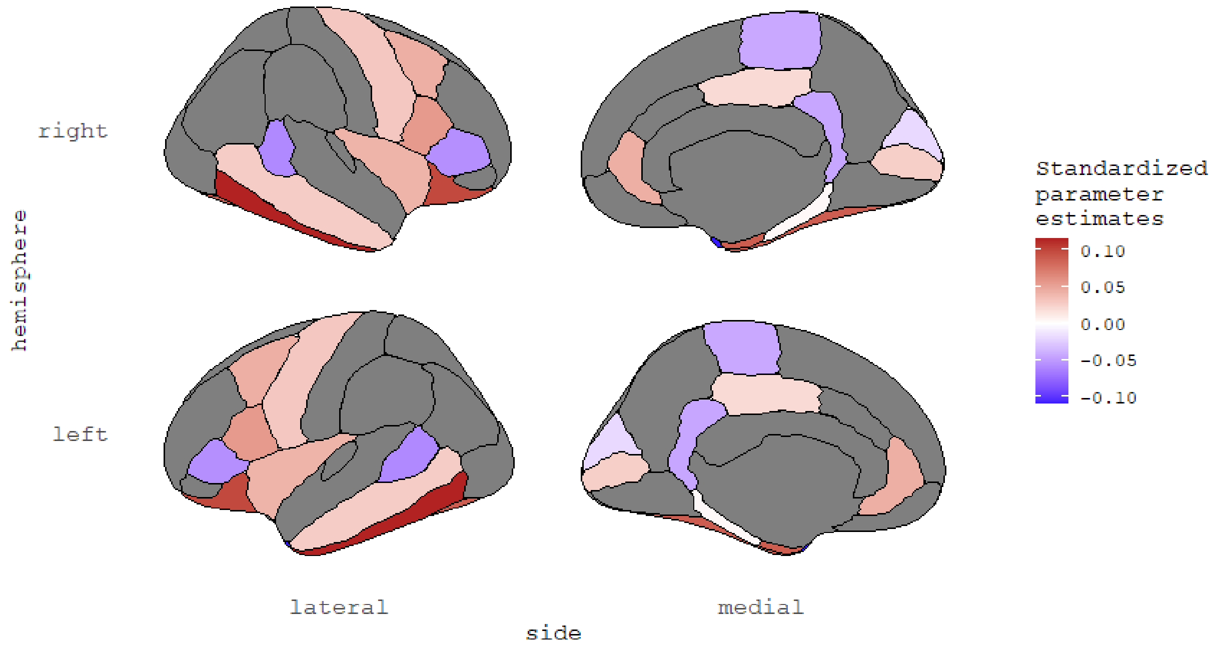

Supplementary Table 16. Comparison of the model with free parameters and the model with parameters constrained to a same value for the surface area model with the regularized regions. AIC/BIC are two information criterions. Pr(>Chisq): p-value of the chi-square ratio test.

	**Degree of freedom**	**AIC**	**BIC**	**Difference in degree of freedom**	**Pr (>Chisq)**
Free model	81	130604.7	130849.9	NA	NA
Constrained model	99	130780.3	130895.6	18	*p<0.001*

Grey Matter Volume (model with all the regions)

Supplementary Table 17. Regression estimates for the model estimating how grey matter volume of all the regions predict the cognitive factor

Path			Estimate	SE	*p*	Standardized Estimate
Cognitive_factor	~	bankssts_gmv	-0.038	0.014	*0.008*	-0.045
Cognitive_factor	~	caudalanteriorcingulate_gmv	0.029	0.015	0.063	0.031
Cognitive_factor	~	caudalmiddlefrontal_gmv	0.019	0.013	0.152	0.024
Cognitive_factor	~	cuneus_gmv	-0.012	0.016	0.445	-0.016
Cognitive_factor	~	entorhinal_gmv	0.047	0.012	*p<0.001*	0.058
Cognitive_factor	~	fusiform_gmv	0.054	0.014	*p<0.001*	0.070
Cognitive_factor	~	inferiorparietal_gmv	-0.028	0.014	*0.050*	-0.036
Cognitive_factor	~	inferiortemporal_gmv	0.035	0.015	*0.020*	0.045
Cognitive_factor	~	isthmuscingulate_gmv	-0.064	0.014	*p<0.001*	-0.080
Cognitive_factor	~	lateraloccipital_gmv	0.047	0.014	*0.001*	0.061
Cognitive_factor	~	lateralorbitofrontal_gmv	0.064	0.017	*p<0.001*	0.086
Cognitive_factor	~	lingual_gmv	0.010	0.014	0.473	0.014
Cognitive_factor	~	medialorbitofrontal_gmv	-0.074	0.015	*p<0.001*	-0.095
Cognitive_factor	~	middletemporal_gmv	0.134	0.016	*p<0.001*	0.178
Cognitive_factor	~	parahippocampal_gmv	0.063	0.011	*p<0.001*	0.077
Cognitive_factor	~	paracentral_gmv	-0.030	0.013	*0.025*	-0.037
Cognitive_factor	~	parsopercularis_gmv	0.013	0.013	0.299	0.016
Cognitive_factor	~	parsorbitalis_gmv	0.014	0.014	0.324	0.017
Cognitive_factor	~	parstriangularis_gmv	-0.035	0.014	*0.010*	-0.043
Cognitive_factor	~	pericalcarine_gmv	0.009	0.015	0.555	0.012
Cognitive_factor	~	postcentral_gmv	-0.022	0.014	0.106	-0.029
Cognitive_factor	~	posteriorcingulate_gmv	-0.024	0.014	0.095	-0.029
Cognitive_factor	~	precentral_gmv	0.082	0.015	*p<0.001*	0.107
Cognitive_factor	~	precuneus_gmv	0.005	0.016	0.769	0.006
Cognitive_factor	~	rostralanteriorcingulate_gmv	-0.006	0.015	0.691	-0.007
Cognitive_factor	~	rostralmiddlefrontal_gmv	-0.013	0.014	0.363	-0.017
Cognitive_factor	~	superiorfrontal_gmv	0.011	0.016	0.502	0.015
Cognitive_factor	~	superiorparietal_gmv	0.015	0.014	0.294	0.020
Cognitive_factor	~	superiortemporal_gmv	-0.010	0.016	0.546	-0.013
Cognitive_factor	~	supramarginal_gmv	-0.006	0.014	0.668	-0.008
Cognitive_factor	~	frontalpole_gmv	-0.008	0.012	0.499	-0.010
Cognitive_factor	~	temporalpole_gmv	0.002	0.012	0.860	0.003
Cognitive_factor	~	transversetemporal_gmv	0.025	0.013	0.063	0.031
Cognitive_factor	~	insula_gmv	0.005	0.014	0.729	0.007

Supplementary Figure 9. Standardized parameter estimates of how the grey matter volume of each region of interest together predict the cognitive factor

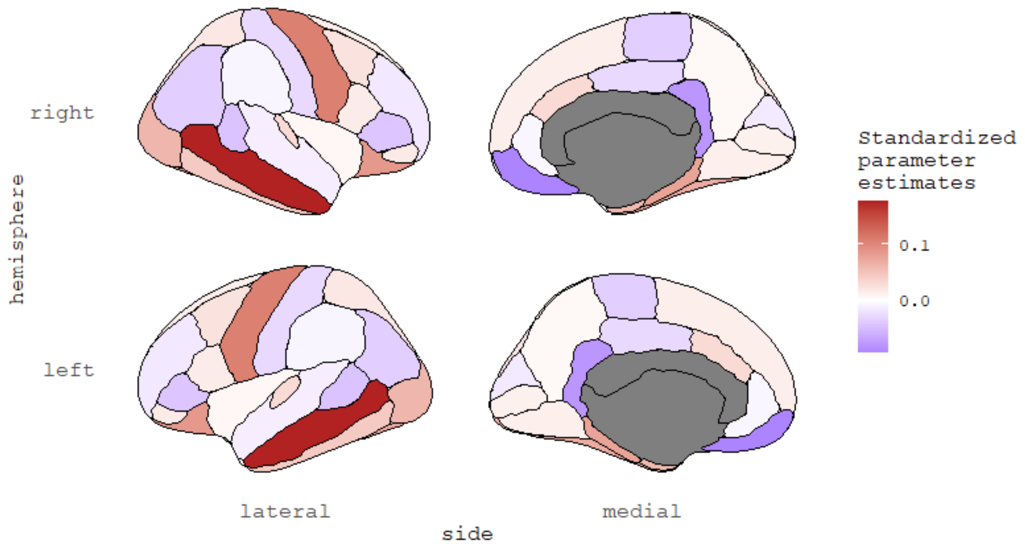

Supplementary Table 18. Comparison of the model with free parameters and the model with parameters constrained to a same value for the grey matter volume model with all the regions. AIC/BIC are two information criterions. Pr(>Chisq): p-value of the chi-square ratio test.

	Degree of freedom	AIC	BIC	Difference in degree of freedom	Pr (>Chisq)
Free model	141	130425.7	130779.0	NA	NA
Constrained model	174	130689.0	130804.4	33	*p<0.001*

Grey Matter Volume (model with the regularized regions)

Supplementary Table 19. Regression estimates for the model estimating how grey matter volume of the regularized regions predict the cognitive factor

Path			Estimate	SE	*p*	Standardized Estimate
Cognitive_factor	~	cuneus_gmv	0.023	0.010	*0.021*	0.030
Cognitive_factor	~	entorhinal_gmv	0.056	0.011	*p<0.001*	0.069
Cognitive_factor	~	fusiform_gmv	0.060	0.013	*p<0.001*	0.077
Cognitive_factor	~	isthmuscingulate_gmv	-0.054	0.012	*p<0.001*	-0.068
Cognitive_factor	~	middletemporal_gmv	0.133	0.013	*p<0.001*	0.177
Cognitive_factor	~	parahippocampal_gmv	0.065	0.011	*p<0.001*	0.080
Cognitive_factor	~	parstriangularis_gmv	-0.016	0.011	0.144	-0.019
Cognitive_factor	~	posteriorcingulate_gmv	-0.024	0.013	0.057	-0.029
Cognitive_factor	~	precentral_gmv	0.091	0.012	*p<0.001*	0.118

Cognitive_factor	~	superiortemporal_gmv	-0.031	0.015	*0.034*	-0.041
Cognitive_factor	~	transversetemporal_gmv	0.035	0.013	*0.005*	0.044

Supplementary Figure 10. Standardized parameter estimates of how the grey matter volume of each regularized region of interest together predict the cognitive factor

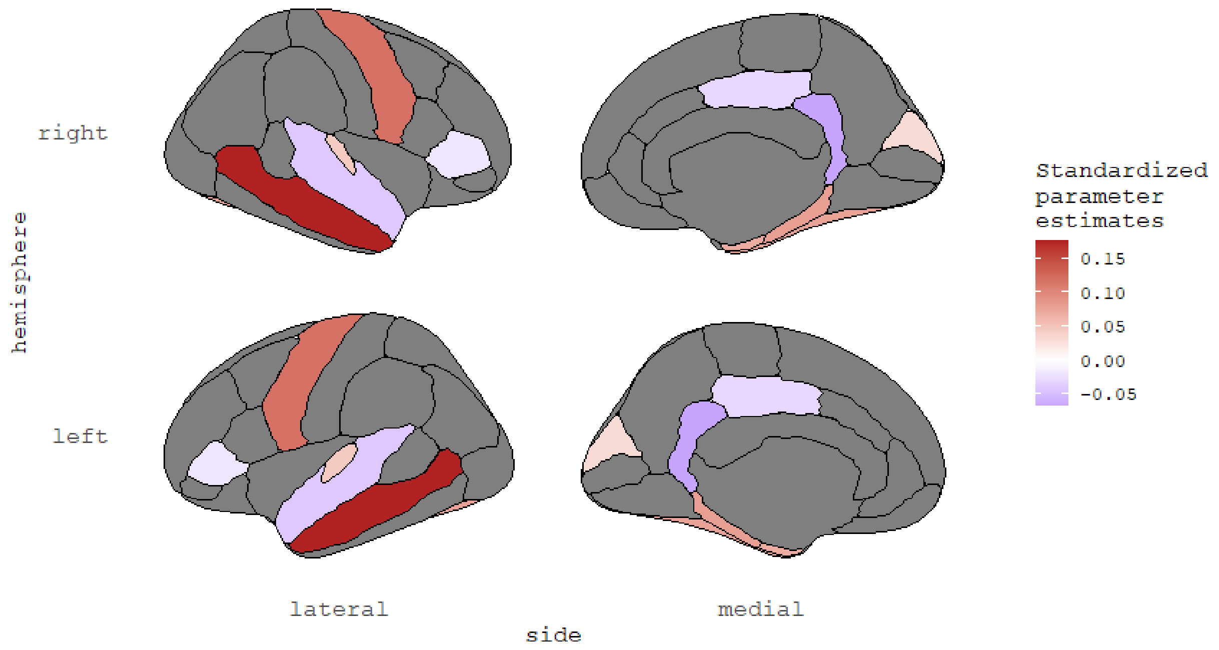

Supplementary Table 20. Comparison of the model with free parameters and the model with parameters constrained to a same value for the grey matter volume model with the regularized regions. AIC/BIC are two information criterions. Pr(>Chisq): p-value of the chi-square ratio test.

	**Degree of freedom**	**AIC**	**BIC**	**Difference in degree of freedom**	**Pr (>Chisq)**
Free model	49	130476.4	130663.8	NA	NA
Constrained model	59	130666.9	130782.3	10	*p<0.001*

Fractional Anisotropy (model with all the regions)

Supplementary Table 21. Regression estimates for the model estimating how fractional anisotropy of all the regions predict the cognitive factor

Path			Estimate	SE	*p*	Standardized Estimate
Cognitive_factor	~	fornix_fa	0.092	0.016	*p<0.001*	0.120
Cognitive_factor	~	cingulatecingulum_fa	-0.091	0.012	*p<0.001*	-0.119
Cognitive_factor	~	parahippocampalcingulum_fa	0.046	0.012	*p<0.001*	0.061
Cognitive_factor	~	corticospinalpyramidal_fa	0.084	0.017	*p<0.001*	0.113
Cognitive_factor	~	anteriorthalamicradiations_fa	0.008	0.013	0.545	0.011
Cognitive_factor	~	uncinate_fa	-0.047	0.017	*0.007*	-0.062
Cognitive_factor	~	inferiorlongitudinalfasiculus_fa	0.021	0.016	0.181	0.027
Cognitive_factor	~	inferiorfrontooccipitalfasiculus_fa	0.053	0.018	*0.003*	0.071
Cognitive_factor	~	forcepsmajor_fa	0.038	0.018	*0.035*	0.054
Cognitive_factor	~	forcepsminor_fa	-0.107	0.020	*p<0.001*	-0.153
Cognitive_factor	~	corpuscallosum_fa	0.012	0.031	0.696	0.017
Cognitive_factor	~	superiorlongitudinalfasiculus_fa	0.316	0.136	*0.020*	0.424
Cognitive_factor	~	temporalsuperiorlongitudinalfasiculus_fa	-0.195	0.073	*0.007*	-0.260
Cognitive_factor	~	parietalsuperiorlongitudinalfasiculus_fa	0.035	0.079	0.659	0.046
Cognitive_factor	~	superiorcorticostriate_fa	0.879	0.116	*p<0.001*	1.162
Cognitive_factor	~	superiorcorticostriatefrontalcortex_fa	-0.243	0.051	*p<0.001*	-0.323
Cognitive_factor	~	superiorcorticostriateparietalcortex_fa	-0.732	0.081	*p<0.001*	-0.958
Cognitive_factor	~	striatalinferiorfrontalcortex_fa	-0.080	0.016	*p<0.001*	-0.104
Cognitive_factor	~	inferiorfrontalsuperiorfrontalcortex_fa	0.013	0.019	0.486	0.017
Cognitive_factor	~	fornix_exfimbria_fa	-0.071	0.016	*p<0.001*	-0.090

Supplementary Table 22. Comparison of the model with free parameters and the model with parameters constrained to a same value for the fractional anisotropy model with all the regions. AIC/BIC are two information criterions. Pr(>Chisq): p-value of the chi-square ratio test.

	Degree of freedom	AIC	BIC	Difference in degree of freedom	Pr (>Chisq)
**Free model**	85	123512.8	123763.3	NA	NA
**Constrained model**	104	123949.8	124064.3	19	*p<0.001*

Fractional Anisotropy (model with the regularized regions)

Supplementary Table 23. Regression estimates for the model estimating how fractional anisotropy of the regularized regions predict the cognitive factor

Path			Estimate	SE	*p*	Standardized Estimate
Cognitive_factor	~	forcepsmajor_fa	0.039	0.012	*0.001*	0.056
Cognitive_factor	~	forcepsminor_fa	-0.144	0.012	*p<0.001*	-0.206
Cognitive_factor	~	fornix_fa	0.022	0.011	*0.041*	0.029
Cognitive_factor	~	parahippocampalcingulum_fa	0.042	0.011	*p<0.001*	0.057
Cognitive_factor	~	corticospinalpyramidal_fa	0.018	0.012	0.153	0.024
Cognitive_factor	~	inferiorlongitudinalfasiculus_fa	0.018	0.013	0.189	0.023
Cognitive_factor	~	superiorlongitudinalfasiculus_fa	0.128	0.013	*p<0.001*	0.173

Supplementary Table 24. Comparison of the model with free parameters and the model with parameters constrained to a same value for the fractional anisotropy model with the regularized regions. AIC/BIC are two information criterions. Pr(>Chisq): p-value of the chi-square ratio test.

	Degree of freedom	AIC	BIC	Difference in degree of freedom	Pr (>Chisq)
**Free model**	33	123715.0	123872.4	NA	NA
**Constrained model**	39	123924.8	124039.2	6	*p<0.001*

Mean Diffusivity (model with all the regions)

Supplementary Table 25. Regression estimates for the model estimating how mean diffusivity of all the regions predict the cognitive factor

Path			Estimate	SE	*p*	Standardized Estimate
Cognitive_factor	~	fornix_md	-0.070	0.023	0.002	-0.095
Cognitive_factor	~	cingulatecingulum_md	0.022	0.016	0.175	0.025
Cognitive_factor	~	parahippocampalcingulum_md	-0.029	0.015	*0.049*	-0.039
Cognitive_factor	~	corticospinalpyramidal_md	0.009	0.016	0.569	0.012
Cognitive_factor	~	anteriorthalamicradiations_md	-0.003	0.018	0.864	-0.004
Cognitive_factor	~	uncinate_md	-0.018	0.019	0.361	-0.024
Cognitive_factor	~	inferiorlongitudinalfasiculus_md	-0.061	0.021	*0.003*	-0.079
Cognitive_factor	~	inferiorfrontooccipitalfasiculus_md	0.119	0.024	*p<0.001*	0.153
Cognitive_factor	~	forcepsmajor_md	-0.013	0.018	0.456	-0.019
Cognitive_factor	~	forcepsminor_md	0.085	0.018	*p<0.001*	0.123
Cognitive_factor	~	corpuscallosum_md	-0.060	0.026	*0.019*	-0.084
Cognitive_factor	~	superiorlongitudinalfasiculus_md	-0.647	0.195	*0.001*	-0.865

Cognitive_factor	~	temporalsuperiorlongitudinalfasiculus_md	0.386	0.103	*p<0.001*	0.517
Cognitive_factor	~	parietalsuperiorlongitudinalfasiculus_md	0.179	0.109	0.102	0.240
Cognitive_factor	~	superiorcorticostriate_md	0.286	0.149	0.055	0.374
Cognitive_factor	~	superiorcorticostriatefrontalcortex_md	0.008	0.070	0.907	0.011
Cognitive_factor	~	superiorcorticostriateparietalcortex_md	-0.306	0.103	*0.003*	-0.400
Cognitive_factor	~	striatalinferiorfrontalcortex_md	-0.044	0.025	0.078	-0.055
Cognitive_factor	~	inferiorfrontalsuperiorfrontalcortex_md	0.063	0.031	*0.038*	0.084
Cognitive_factor	~	fornix_exfimbria_md	0.075	0.021	*p<0.001*	0.101

Supplementary Table 26. Comparison of the model with free parameters and the model with parameters constrained to a same value for the mean diffusivity model with all the regions. AIC/BIC are two information criterions. Pr(>Chisq): p-value of the chi-square ratio test.

	Degree of freedom	AIC	BIC	Difference in degree of freedom	Pr (>Chisq)
**Free model**	85	123885.5	124135.9	NA	NA
**Constrained model**	104	124018.4	124132.8	19	*p<0.001*

Mean Diffusivity (model with the regularized regions)

Supplementary Table 27. Regression estimates for the model estimating how mean diffusivity of the regularized regions predict the cognitive factor

Path			Estimate	SE	*p*	Standardized Estimate
Cognitive_factor	~	forcepsmajor_md	-0.030	0.013	*0.025*	-0.043
Cognitive_factor	~	forcepsminor_md	0.055	0.012	*p<0.001*	0.080
Cognitive_factor	~	cingulatecingulum_md	0.024	0.015	0.118	0.029
Cognitive_factor	~	corticospinalpyramidal_md	-0.010	0.014	0.456	-0.014
Cognitive_factor	~	parietalsuperiorlongitudinalfasiculus_md	-0.067	0.016	*p<0.001*	-0.093
Cognitive_factor	~	fornix_exfimbria_md	0.002	0.010	0.827	0.003

Supplementary Table 28. Comparison of the model with free parameters and the model with parameters constrained to a same value for the mean diffusivity model with the regularized regions. AIC/BIC are two information criterions. Pr(>Chisq): p-value of the chi-square ratio test.

	Degree of freedom	AIC	BIC	Difference in degree of freedom	Pr (>Chisq)
**Free model**	29	123990.0	124140.3	NA	NA
**Constrained model**	34	124040.9	124155.4	5	*p<0.001*

White Matter Volume (model with all the regions)

Supplementary Table 29. Regression estimates for the model estimating how white matter volume of all the regions predict the cognitive factor

Path			Estimate	SE	*p*	Standardized Estimate
Cognitive_factor	~	fornix_wmv	0.174	0.028	*p<0.001*	0.240
Cognitive_factor	~	cingulatecingulum_wmv	-0.084	0.016	*p<0.001*	-0.112
Cognitive_factor	~	parahippocampalcingulum_wmv	0.002	0.013	0.879	0.003
Cognitive_factor	~	corticospinalpyramidal_wmv	0.210	0.024	*p<0.001*	0.287
Cognitive_factor	~	anteriorthalamicradiations_wmv	0.074	0.024	*0.002*	0.103
Cognitive_factor	~	uncinate_wmv	-0.029	0.016	0.073	-0.039
Cognitive_factor	~	inferiorlongitudinalfasiculus_wmv	0.020	0.017	0.245	0.026
Cognitive_factor	~	inferiorfrontooccipitalfasiculus_wmv	0.015	0.027	0.569	0.021
Cognitive_factor	~	forcepsmajor_wmv	-0.027	0.019	0.158	-0.038
Cognitive_factor	~	forcepsminor_wmv	-0.079	0.026	*0.002*	-0.112
Cognitive_factor	~	corpuscallosum_wmv	0.095	0.040	*0.017*	0.134
Cognitive_factor	~	superiorlongitudinalfasiculus_wmv	-0.020	0.081	0.809	-0.027
Cognitive_factor	~	temporalsuperiorlongitudinalfasiculus_wmv	0.113	0.054	*0.037*	0.151
Cognitive_factor	~	parietalsuperiorlongitudinalfasiculus_wmv	-0.017	0.043	0.691	-0.023
Cognitive_factor	~	superiorcorticostriate_wmv	-0.082	0.087	0.348	-0.111
Cognitive_factor	~	superiorcorticostriatefrontalcortex_wmv	-0.153	0.051	*0.003*	-0.209
Cognitive_factor	~	superiorcorticostriateparietalcortex_wmv	-0.009	0.056	0.870	-0.012
Cognitive_factor	~	striatalinferiorfrontalcortex_wmv	-0.050	0.019	*0.008*	-0.067
Cognitive_factor	~	inferiorfrontalsuperiorfrontalcortex_wmv	0.101	0.023	*p<0.001*	0.136
Cognitive_factor	~	fornix_exfimbria_wmv	-0.034	0.027	0.204	-0.045

Supplementary Table 30. Comparison of the model with free parameters and the model with parameters constrained to a same value for the white matter volume model with all the regions. AIC/BIC are two information criterions. Pr(>Chisq): p-value of the chi-square ratio test.

	Degree of freedom	AIC	BIC	Difference in degree of freedom	Pr (>Chisq)
**Free model**	85	123223.1	123473.5	NA	NA
**Constrained model**	104	123498.9	123613.4	19	*p<0.001*

White Matter Volume (model with the regularized regions)

Supplementary Table 31. Regression estimates for the model estimating how white matter volume of the regularized regions predict the cognitive factor

Path			Estimate	SE	*p*	Standardized Estimate
Cognitive_factor	~	forcepsmajor_wmv	0.008	0.013	0.550	0.011
Cognitive_factor	~	fornix_wmv	0.150	0.016	*p<0.001*	0.207
Cognitive_factor	~	cingulatecingulum_wmv	-0.089	0.015	*p<0.001*	-0.118
Cognitive_factor	~	parahippocampalcingulum_wmv	0.004	0.012	0.719	0.006
Cognitive_factor	~	corticospinalpyramidal_wmv	0.117	0.017	*p<0.001*	0.160
Cognitive_factor	~	anteriorthalamicradiations_wmv	-0.007	0.018	0.715	-0.009
Cognitive_factor	~	uncinate_wmv	-0.029	0.015	0.057	-0.039
Cognitive_factor	~	inferiorlongitudinalfasiculus_wmv	0.028	0.016	0.080	0.038
Cognitive_factor	~	inferiorfrontooccipitalfasiculus_wmv	-0.018	0.025	0.477	-0.024
Cognitive_factor	~	temporalsuperiorlongitudinalfasiculus_wmv	0.067	0.017	*p<0.001*	0.091

Supplementary Table 32. Comparison of the model with free parameters and the model with parameters constrained to a same value for the white matter volume model with the regularized regions. AIC/BIC are two information criterions. Pr(>Chisq): p-value of the chi-square ratio test.

	Degree of freedom	AIC	BIC	Difference in degree of freedom	Pr (>Chisq)
**Free model**	45	123280.8	123459.7	NA	NA
**Constrained model**	54	123486.0	123600.5	9	*p<0.001*

Models per tissue estimating cognitive factor from several regions in three metrics

For grey and white matter, we reported the tables for the models with all the regions included as predictors and the models with only the regions that survived the regularization (the one that we used for the analyses).

Grey matter metrics (model with all the regions)

Supplementary Table 33. Regression estimates for the model estimating how cortical thickness, surface area and grey matter volume of all the regions predict the cognitive factor

Path			Estimate	SE	*p*	Standardized Estimate
Cognitive_factor	~	bankssts_ct	-0.002	0.018	0.911	-0.002
Cognitive_factor	~	caudalanteriorcingulate_ct	-0.045	0.019	*0.015*	-0.052
Cognitive_factor	~	caudalmiddlefrontal_ct	0.067	0.029	*0.022*	0.085
Cognitive_factor	~	cuneus_ct	0.010	0.030	0.753	0.012
Cognitive_factor	~	entorhinal_ct	0.021	0.019	0.273	0.025
Cognitive_factor	~	fusiform_ct	0.009	0.025	0.723	0.011
Cognitive_factor	~	inferiorparietal_ct	-0.037	0.032	0.249	-0.047
Cognitive_factor	~	inferiortemporal_ct	0.060	0.029	*0.039*	0.076
Cognitive_factor	~	isthmuscingulate_ct	0.016	0.019	0.404	0.019
Cognitive_factor	~	lateraloccipital_ct	0.025	0.037	0.500	0.033
Cognitive_factor	~	lateralorbitofrontal_ct	-0.079	0.022	*p<0.001*	-0.098
Cognitive_factor	~	lingual_ct	0.063	0.032	*0.046*	0.081
Cognitive_factor	~	medialorbitofrontal_ct	0.054	0.023	*0.020*	0.066
Cognitive_factor	~	middletemporal_ct	-0.059	0.034	0.080	-0.076
Cognitive_factor	~	parahippocampal_ct	0.037	0.028	0.184	0.047
Cognitive_factor	~	paracentral_ct	0.011	0.027	0.697	0.013
Cognitive_factor	~	parsopercularis_ct	-0.044	0.027	0.100	-0.053
Cognitive_factor	~	parsorbitalis_ct	0.014	0.023	0.544	0.017
Cognitive_factor	~	parstriangularis_ct	-0.043	0.027	0.117	-0.051
Cognitive_factor	~	pericalcarine_ct	0.100	0.029	*0.001*	0.128
Cognitive_factor	~	postcentral_ct	0.048	0.036	0.181	0.063
Cognitive_factor	~	posteriorcingulate_ct	-0.048	0.022	*0.028*	-0.057
Cognitive_factor	~	precentral_ct	0.173	0.038	*p<0.001*	0.223
Cognitive_factor	~	precuneus_ct	-0.064	0.026	*0.013*	-0.083
Cognitive_factor	~	rostralanteriorcingulate_ct	-0.053	0.020	*0.006*	-0.061
Cognitive_factor	~	rostralmiddlefrontal_ct	0.114	0.030	*p<0.001*	0.146
Cognitive_factor	~	superiorfrontal_ct	-0.004	0.032	0.908	-0.005
Cognitive_factor	~	superiorparietal_ct	-0.098	0.034	*0.004*	-0.130
Cognitive_factor	~	superiortemporal_ct	-0.074	0.035	*0.035*	-0.095
Cognitive_factor	~	supramarginal_ct	0.051	0.032	0.106	0.065
Cognitive_factor	~	frontalpole_ct	-0.001	0.022	0.979	-0.001
Cognitive_factor	~	temporalpole_ct	-0.042	0.021	*0.048*	-0.051
Cognitive_factor	~	transversetemporal_ct	-0.056	0.019	*0.003*	-0.067

Cognitive_factor	~	insula_ct	-0.047	0.027	0.079	-0.058
Cognitive_factor	~	bankssts_sa	-0.060	0.046	0.189	-0.072
Cognitive_factor	~	caudalanteriorcingulate_sa	-0.025	0.054	0.642	-0.028
Cognitive_factor	~	caudalmiddlefrontal_sa	0.219	0.070	*0.002*	0.275
Cognitive_factor	~	cuneus_sa	-0.034	0.048	0.475	-0.045
Cognitive_factor	~	entorhinal_sa	0.022	0.032	0.493	0.027
Cognitive_factor	~	fusiform_sa	0.101	0.056	0.069	0.131
Cognitive_factor	~	inferiorparietal_sa	0.002	0.069	0.973	0.003
Cognitive_factor	~	inferiortemporal_sa	0.289	0.065	*p<0.001*	0.376
Cognitive_factor	~	isthmuscingulate_sa	0.007	0.050	0.895	0.008
Cognitive_factor	~	lateraloccipital_sa	-0.097	0.066	0.144	-0.126
Cognitive_factor	~	lateralorbitofrontal_sa	-0.137	0.054	*0.012*	-0.180
Cognitive_factor	~	lingual_sa	0.115	0.058	*0.048*	0.152
Cognitive_factor	~	medialorbitofrontal_sa	0.221	0.049	*p<0.001*	0.283
Cognitive_factor	~	middletemporal_sa	-0.225	0.062	*p<0.001*	-0.298
Cognitive_factor	~	parahippocampal_sa	-0.062	0.038	0.106	-0.077
Cognitive_factor	~	paracentral_sa	0.072	0.053	0.176	0.090
Cognitive_factor	~	parsopercularis_sa	0.150	0.071	*0.034*	0.183
Cognitive_factor	~	parsorbitalis_sa	-0.020	0.045	0.652	-0.026
Cognitive_factor	~	parstriangularis_sa	-0.037	0.065	0.567	-0.046
Cognitive_factor	~	pericalcarine_sa	0.215	0.050	*p<0.001*	0.288
Cognitive_factor	~	postcentral_sa	-0.032	0.068	0.639	-0.041
Cognitive_factor	~	posteriorcingulate_sa	-0.112	0.062	0.072	-0.136
Cognitive_factor	~	precentral_sa	0.143	0.068	*0.035*	0.185
Cognitive_factor	~	precuneus_sa	-0.078	0.066	0.233	-0.104
Cognitive_factor	~	rostralanteriorcingulate_sa	-0.129	0.049	*0.008*	-0.158
Cognitive_factor	~	rostralmiddlefrontal_sa	0.307	0.065	*p<0.001*	0.401
Cognitive_factor	~	superiorfrontal_sa	-0.043	0.077	0.577	-0.057
Cognitive_factor	~	superiorparietal_sa	-0.027	0.067	0.685	-0.035
Cognitive_factor	~	superiortemporal_sa	-0.107	0.065	0.098	-0.141
Cognitive_factor	~	supramarginal_sa	0.028	0.072	0.694	0.036
Cognitive_factor	~	frontalpole_sa	0.031	0.027	0.263	0.037
Cognitive_factor	~	temporalpole_sa	-0.128	0.027	*p<0.001*	-0.159
Cognitive_factor	~	transversetemporal_sa	-0.155	0.034	*p<0.001*	-0.193
Cognitive_factor	~	insula_sa	-0.090	0.054	0.096	-0.117
Cognitive_factor	~	bankssts_gmv	0.028	0.047	0.551	0.033
Cognitive_factor	~	caudalanteriorcingulate_gmv	0.068	0.055	0.218	0.072
Cognitive_factor	~	caudalmiddlefrontal_gmv	-0.194	0.067	*0.004*	-0.240
Cognitive_factor	~	cuneus_gmv	0.016	0.057	0.775	0.021
Cognitive_factor	~	entorhinal_gmv	0.014	0.029	0.637	0.017
Cognitive_factor	~	fusiform_gmv	-0.047	0.054	0.383	-0.060
Cognitive_factor	~	inferiorparietal_gmv	-0.004	0.067	0.957	-0.005
Cognitive_factor	~	inferiortemporal_gmv	-0.187	0.062	*0.003*	-0.240
Cognitive_factor	~	isthmuscingulate_gmv	-0.057	0.049	0.246	-0.071

Cognitive_factor	~	lateraloccipital_gmv	0.116	0.071	0.102	0.151
Cognitive_factor	~	lateralorbitofrontal_gmv	0.158	0.051	*0.002*	0.210
Cognitive_factor	~	lingual_gmv	-0.131	0.065	*0.042*	-0.171
Cognitive_factor	~	medialorbitofrontal_gmv	-0.243	0.050	*p<0.001*	-0.306
Cognitive_factor	~	middletemporal_gmv	0.271	0.056	*p<0.001*	0.356
Cognitive_factor	~	parahippocampal_gmv	0.087	0.042	*0.038*	0.107
Cognitive_factor	~	paracentral_gmv	-0.093	0.054	0.084	-0.114
Cognitive_factor	~	parsopercularis_gmv	-0.117	0.067	0.080	-0.142
Cognitive_factor	~	parsorbitalis_gmv	0.032	0.042	0.445	0.040
Cognitive_factor	~	parstriangularis_gmv	0.004	0.062	0.945	0.005
Cognitive_factor	~	pericalcarine_gmv	-0.243	0.056	*p<0.001*	-0.324
Cognitive_factor	~	postcentral_gmv	-0.033	0.069	0.635	-0.042
Cognitive_factor	~	posteriorcingulate_gmv	0.084	0.060	0.162	0.100
Cognitive_factor	~	precentral_gmv	-0.095	0.066	0.150	-0.123
Cognitive_factor	~	precuneus_gmv	0.064	0.064	0.319	0.085
Cognitive_factor	~	rostralanteriorcingulate_gmv	0.119	0.048	*0.013*	0.140
Cognitive_factor	~	rostralmiddlefrontal_gmv	-0.290	0.063	*p<0.001*	-0.376
Cognitive_factor	~	superiorfrontal_gmv	0.081	0.068	0.234	0.106
Cognitive_factor	~	superiorparietal_gmv	0.062	0.067	0.355	0.080
Cognitive_factor	~	superiortemporal_gmv	0.155	0.061	*0.012*	0.201
Cognitive_factor	~	supramarginal_gmv	-0.046	0.068	0.493	-0.058
Cognitive_factor	~	frontalpole_gmv	-0.001	0.029	0.970	-0.001
Cognitive_factor	~	temporalpole_gmv	0.105	0.029	*p<0.001*	0.128
Cognitive_factor	~	transversetemporal_gmv	0.143	0.032	*p<0.001*	0.177
Cognitive_factor	~	insula_gmv	0.106	0.054	*0.048*	0.142

Supplementary Figure 11. P-value of the models estimating how the grey matter metrics of each region of interest together predict the cognitive factor

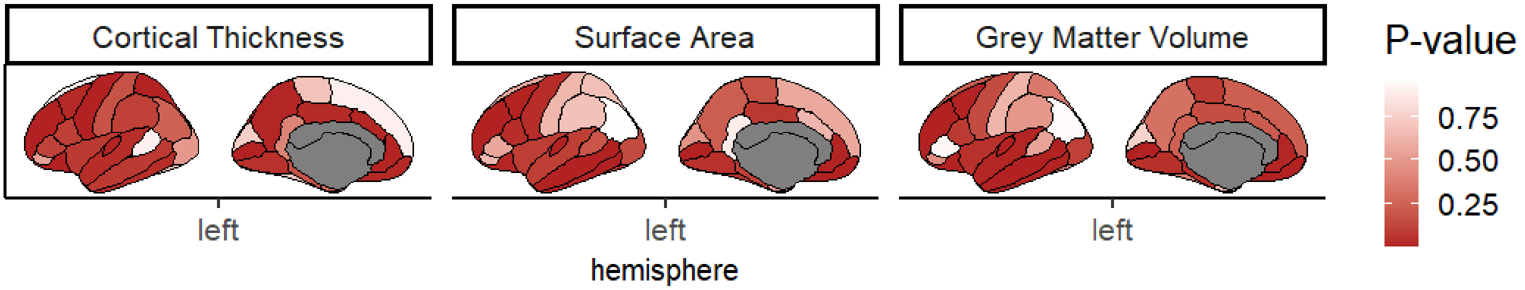

Supplementary Figure 12. Standardized parameter estimates of how the grey matter metrics of each region of interest together predict the cognitive factor

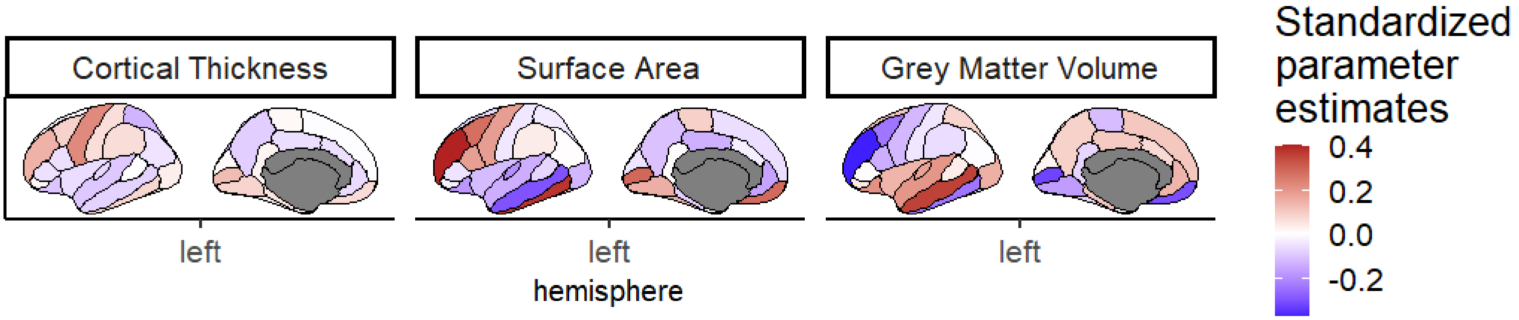

Supplementary Table 34. Comparison of the model with free parameters and the model with parameters constrained to a same value for the grey matter model with all the regions. AIC/BIC are two information criterions. Pr(>Chisq): p-value of the chi-square ratio test.

	Degree of freedom	AIC	BIC	Difference in degree of freedom	Pr (>Chisq)
**Free model**	413	129973.5	130817.1	NA	NA
**Constrained model**	514	130717.2	130832.5	101	*p<0.001*

Grey matter metrics (model with the regularized regions)

Supplementary Table 35. Regression estimates for the model estimating how cortical thickness, surface area and grey matter volume of the regularized regions predict the cognitive factor

Path			Estimate	SE	*p*	Standardized Estimate
Cognitive_factor	~	bankssts_ct	-0.017	0.013	0.182	-0.019
Cognitive_factor	~	caudalanteriorcingulate_ct	-0.053	0.011	*p<0.001*	-0.061
Cognitive_factor	~	cuneus_ct	0.001	0.012	0.944	0.001
Cognitive_factor	~	entorhinal_ct	0.016	0.010	0.119	0.019
Cognitive_factor	~	fusiform_ct	-0.008	0.014	0.574	-0.010
Cognitive_factor	~	isthmuscingulate_ct	-0.021	0.011	*0.050*	-0.025
Cognitive_factor	~	lateraloccipital_ct	0.061	0.014	*p<0.001*	0.082
Cognitive_factor	~	lateralorbitofrontal_ct	-0.019	0.013	0.140	-0.023
Cognitive_factor	~	medialorbitofrontal_ct	-0.052	0.012	*p<0.001*	-0.063
Cognitive_factor	~	middletemporal_ct	0.055	0.017	*0.001*	0.070
Cognitive_factor	~	paracentral_ct	-0.019	0.013	0.151	-0.024
Cognitive_factor	~	parstriangularis_ct	-0.055	0.013	*p<0.001*	-0.067
Cognitive_factor	~	rostralmiddlefrontal_ct	0.131	0.023	*p<0.001*	0.168
Cognitive_factor	~	superiorfrontal_ct	0.013	0.017	0.457	0.017
Cognitive_factor	~	supramarginal_ct	0.009	0.015	0.549	0.011
Cognitive_factor	~	frontalpole_ct	-0.024	0.014	0.082	-0.028
Cognitive_factor	~	transversetemporal_ct	0.014	0.011	0.205	0.017
Cognitive_factor	~	caudalanteriorcingulate_sa	0.041	0.017	*0.015*	0.046
Cognitive_factor	~	cuneus_sa	-0.012	0.016	0.463	-0.015
Cognitive_factor	~	fusiform_sa	0.068	0.015	*p<0.001*	0.089
Cognitive_factor	~	inferiortemporal_sa	0.088	0.019	*p<0.001*	0.115
Cognitive_factor	~	isthmus`ingulate_sa	-0.051	0.014	*p<0.001*	-0.065
Cognitive_factor	~	lingual_sa	0.010	0.016	0.517	0.014
Cognitive_factor	~	medialorbitofrontal_sa	-0.006	0.014	0.690	-0.007
Cognitive_factor	~	parahippocampal_sa	-0.150	0.017	*p<0.001*	-0.187
Cognitive_factor	~	pericalcarine_sa	0.010	0.018	0.572	0.014
Cognitive_factor	~	posteriorcingulate_sa	-0.044	0.016	*0.005*	-0.054
Cognitive_factor	~	precentral_sa	-0.064	0.028	*0.021*	-0.083
Cognitive_factor	~	rostralmiddlefrontal_sa	0.349	0.046	*p<0.001*	0.459
Cognitive_factor	~	superiorfrontal_sa	0.029	0.018	0.108	0.039
Cognitive_factor	~	superiorparietal_sa	0.029	0.012	*0.017*	0.038
Cognitive_factor	~	temporalpole_sa	-0.045	0.013	*0.001*	-0.056
Cognitive_factor	~	transversetemporal_sa	-0.015	0.012	0.212	-0.019
Cognitive_factor	~	insula_sa	0.001	0.023	0.979	0.001
Cognitive_factor	~	caudalmiddlefrontal_gmv	0.024	0.013	0.073	0.030
Cognitive_factor	~	middletemporal_gmv	0.053	0.017	*0.002*	0.070
Cognitive_factor	~	parahippocampal_gmv	0.176	0.015	*p<0.001*	0.217
Cognitive_factor	~	parsorbitalis_gmv	0.020	0.012	0.103	0.025

Cognitive_factor	~	precentral_gmv	0.117	0.026	*p<0.001*	0.152
Cognitive_factor	~	rostralanteriorcingulate_gmv	-0.011	0.015	0.463	-0.013
Cognitive_factor	~	rostralmiddlefrontal_gmv	-0.365	0.046	*p<0.001*	-0.475
Cognitive_factor	~	frontalpole_gmv	0.038	0.015	*0.012*	0.046
Cognitive_factor	~	insula_gmv	0.022	0.023	0.350	0.029

Supplementary Figure 13. P-value of the models estimating how the grey matter metrics of each regularized region of interest together predict the cognitive factor

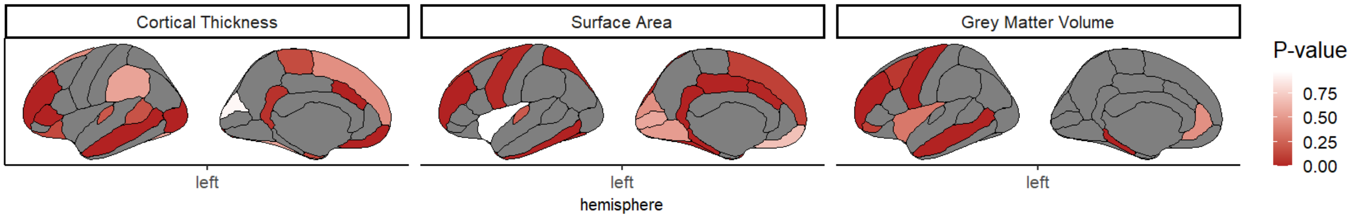

Supplementary Figure 14. Standardized parameter estimates of how the grey matter metrics of each regularized region of interest together predict the cognitive factor

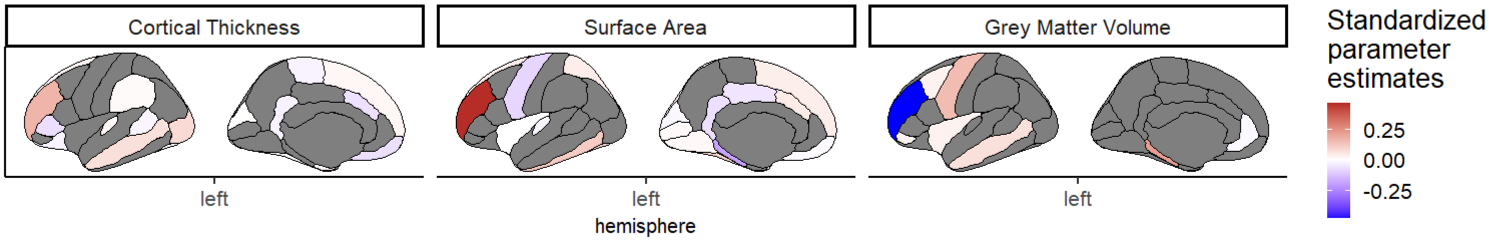

Supplementary Table 36. Comparison of the model with free parameters and the model with parameters constrained to a same value for the grey matter model with the regularized regions. AIC/BIC are two information criterions. Pr(>Chisq): p-value of the chi-square ratio test.

	**Degree of freedom**	**AIC**	**BIC**	**Difference in degree of freedom**	**Pr (>Chisq)**
**Free model**	177	130220.1	130638.2	NA	NA
**Constrained model**	219	130757.3	130872.6	42	*p<0.001*

White matter metrics (model with all the regions)

Supplementary Table 37. Regression estimates for the model estimating how fractional anisotropy, mean diffusivity and white matter volume of all the regions predict the cognitive factor

Path			Estimate	SE	*p*	Standardized Estimate
Cognitive_factor	~	fornix_fa	0.023	0.019	0.216	0.030
Cognitive_factor	~	cingulatecingulum_fa	-0.095	0.015	*p<0.001*	-0.121
Cognitive_factor	~	parahippocampalcingulum_fa	0.013	0.013	0.312	0.017
Cognitive_factor	~	corticospinalpyramidal_fa	0.061	0.019	*0.002*	0.080
Cognitive_factor	~	anteriorthalamicradiations_fa	0.038	0.017	*0.021*	0.050
Cognitive_factor	~	uncinate_fa	-0.071	0.020	*p<0.001*	-0.092
Cognitive_factor	~	inferiorlongitudinalfasiculus_fa	0.018	0.019	0.332	0.023
Cognitive_factor	~	inferiorfrontooccipitalfasiculus_fa	0.079	0.023	*0.001*	0.103
Cognitive_factor	~	forcepsmajor_fa	0.004	0.022	0.858	0.006
Cognitive_factor	~	forcepsminor_fa	-0.056	0.025	*0.023*	-0.078
Cognitive_factor	~	corpuscallosum_fa	-0.044	0.038	0.246	-0.061
Cognitive_factor	~	superiorlongitudinalfasiculus_fa	-0.171	0.143	0.230	-0.225
Cognitive_factor	~	temporalsuperiorlongitudinalfasiculus_fa	0.079	0.077	0.299	0.104
Cognitive_factor	~	parietalsuperiorlongitudinalfasiculus_fa	0.230	0.085	*0.007*	0.301
Cognitive_factor	~	superiorcorticostriate_fa	0.542	0.139	*p<0.001*	0.700
Cognitive_factor	~	superiorcorticostriatefrontalcortex_fa	-0.146	0.064	*0.023*	-0.189
Cognitive_factor	~	superiorcorticostriateparietalcortex_fa	-0.448	0.093	*p<0.001*	-0.573
Cognitive_factor	~	striatalinferiorfrontalcortex_fa	-0.054	0.018	*0.002*	-0.068
Cognitive_factor	~	inferiorfrontalsuperiorfrontalcortex_fa	0.064	0.022	*0.004*	0.083
Cognitive_factor	~	fornix_exfimbria_fa	-0.047	0.018	*0.009*	-0.059
Cognitive_factor	~	fornix_md	-0.005	0.026	0.844	-0.007
Cognitive_factor	~	cingulatecingulum_md	0.015	0.017	0.354	0.018
Cognitive_factor	~	parahippocampalcingulum_md	-0.012	0.015	0.445	-0.015
Cognitive_factor	~	corticospinalpyramidal_md	0.111	0.019	*p<0.001*	0.145
Cognitive_factor	~	anteriorthalamicradiations_md	0.065	0.017	*p<0.001*	0.085
Cognitive_factor	~	uncinate_md	-0.068	0.022	*0.002*	-0.089
Cognitive_factor	~	inferiorlongitudinalfasiculus_md	-0.004	0.023	0.852	-0.005
Cognitive_factor	~	inferiorfrontooccipitalfasiculus_md	0.065	0.027	*0.018*	0.081
Cognitive_factor	~	forcepsmajor_md	0.023	0.023	0.328	0.032
Cognitive_factor	~	forcepsminor_md	0.060	0.023	*0.008*	0.084
Cognitive_factor	~	corpuscallosum_md	-0.078	0.032	*0.013*	-0.107
Cognitive_factor	~	superiorlongitudinalfasiculus_md	-0.116	0.202	0.564	-0.151
Cognitive_factor	~	temporalsuperiorlongitudinalfasiculus_md	0.132	0.106	0.212	0.171
Cognitive_factor	~	parietalsuperiorlongitudinalfasiculus_md	-0.062	0.116	0.592	-0.080
Cognitive_factor	~	superiorcorticostriate_md	-0.009	0.164	0.957	-0.011
Cognitive_factor	~	superiorcorticostriatefrontalcortex_md	-0.040	0.074	0.583	-0.051
Cognitive_factor	~	superiorcorticostriateparietalcortex_md	-0.106	0.114	0.353	-0.134
Cognitive_factor	~	striatalinferiorfrontalcortex_md	-0.061	0.027	*0.023*	-0.074
Cognitive_factor	~	inferiorfrontalsuperiorfrontalcortex_md	0.101	0.032	*0.002*	0.131
Cognitive_factor	~	fornix_exfimbria_md	0.022	0.025	0.376	0.029
Cognitive_factor	~	fornix_wmv	0.237	0.032	*p<0.001*	0.322

Cognitive_factor	~	cingulatecingulum_wmv	-0.021	0.019	0.271	-0.028
Cognitive_factor	~	parahippocampalcingulum_wmv	0.039	0.013	*0.004*	0.051
Cognitive_factor	~	corticospinalpyramidal_wmv	0.198	0.024	*p<0.001*	0.266
Cognitive_factor	~	anteriorthalamicradiations_wmv	0.018	0.027	0.493	0.025
Cognitive_factor	~	uncinate_wmv	-0.007	0.018	0.698	-0.009
Cognitive_factor	~	inferiorlongitudinalfasiculus_wmv	-0.003	0.021	0.873	-0.004
Cognitive_factor	~	inferiorfrontooccipitalfasiculus_wmv	-0.054	0.032	0.089	-0.073
Cognitive_factor	~	forcepsmajor_wmv	0.000	0.021	0.996	0.000
Cognitive_factor	~	forcepsminor_wmv	-0.046	0.026	0.084	-0.064
Cognitive_factor	~	corpuscallosum_wmv	0.082	0.044	0.060	0.114
Cognitive_factor	~	superiorlongitudinalfasiculus_wmv	-0.111	0.084	0.187	-0.147
Cognitive_factor	~	temporalsuperiorlongitudinalfasiculus_wmv	0.147	0.056	*0.009*	0.193
Cognitive_factor	~	parietalsuperiorlongitudinalfasiculus_wmv	0.009	0.047	0.849	0.012
Cognitive_factor	~	superiorcorticostriate_wmv	-0.085	0.087	0.331	-0.114
Cognitive_factor	~	superiorcorticostriatefrontalcortex_wmv	-0.158	0.053	*0.003*	-0.212
Cognitive_factor	~	superiorcorticostriateparietalcortex_wmv	-0.008	0.057	0.885	-0.011
Cognitive_factor	~	striatalinferiorfrontalcortex_wmv	0.030	0.021	0.162	0.039
Cognitive_factor	~	inferiorfrontalsuperiorfrontalcortex_wmv	0.076	0.024	*0.002*	0.101
Cognitive_factor	~	fornix_exfimbria_wmv	-0.065	0.031	*0.036*	-0.084

Supplementary Table 38. Comparison of the model with free parameters and the model with parameters constrained to a same value for the white matter model with all the regions. AIC/BIC are two information criterions. Pr(>Chisq): p-value of the chi-square ratio test.

	**Degree of freedom**	**AIC**	**BIC**	**Difference in degree of freedom**	**Pr (>Chisq)**
Free model	245	122708.0	123244.7	NA	NA
Constrained model	304	123602.8	123717.3	59	*p<0.001*

White matter metrics (model with the regularized regions)

Supplementary Table 39. Regression estimates for the model estimating how fractional anisotropy, mean diffusivity and white matter volume of the regularized regions predict the cognitive factor

Path			Estimate	SE	*p*	Standardized Estimate
Cognitive_factor	~	fornix_fa	0.026	0.012	*0.028*	0.035
Cognitive_factor	~	cingulatecingulum_fa	-0.119	0.012	*p<0.001*	-0.155
Cognitive_factor	~	anteriorthalamicradiations_fa	0.009	0.014	0.536	0.012
Cognitive_factor	~	inferiorlongitudinalfasiculus_fa	0.003	0.015	0.850	0.004
Cognitive_factor	~	superiorlongitudinalfasiculus_fa	0.135	0.015	*p<0.001*	0.182
Cognitive_factor	~	striatalinferiorfrontalcortex_fa	-0.065	0.013	*p<0.001*	-0.086
Cognitive_factor	~	inferiorfrontalsuperiorfrontalcortex_fa	0.049	0.016	*0.002*	0.066
Cognitive_factor	~	anteriorthalamicradiations_md	0.068	0.016	*p<0.001*	0.094
Cognitive_factor	~	inferiorlongitudinalfasiculus_md	-0.021	0.021	0.325	-0.028
Cognitive_factor	~	inferiorfrontooccipitalfasiculus_md	0.013	0.024	0.589	0.017
Cognitive_factor	~	forcepsmajor_md	0.021	0.014	0.135	0.029
Cognitive_factor	~	superiorcorticostriateparietalcortex_md	-0.063	0.017	*p<0.001*	-0.086
Cognitive_factor	~	fornix_exfimbria_md	-0.016	0.012	0.184	-0.021
Cognitive_factor	~	corticospinalpyramidal_wmv	0.144	0.018	*p<0.001*	0.197
Cognitive_factor	~	anteriorthalamicradiations_wmv	-0.008	0.023	0.721	-0.012
Cognitive_factor	~	uncinate_wmv	-0.017	0.016	0.296	-0.023
Cognitive_factor	~	inferiorlongitudinalfasiculus_wmv	0.010	0.019	0.595	0.013
Cognitive_factor	~	inferiorfrontooccipitalfasiculus_wmv	-0.002	0.028	0.942	-0.003
Cognitive_factor	~	forcepsmajor_wmv	0.049	0.019	*0.008*	0.069
Cognitive_factor	~	forcepsminor_wmv	-0.035	0.023	0.128	-0.050
Cognitive_factor	~	corpuscallosum_wmv	0.042	0.032	0.190	0.059
Cognitive_factor	~	temporalsuperiorlongitudinalfasiculus_wmv	0.073	0.017	*p<0.001*	0.097
Cognitive_factor	~	striatalinferiorfrontalcortex_wmv	-0.005	0.019	0.788	-0.007

Supplementary Table 40. Standardized parameter estimates of how the white matter metrics of each regularized region of interest together predict the cognitive factor

Path			FA	MD	WMV
Cognitive_factor	~	corpuscallosum	-	-	0.059
Cognitive_factor	~	forcepsmajor	-	0.029	0.069
Cognitive_factor	~	forcepsminor	-	-	-0.050
Cognitive_factor	~	anteriorthalamicradiations	0.012	0.094	-0.012
Cognitive_factor	~	cingulatecingulum	-0.155	-	-
Cognitive_factor	~	corticospinalpyramidal	-	-	0.197
Cognitive_factor	~	fornix	0.035	-	-
Cognitive_factor	~	fornix_exfimbria	-	-0.021	-
Cognitive_factor	~	inferiorfrontalsuperiorfrontalcortex	0.066	-	-
Cognitive_factor	~	inferiorfrontooccipitalfasiculus	-	0.017	-0.003
Cognitive_factor	~	inferiorlongitudinalfasiculus	0.004	-0.028	0.013
Cognitive_factor	~	striatalinferiorfrontalcortex	-0.086	-	-0.007
Cognitive_factor	~	superiorcorticostriateparietalcortex	-	-0.09	-
Cognitive_factor	~	superiorlongitudinalfasiculus	0.182	-	-
Cognitive_factor	~	temporalsuperiorlongitudinalfasiculus	-	-	0.097
Cognitive_factor	~	uncinate	-	-	-0.023

Supplementary Table 41. Comparison of the model with free parameters and the model with parameters constrained to a same value for the white matter model with the regularized regions. AIC/BIC are two information criterions. Pr(>Chisq): p-value of the chi-square ratio test.

	Degree of freedom	AIC	BIC	Difference in degree of freedom	Pr (>Chisq)
Free model	97	123117.4	123389.4	NA	NA
Constrained model	119	123601.0	123715.5	22	*p<0.001*

Models with grey and white matter metrics estimating cognitive factor from several regions in the six metrics

For the grey and white matter model, we reported the table for the model with all the regions and the model with only the regions that survived the regularization included as predictors (the one that we used for the analyses).

Grey and white matter metrics (model with all the regions)

Supplementary Table 42. Regression estimates for the model estimating how cortical thickness, surface area, grey matter volume, fractional anisotropy, mean diffusivity and white matter volume of all the regions predict the cognitive factor

Path			Estimate	SE	*p*	Standardized Estimate
Cognitive_factor	~	bankssts_ct	0.002	0.018	0.916	0.002
Cognitive_factor	~	caudalanteriorcingulate_ct	-0.027	0.018	0.136	-0.032
Cognitive_factor	~	caudalmiddlefrontal_ct	0.015	0.030	0.622	0.018
Cognitive_factor	~	cuneus_ct	0.007	0.031	0.812	0.009
Cognitive_factor	~	entorhinal_ct	0.014	0.020	0.485	0.016
Cognitive_factor	~	fusiform_ct	-0.003	0.026	0.920	-0.003
Cognitive_factor	~	inferiorparietal_ct	-0.022	0.032	0.486	-0.028
Cognitive_factor	~	inferiortemporal_ct	0.042	0.030	0.152	0.052
Cognitive_factor	~	isthmuscingulate_ct	0.022	0.020	0.283	0.026
Cognitive_factor	~	lateraloccipital_ct	-0.007	0.038	0.845	-0.010
Cognitive_factor	~	lateralorbitofrontal_ct	-0.076	0.023	*0.001*	-0.092
Cognitive_factor	~	lingual_ct	0.061	0.032	0.055	0.078
Cognitive_factor	~	medialorbitofrontal_ct	0.040	0.024	0.097	0.047
Cognitive_factor	~	middletemporal_ct	-0.033	0.034	0.335	-0.041
Cognitive_factor	~	parahippocampal_ct	0.028	0.029	0.331	0.035
Cognitive_factor	~	paracentral_ct	0.015	0.028	0.581	0.019
Cognitive_factor	~	parsopercularis_ct	-0.038	0.027	0.163	-0.045
Cognitive_factor	~	parsorbitalis_ct	0.006	0.024	0.806	0.007
Cognitive_factor	~	parstriangularis_ct	-0.050	0.027	0.068	-0.059
Cognitive_factor	~	pericalcarine_ct	0.049	0.030	0.096	0.063
Cognitive_factor	~	postcentral_ct	0.013	0.037	0.735	0.016
Cognitive_factor	~	posteriorcingulate_ct	-0.029	0.023	0.206	-0.034
Cognitive_factor	~	precentral_ct	0.166	0.039	*p<0.001*	0.208
Cognitive_factor	~	precuneus_ct	-0.053	0.026	*0.045*	-0.067
Cognitive_factor	~	rostralanteriorcingulate_ct	-0.038	0.020	0.059	-0.043
Cognitive_factor	~	rostralmiddlefrontal_ct	0.115	0.031	*p<0.001*	0.144
Cognitive_factor	~	superiorfrontal_ct	-0.007	0.033	0.827	-0.009
Cognitive_factor	~	superiorparietal_ct	-0.061	0.036	0.084	-0.080
Cognitive_factor	~	superiortemporal_ct	-0.072	0.036	*0.043*	-0.091
Cognitive_factor	~	supramarginal_ct	0.052	0.032	0.101	0.065
Cognitive_factor	~	frontalpole_ct	0.004	0.022	0.868	0.004

Cognitive_factor	~	temporalpole_ct	-0.038	0.022	0.077	-0.045
Cognitive_factor	~	transversetemporal_ct	-0.043	0.020	*0.027*	-0.052
Cognitive_factor	~	insula_ct	-0.023	0.028	0.399	-0.028
Cognitive_factor	~	bankssts_sa	-0.059	0.046	0.202	-0.070
Cognitive_factor	~	caudalanteriorcingulate_sa	0.018	0.048	0.703	0.020
Cognitive_factor	~	caudalmiddlefrontal_sa	0.069	0.073	0.343	0.086
Cognitive_factor	~	cuneus_sa	-0.035	0.049	0.472	-0.046
Cognitive_factor	~	entorhinal_sa	0.009	0.033	0.784	0.011
Cognitive_factor	~	fusiform_sa	0.061	0.057	0.289	0.079
Cognitive_factor	~	inferiorparietal_sa	0.056	0.070	0.421	0.072
Cognitive_factor	~	inferiortemporal_sa	0.231	0.065	*p<0.001*	0.299
Cognitive_factor	~	isthmuscingulate_sa	-0.001	0.053	0.978	-0.002
Cognitive_factor	~	lateraloccipital_sa	-0.143	0.068	*0.034*	-0.186
Cognitive_factor	~	lateralorbitofrontal_sa	-0.108	0.055	*0.048*	-0.142
Cognitive_factor	~	lingual_sa	0.113	0.060	0.057	0.149
Cognitive_factor	~	medialorbitofrontal_sa	0.169	0.051	*0.001*	0.215
Cognitive_factor	~	middletemporal_sa	-0.168	0.063	*0.008*	-0.222
Cognitive_factor	~	parahippocampal_sa	-0.060	0.039	0.129	-0.074
Cognitive_factor	~	paracentral_sa	0.070	0.055	0.205	0.086
Cognitive_factor	~	parsopercularis_sa	0.151	0.071	*0.032*	0.184
Cognitive_factor	~	parsorbitalis_sa	-0.019	0.046	0.683	-0.024
Cognitive_factor	~	parstriangularis_sa	-0.084	0.067	0.212	-0.103
Cognitive_factor	~	pericalcarine_sa	0.123	0.052	*0.018*	0.164
Cognitive_factor	~	postcentral_sa	-0.042	0.070	0.551	-0.054
Cognitive_factor	~	posteriorcingulate_sa	-0.097	0.073	0.184	-0.116
Cognitive_factor	~	precentral_sa	0.149	0.070	*0.032*	0.192
Cognitive_factor	~	precuneus_sa	-0.023	0.066	0.727	-0.031
Cognitive_factor	~	rostralanteriorcingulate_sa	-0.118	0.050	*0.018*	-0.144
Cognitive_factor	~	rostralmiddlefrontal_sa	0.261	0.068	*p<0.001*	0.339
Cognitive_factor	~	superiorfrontal_sa	-0.007	0.078	0.932	-0.009
Cognitive_factor	~	superiorparietal_sa	0.012	0.069	0.862	0.016
Cognitive_factor	~	superiortemporal_sa	-0.095	0.066	0.150	-0.124
Cognitive_factor	~	supramarginal_sa	0.050	0.074	0.497	0.063
Cognitive_factor	~	frontalpole_sa	0.022	0.028	0.432	0.026
Cognitive_factor	~	temporalpole_sa	-0.120	0.027	*p<0.001*	-0.148
Cognitive_factor	~	transversetemporal_sa	-0.122	0.034	*p<0.001*	-0.151
Cognitive_factor	~	insula_sa	-0.057	0.056	0.303	-0.074
Cognitive_factor	~	bankssts_gmv	0.033	0.047	0.485	0.039
Cognitive_factor	~	caudalanteriorcingulate_gmv	0.014	0.050	0.785	0.014
Cognitive_factor	~	caudalmiddlefrontal_gmv	-0.041	0.070	0.561	-0.050
Cognitive_factor	~	cuneus_gmv	0.021	0.058	0.721	0.027
Cognitive_factor	~	entorhinal_gmv	0.009	0.030	0.755	0.012
Cognitive_factor	~	fusiform_gmv	-0.030	0.056	0.590	-0.038
Cognitive_factor	~	inferiorparietal_gmv	-0.067	0.068	0.321	-0.085

Cognitive_factor	~	inferiortemporal_gmv	-0.160	0.063	*0.011*	-0.204
Cognitive_factor	~	isthmuscingulate_gmv	-0.055	0.052	0.290	-0.068
Cognitive_factor	~	lateraloccipital_gmv	0.161	0.072	*0.026*	0.209
Cognitive_factor	~	lateralorbitofrontal_gmv	0.150	0.052	*0.004*	0.198
Cognitive_factor	~	lingual_gmv	-0.135	0.066	*0.041*	-0.175
Cognitive_factor	~	medialorbitofrontal_gmv	-0.161	0.052	*0.002*	-0.201
Cognitive_factor	~	middletemporal_gmv	0.196	0.057	*0.001*	0.257
Cognitive_factor	~	parahippocampal_gmv	0.058	0.043	0.176	0.071
Cognitive_factor	~	paracentral_gmv	-0.077	0.055	0.164	-0.093
Cognitive_factor	~	parsopercularis_gmv	-0.114	0.067	0.090	-0.137
Cognitive_factor	~	parsorbitalis_gmv	0.038	0.043	0.372	0.047
Cognitive_factor	~	parstriangularis_gmv	0.048	0.064	0.449	0.058
Cognitive_factor	~	pericalcarine_gmv	-0.150	0.058	*0.010*	-0.199
Cognitive_factor	~	postcentral_gmv	-0.017	0.071	0.809	-0.022
Cognitive_factor	~	posteriorcingulate_gmv	0.065	0.069	0.351	0.077
Cognitive_factor	~	precentral_gmv	-0.109	0.068	0.107	-0.140
Cognitive_factor	~	precuneus_gmv	0.014	0.065	0.830	0.018
Cognitive_factor	~	rostralanteriorcingulate_gmv	0.119	0.048	*0.014*	0.139
Cognitive_factor	~	rostralmiddlefrontal_gmv	-0.245	0.065	*p<0.001*	-0.315
Cognitive_factor	~	superiorfrontal_gmv	0.056	0.069	0.418	0.073
Cognitive_factor	~	superiorparietal_gmv	0.011	0.069	0.873	0.014
Cognitive_factor	~	superiortemporal_gmv	0.147	0.063	*0.019*	0.190
Cognitive_factor	~	supramarginal_gmv	-0.070	0.069	0.311	-0.087
Cognitive_factor	~	frontalpole_gmv	-0.007	0.029	0.820	-0.008
Cognitive_factor	~	temporalpole_gmv	0.097	0.030	*0.001*	0.116
Cognitive_factor	~	transversetemporal_gmv	0.110	0.033	*0.001*	0.136
Cognitive_factor	~	insula_gmv	0.084	0.055	0.127	0.111
Cognitive_factor	~	fornix_fa	0.019	0.019	0.332	0.024
Cognitive_factor	~	cingulatecingulum_fa	-0.044	0.016	*0.006*	-0.056
Cognitive_factor	~	parahippocampalcingulum_fa	-0.001	0.013	0.955	-0.001
Cognitive_factor	~	corticospinalpyramidal_fa	0.048	0.020	*0.015*	0.062
Cognitive_factor	~	anteriorthalamicradiations_fa	0.034	0.017	*0.048*	0.043
Cognitive_factor	~	uncinate_fa	-0.056	0.020	*0.005*	-0.071
Cognitive_factor	~	inferiorlongitudinalfasiculus_fa	0.015	0.019	0.413	0.019
Cognitive_factor	~	inferiorfrontooccipitalfasiculus_fa	0.071	0.024	*0.003*	0.092
Cognitive_factor	~	forcepsmajor_fa	0.020	0.023	0.375	0.028
Cognitive_factor	~	forcepsminor_fa	-0.026	0.024	0.289	-0.036
Cognitive_factor	~	corpuscallosum_fa	-0.086	0.040	*0.030*	-0.118
Cognitive_factor	~	superiorlongitudinalfasiculus_fa	-0.076	0.142	0.594	-0.099
Cognitive_factor	~	temporalsuperiorlongitudinalfasiculus_fa	0.040	0.077	0.602	0.052
Cognitive_factor	~	parietalsuperiorlongitudinalfasiculus_fa	0.124	0.084	0.142	0.161
Cognitive_factor	~	superiorcorticostriate_fa	0.365	0.132	*0.006*	0.469
Cognitive_factor	~	superiorcorticostriatefrontalcortex_fa	-0.088	0.059	0.132	-0.114
Cognitive_factor	~	superiorcorticostriateparietalcortex_fa	-0.320	0.091	*p<0.001*	-0.407

Cognitive_factor	~	striatalinferiorfrontalcortex_fa	-0.047	0.018	*0.008*	-0.059
Cognitive_factor	~	inferiorfrontalsuperiorfrontalcortex_fa	0.055	0.022	*0.013*	0.071
Cognitive_factor	~	fornix_exfimbria_fa	-0.050	0.018	*0.005*	-0.062
Cognitive_factor	~	fornix_md	-0.016	0.024	0.497	-0.021
Cognitive_factor	~	cingulatecingulum_md	0.007	0.016	0.654	0.008
Cognitive_factor	~	parahippocampalcingulum_md	-0.026	0.015	0.085	-0.034
Cognitive_factor	~	corticospinalpyramidal_md	0.096	0.019	*p<0.001*	0.124
Cognitive_factor	~	anteriorthalamicradiations_md	0.041	0.017	*0.016*	0.054
Cognitive_factor	~	uncinate_md	-0.042	0.021	*0.043*	-0.055
Cognitive_factor	~	inferiorlongitudinalfasiculus_md	-0.006	0.023	0.801	-0.007
Cognitive_factor	~	inferiorfrontooccipitalfasiculus_md	0.031	0.027	0.247	0.039
Cognitive_factor	~	forcepsmajor_md	0.060	0.021	*0.004*	0.083
Cognitive_factor	~	forcepsminor_md	0.067	0.023	*0.003*	0.093
Cognitive_factor	~	corpuscallosum_md	-0.120	0.032	*p<0.001*	-0.163
Cognitive_factor	~	superiorlongitudinalfasiculus_md	-0.166	0.204	0.415	-0.214
Cognitive_factor	~	temporalsuperiorlongitudinalfasiculus_md	0.149	0.107	0.163	0.192
Cognitive_factor	~	parietalsuperiorlongitudinalfasiculus_md	-0.023	0.116	0.844	-0.029
Cognitive_factor	~	superiorcorticostriate_md	0.119	0.160	0.457	0.149
Cognitive_factor	~	superiorcorticostriatefrontalcortex_md	-0.147	0.073	*0.044*	-0.185
Cognitive_factor	~	superiorcorticostriateparietalcortex_md	-0.123	0.111	0.266	-0.155
Cognitive_factor	~	striatalinferiorfrontalcortex_md	-0.035	0.026	0.171	-0.043
Cognitive_factor	~	inferiorfrontalsuperiorfrontalcortex_md	0.134	0.032	*p<0.001*	0.172
Cognitive_factor	~	fornix_exfimbria_md	0.022	0.024	0.365	0.029
Cognitive_factor	~	fornix_wmv	0.134	0.034	*p<0.001*	0.182
Cognitive_factor	~	cingulatecingulum_wmv	-0.006	0.021	0.776	-0.008
Cognitive_factor	~	parahippocampalcingulum_wmv	0.041	0.014	*0.004*	0.053
Cognitive_factor	~	corticospinalpyramidal_wmv	0.194	0.024	*p<0.001*	0.260
Cognitive_factor	~	anteriorthalamicradiations_wmv	0.028	0.028	0.306	0.038
Cognitive_factor	~	uncinate_wmv	-0.001	0.019	0.972	-0.001
Cognitive_factor	~	inferiorlongitudinalfasiculus_wmv	-0.010	0.022	0.662	-0.013
Cognitive_factor	~	inferiorfrontooccipitalfasiculus_wmv	-0.073	0.032	*0.025*	-0.097
Cognitive_factor	~	forcepsmajor_wmv	0.011	0.022	0.601	0.016
Cognitive_factor	~	forcepsminor_wmv	-0.108	0.028	*p<0.001*	-0.150
Cognitive_factor	~	corpuscallosum_wmv	0.094	0.046	*0.038*	0.131
Cognitive_factor	~	superiorlongitudinalfasiculus_wmv	-0.110	0.084	0.188	-0.146
Cognitive_factor	~	temporalsuperiorlongitudinalfasiculus_wmv	0.125	0.056	*0.025*	0.164
Cognitive_factor	~	parietalsuperiorlongitudinalfasiculus_wmv	0.026	0.048	0.588	0.034
Cognitive_factor	~	superiorcorticostriate_wmv	0.052	0.087	0.551	0.069
Cognitive_factor	~	superiorcorticostriatefrontalcortex_wmv	-0.219	0.053	*p<0.001*	-0.292
Cognitive_factor	~	superiorcorticostriateparietalcortex_wmv	-0.070	0.057	0.218	-0.092
Cognitive_factor	~	striatalinferiorfrontalcortex_wmv	0.006	0.022	0.804	0.007
Cognitive_factor	~	inferiorfrontalsuperiorfrontalcortex_wmv	0.005	0.026	0.851	0.007
Cognitive_factor	~	fornix_exfimbria_wmv	-0.005	0.032	0.880	-0.006

Supplementary Table 43. Comparison of the model with free parameters and the model with parameters constrained to a same value for the grey & white matter model with all the regions. AIC/BIC are two information criterions. Pr(>Chisq): p-value of the chi-square ratio test.

	**Degree of freedom**	**AIC**	**BIC**	**Difference in degree of freedom**	**Pr (>Chisq)**
Free model	653	122276	123543	NA	NA
Constrained model	814	123378	123492	161	*p<0.001*

Grey and white matter metrics (model with the regularized regions)

Supplementary Table 44. Regression estimates for the model estimating how cortical thickness, surface area, grey matter volume, fractional anisotropy, mean diffusivity and white matter volume of the regularized regions predict the cognitive factor

**Path**			**Estimate**	**SE**	***p***	**Standardized Estimate**
Cognitive_factor	~	caudalanteriorcingulate_ct	-0.032	0.012	*0.008*	-0.037
Cognitive_factor	~	entorhinal_ct	0.013	0.010	0.208	0.016
Cognitive_factor	~	lateraloccipital_ct	0.067	0.015	*p<0.001*	0.089
Cognitive_factor	~	lingual_ct	0.001	0.011	0.911	0.002
Cognitive_factor	~	medialorbitofrontal_ct	-0.031	0.012	*0.008*	-0.038
Cognitive_factor	~	middletemporal_ct	0.013	0.014	0.365	0.016
Cognitive_factor	~	parahippocampal_ct	0.094	0.012	*p<0.001*	0.120
Cognitive_factor	~	parsopercularis_ct	-0.077	0.014	*p<0.001*	-0.092
Cognitive_factor	~	parstriangularis_ct	-0.030	0.015	*0.047*	-0.036
Cognitive_factor	~	precentral_ct	0.086	0.015	*p<0.001*	0.110
Cognitive_factor	~	precuneus_ct	-0.033	0.015	*0.024*	-0.043
Cognitive_factor	~	rostralanteriorcingulate_ct	-0.018	0.013	0.163	-0.021
Cognitive_factor	~	rostralmiddlefrontal_ct	0.019	0.014	0.186	0.024
Cognitive_factor	~	superiorparietal_ct	-0.065	0.016	*p<0.001*	-0.085
Cognitive_factor	~	transversetemporal_ct	0.026	0.011	*0.022*	0.031
Cognitive_factor	~	insula_ct	-0.007	0.012	0.558	-0.009
Cognitive_factor	~	caudalmiddlefrontal_sa	0.037	0.014	*0.009*	0.047
Cognitive_factor	~	cuneus_sa	-0.011	0.016	0.513	-0.014
Cognitive_factor	~	isthmuscingulate_sa	-0.067	0.014	*p<0.001*	-0.085
Cognitive_factor	~	parsopercularis_sa	0.042	0.014	*0.002*	0.051
Cognitive_factor	~	pericalcarine_sa	0.006	0.015	0.691	0.008
Cognitive_factor	~	posteriorcingulate_sa	0.049	0.052	0.348	0.059
Cognitive_factor	~	superiorparietal_sa	0.014	0.012	0.263	0.018
Cognitive_factor	~	entorhinal_gmv	0.023	0.011	*0.040*	0.029

Cognitive_factor	~	middletemporal_gmv	0.049	0.013	*p<0.001*	0.064
Cognitive_factor	~	parahippocampal_gmv	0.099	0.014	*p<0.001*	0.132
Cognitive_factor	~	parstriangularis_gmv	0.003	0.013	0.842	0.003
Cognitive_factor	~	posteriorcingulate_gmv	-0.007	0.013	0.597	-0.009
Cognitive_factor	~	precentral_gmv	-0.076	0.046	0.097	-0.092
Cognitive_factor	~	rostralanteriorcingulate_gmv	0.016	0.015	0.287	0.021
Cognitive_factor	~	superiortemporal_gmv	0.040	0.015	*0.007*	0.047
Cognitive_factor	~	fornix_fa	0.011	0.014	0.408	0.015
Cognitive_factor	~	cingulatecingulum_fa	0.003	0.012	0.783	0.004
Cognitive_factor	~	anteriorthalamicradiations_fa	-0.065	0.013	*p<0.001*	-0.084
Cognitive_factor	~	inferiorlongitudinalfasiculus_fa	-0.017	0.013	0.198	-0.023
Cognitive_factor	~	superiorlongitudinalfasiculus_fa	-0.002	0.014	0.883	-0.003
Cognitive_factor	~	superiorcorticostriatefrontalcortex_fa	0.083	0.015	*p<0.001*	0.112
Cognitive_factor	~	inferiorfrontalsuperiorfrontalcortex_fa	0.024	0.014	0.093	0.032
Cognitive_factor	~	cingulatecingulum_md	0.003	0.017	0.848	0.004
Cognitive_factor	~	uncinate_md	0.014	0.015	0.361	0.016
Cognitive_factor	~	inferiorfrontooccipitalfasiculus_md	0.015	0.019	0.445	0.020
Cognitive_factor	~	superiorlongitudinalfasiculus_md	0.038	0.019	*0.046*	0.051
Cognitive_factor	~	superiorcorticostriateparietalcortex_md	-0.030	0.022	0.168	-0.041
Cognitive_factor	~	corticospinalpyramidal_wmv	-0.042	0.020	*0.033*	-0.057
Cognitive_factor	~	uncinate_wmv	0.233	0.023	*p<0.001*	0.318
Cognitive_factor	~	forcepsminor_wmv	-0.023	0.015	0.134	-0.031
Cognitive_factor	~	corpuscallosum_wmv	-0.111	0.021	*p<0.001*	-0.157
Cognitive_factor	~	superiorcorticostriatefrontalcortex_wmv	0.162	0.025	*p<0.001*	0.229
Cognitive_factor	~	striatalinferiorfrontalcortex_wmv	-0.204	0.028	*p<0.001*	-0.278

Supplementary Table 45. Standardized parameter estimates of how the grey & white matter metrics of each regularized region of interest together predict the cognitive factor

	Path		CT	SA	GMV	FA	MD	WMV
Cognitive_factor	~	caudalanteriorcingulate	-0.037	-	-			
Cognitive_factor	~	caudalmiddlefrontal	-	0.047	-			
Cognitive_factor	~	cuneus	-	-0.014	-			
Cognitive_factor	~	entorhinal	0.016	-	0.029			
Cognitive_factor	~	fusiform	-	-	0.064			
Cognitive_factor	~	insula	-0.009	-	-			
Cognitive_factor	~	isthmuscingulate	-	-0.085	-			
Cognitive_factor	~	lateraloccipital	0.089	-	-			
Cognitive_factor	~	lingual	0.002	-	-			
Cognitive_factor	~	medialorbitofrontal	-0.038	-	-			
Cognitive_factor	~	middletemporal	0.016	-	0.132			
Cognitive_factor	~	parahippocampal	0.120	-	0.003			
Cognitive_factor	~	parsopercularis	-0.092	0.051	-			

Cognitive_factor	~	parstriangularis	-0.036	-	-0.009			
Cognitive_factor	~	pericalcarine	-	0.008	-			
Cognitive_factor	~	posteriorcingulate	-	0.059	-0.092			
Cognitive_factor	~	precentral	0.110	-	0.021			
Cognitive_factor	~	precuneus	-0.043	-	-			
Cognitive_factor	~	rostralanteriorcingulate	-0.021	-	0.047			
Cognitive_factor	~	rostralmiddlefrontal	0.024	-	-			
Cognitive_factor	~	superiorparietal	-0.085	0.018	-			
Cognitive_factor	~	superiortemporal	-	-	0.015			
Cognitive_factor	~	transversetemporal	0.031	-	-			
Cognitive_factor	~	corpuscallosum				-	-	0.229
Cognitive_factor	~	forcepsminor				-	-	-0.157
Cognitive_factor	~	anteriorthalamicradiations				-0.023	-	-
Cognitive_factor	~	cingulatecingulum				-0.084	0.016	-
Cognitive_factor	~	corticospinalpyramidal				-	-	0.318
Cognitive_factor	~	fornix				0.004	-	-
Cognitive_factor	~	inferiorfrontalsuperiorfrontalcortex				0.004	-	-
Cognitive_factor	~	inferiorfrontooccipitalfasiculus				-	0.051	-
Cognitive_factor	~	inferiorlongitudinalfasiculus				-0.003	-	-
Cognitive_factor	~	striatalinferiorfrontalcortex				-	-	0.008
Cognitive_factor	~	superiorcorticostriatefrontalcortex				0.032	-	-0.278
Cognitive_factor	~	superiorcorticostriateparietalcortex				-	-0.057	-
Cognitive_factor	~	superiorlongitudinalfasiculus				0.112	-0.041	-
Cognitive_factor	~	superiorlongitudinalfasiculus				-	0.020	-0.031
Cognitive_factor	~	uncinate				-	-	0.229

Supplementary Table 46. Comparison of the model with free parameters and the model with parameters constrained to a same value for the grey & white matter model with the regularized regions. AIC/BIC are two information criterions. Pr(>Chisq): p-value of the chi-square ratio test.

	Degree of freedom	AIC	BIC	Difference in degree of freedom	Pr (>Chisq)
Free model	205	122675.3	123140.5	NA	NA
Constrained model	254	123490.5	123605.0	49	*p<0.001*

Comparison between models with grey & white matter metrics and with grey & white matter metrics & TIV

We compare the parameter estimates of each region/tract between a model with grey and white matter metrics and a model with grey and white matter metrics and TIV.

Supplementary Figure 15. Standardized parameter estimates comparison of how the grey matter metrics (top) and the white matter metrics (bottom) predict the cognitive factor in a model with and without TIV

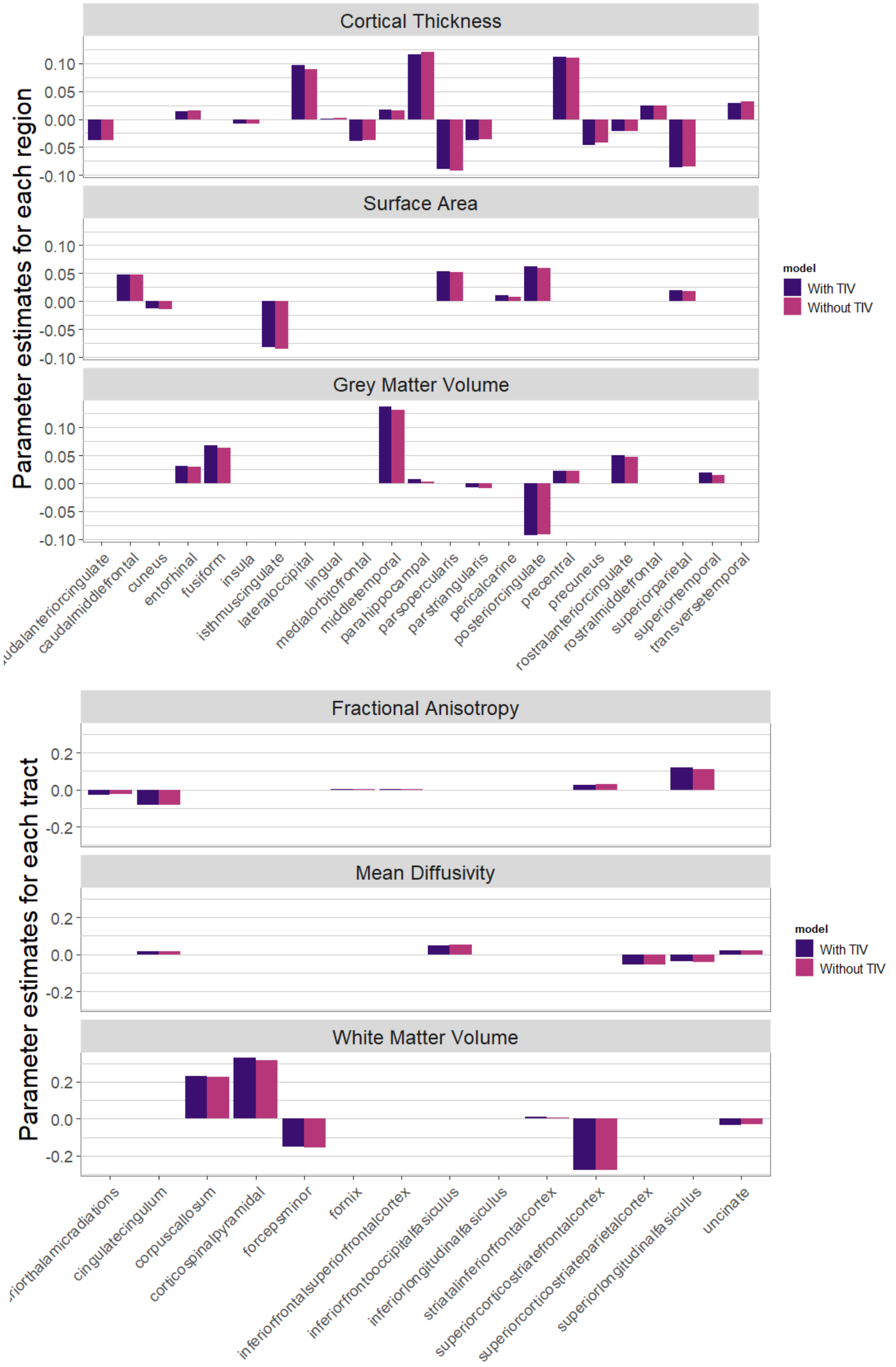

Supplement: Supplement 1 [file media-1.pdf]
