# Supplementary material for: Grey and white matter metrics demonstrate distinct and complementary prediction of differences in cognitive performance in children: Findings from ABCD (N= 11 876)
Source: bioRxiv. 2023 Mar 8:2023.03.06.529634. Preprint. [Version 1] doi: 10.1101/2023.03.06.529634 (PMC10028815; doi:10.1101/2023.03.06.529634)
Supplement: Supplementary file 1 [file media-1.docx]

**Supplementary materials**

Grey and white matter metrics demonstrate distinct and complementary prediction of differences in cognitive performance in children: Findings from ABCD (N= 11 876)

Lea C. Michel^1^, Ethan M. McCormick^1,2,3^ & Rogier A. Kievit^1^

Contents

I. Summary of the variables

II. Correlations between the 5 cognitive tasks

III. Confirmatory Factor Analysis

IV. Individual models estimating cognitive factor from one region in one metric 

i. Cortical Thickness

ii. Surface Area

iii. Grey Matter Volume

iv. Fractional Anisotropy

v. Mean Diffusivity

vi. White Matter Volume

V. Models per metric estimating cognitive factor from several regions in one metric

i. Cortical Thickness (all regions and regularized regions)

ii. Surface Area (all regions and regularized regions)

iii. Grey Matter Volume (all regions and regularized regions)

iv. Fractional Anisotropy (all regions and regularized regions)

v. Mean Diffusivity (all regions and regularized regions)

vi. White Matter Volume (all regions and regularized regions)

VI. Models per tissue estimating cognitive factor from several regions in three metrics

i. Grey Matter Metrics  (all regions and regularized regions)

ii. White Matter Metrics (all regions and regularized regions)

VII. Models with grey and white matter metrics estimating cognitive factor from several regions in the six metrics

VIII. Comparison between models with grey matter, white matter and grey & white matter metrics 

i. Information criterion

ii. Adjusted R-square

**Summary of the variables** 

*Supplementary Table 1. Characteristics of the variables used in the models. All the measures of grey matter and all the ones in white matter share the same frequency and number of missing data per tissue so we added only one measure of grey matter and one measure of white matter.*

| **Variable** | **Stats / Values** | **Freqs (% of Valid)** | **Graph** | **Missing** |
| --- | --- | --- | --- | --- |
| subjectkey [character] |  | 11 876 distinct values |  | 0 (0.0%) |
| interview_age [numeric] | Mean (sd) : 119 (7.5) min ≤ med ≤ max: 107 ≤ 119 ≤ 133 IQR (CV) : 14 (0.1) | 27 distinct values |  | 0 (0.0%) |
| sex [factor] | 1. F 2. M | 5680 ( 47.8% ) 6196 ( 52.2% ) |  | 0 (0.0%) |
| Picture_Vocabulary [numeric] | Mean (sd) : 84.5 (8.1) min ≤ med ≤ max: 29 ≤ 84 ≤ 119 IQR (CV) : 11 (0.1) | 70 distinct values |  | 147 (1.2%) |
| Flanker [numeric] | Mean (sd) : 94 (9.1) min ≤ med ≤ max: 51 ≤ 95 ≤ 116 IQR (CV) : 11 (0.1) | 68 distinct values |  | 153 (1.3%) |
| Oral_Reading_Recognition [numeric] | Mean (sd) : 90.9 (6.9) min ≤ med ≤ max: 59 ≤ 91 ≤ 119 IQR (CV) : 8 (0.1) | 63 distinct values |  | 161 (1.4%) |
| Rey_Auditory_Verbal_Learning [numeric] | Mean (sd) : 44.2 (10) min ≤ med ≤ max: 0 ≤ 45 ≤ 72 IQR (CV) : 13 (0.2) | 69 distinct values |  | 292 (2.5%) |
| Little_Man [numeric] | Mean (sd) : 58.9 (17) min ≤ med ≤ max: 0 ≤ 56.2 ≤ 100 IQR (CV) : 25 (0.3) | 32 distinct values |  | 337 (2.8%) |
| bankssts_ct [numeric] | Mean (sd) : 0.1 (0.8) min ≤ med ≤ max: -6.8 ≤ 0.1 ≤ 4.3 IQR (CV) : 1.1 (7.9) | 11521 distinct values |  | 116 (1.0%) |
| forecepsmajor_fa [numeric] | Mean (sd) : -0.1 (1) min ≤ med ≤ max: -9.3 ≤ 0 ≤ 9.4 IQR (CV) : 1.2 (-8.2) | 11107 distinct values |  | 739 (6.2%) |

*Supplementary Table 2. Characteristics of the variables used in the models for the model-building sample. All the measures of grey matter and all the ones in white matter share the same frequency and number of missing data per tissue so we added only one measure of grey matter and one measure of white matter.*

| **Variable** | **Stats / Values** | **Freqs (% of Valid)** | **Graph** | **Missing** |
| --- | --- | --- | --- | --- |
| subjectkey [character] |  | 1 781 distinct values |  | 0 (0.0%) |
| interview_age [numeric] | Mean (sd) : 118.8 (7.5) min ≤ med ≤ max: 107 ≤ 118 ≤ 133 IQR (CV) : 14 (0.1) | 27 distinct values |  | 0 (0.0%) |
| sex [factor] | 1. F 2. M | 874 ( 49.1% ) 907 ( 50.9% ) |  | 0 (0.0%) |
| Picture_Vocabulary [numeric] | Mean (sd) : 84.5 (8.1) min ≤ med ≤ max: 29 ≤ 84 ≤ 119 IQR (CV) : 11 (0.1) | 52 distinct values |  | 27 (1.5%) |
| Flanker [numeric] | Mean (sd) : 94 (9) min ≤ med ≤ max: 51 ≤ 96 ≤ 114 IQR (CV) : 10 (0.1) | 58 distinct values |  | 27 (1.5%) |
| Oral_Reading_Recognition [numeric] | Mean (sd) : 90.7 (6.9) min ≤ med ≤ max: 62 ≤ 91 ≤ 118 IQR (CV) : 8 (0.1) | 51 distinct values |  | 29 (1.6%) |
| Rey_Auditory_Verbal_ Learning [numeric] | Mean (sd) : 43.7 (10) min ≤ med ≤ max: 0 ≤ 44 ≤ 70 IQR (CV) : 14 (0.2) | 60 distinct values |  | 38 (2.1%) |
| Little_Man [numeric] | Mean (sd) : 59 (17) min ≤ med ≤ max: 9.4 ≤ 56.2 ≤ 100 IQR (CV) : 25 (0.3) | 29 distinct values |  | 50 (2.8%) |
| bankssts_ct [numeric] | Mean (sd) : 0.1 (0.9) min ≤ med ≤ max: -3.6 ≤ 0.1 ≤ 3.1 IQR (CV) : 1.1 (8.5) | 1759 distinct values |  | 18 (1.0%) |
| forecepsmajor_fa [numeric] | Mean (sd) : -0.1 (1) min ≤ med ≤ max: -7 ≤ 0 ≤ 2.2 IQR (CV) : 1.2 (-8.9) | 1666 distinct values |  | 115 (6.5%) |

*Supplementary Table 3. Characteristics of the variables used in the models for the validation sample. All the measures of grey matter and all the ones in white matter share the same frequency and number of missing data per tissue so we added only one measure of grey matter and one measure of white matter.*

| **Variable** | **Stats / Values** | **Freqs (% of Valid)** | **Graph** | **Missing** |
| --- | --- | --- | --- | --- |
| subjectkey [character] |  | 10 095 distinct values |  | 0 (0.0%) |
| interview_age [numeric] | Mean (sd) : 119 (7.5) min ≤ med ≤ max: 107 ≤ 119 ≤ 132 IQR (CV) : 14 (0.1) | 26 distinct values |  | 0 (0.0%) |
| sex [factor] | 1. F 2. M | 4806 ( 47.6% ) 5289 ( 52.4% ) |  | 0 (0.0%) |
| Picture_Vocabulary [numeric] | Mean (sd) : 84.4 (8.1) min ≤ med ≤ max: 36 ≤ 84 ≤ 119 IQR (CV) : 11 (0.1) | 69 distinct values |  | 120 (1.2%) |
| Flanker [numeric] | Mean (sd) : 94 (9.2) min ≤ med ≤ max: 53 ≤ 95 ≤ 116 IQR (CV) : 11 (0.1) | 67 distinct values |  | 126 (1.2%) |
| Oral_Reading_Recognition [numeric] | Mean (sd) : 90.9 (6.9) min ≤ med ≤ max: 59 ≤ 91 ≤ 119 IQR (CV) : 8 (0.1) | 62 distinct values |  | 132 (1.3%) |
| Rey_Auditory_Verbal_ Learning [numeric] | Mean (sd) : 44.2 (10) min ≤ med ≤ max: 0 ≤ 45 ≤ 72 IQR (CV) : 13 (0.2) | 68 distinct values |  | 254 (2.5%) |
| Little_Man [numeric] | Mean (sd) : 58.9 (17) min ≤ med ≤ max: 0 ≤ 56.2 ≤ 100 IQR (CV) : 25 (0.3) | 32 distinct values |  | 287 (2.8%) |
| bankssts_ct [numeric] | Mean (sd) : 0.1 (0.8) min ≤ med ≤ max: -6.8 ≤ 0.1 ≤ 4.3 IQR (CV) : 1.1 (7.8) | 9820 distinct values |  | 98 (1.0%) |
| forecepsmajor_fa [numeric] | Mean (sd) : -0.1 (1) min ≤ med ≤ max: -9.3 ≤ 0 ≤ 9.4 IQR (CV) : 1.2 (-8.1) | 9446 distinct values |  | 624 (6.2%) |

**Correlations between the 5 cognitive tasks**

*Supplementary Figure 1. Correlation plot of the five measures of cognition: Picture Vocabulary, Flanker, Oral Reading Recognition, Rey Auditory Verbal Learning and Little Man in the whole sample.* 

*Supplementary Table 4. Correlation table of the five measures of cognition: Picture Vocabulary, Flanker, Oral Reading Recognition, Rey Auditory Verbal Learning and Little Man in the whole sample.*

|  | Picture Vocabulary | Flanker | Oral Reading Recognition | Rey Auditory Verbal Learning | Little Man |
| --- | --- | --- | --- | --- | --- |
| Picture Vocabulary | 1 |  |  |  |  |
| Flanker | 0.26 | 1 |  |  |  |
| Oral Reading Recognition | 0.53 | 0.28 | 1 |  |  |
| Rey Auditory Verbal Learning | 0.38 | 0.24 | 0.37 | 1 |  |
| Little Man | 0.28 | 0.25 | 0.35 | 0.25 | 1 |

**Confirmatory Factor Analysis**

*Supplementary Table 5. Latent variable loadings for the confirmatory factor analysis estimating the cognitive factor*

| Path | Estimate | SE | *p* | Standardized Estimate |
| --- | --- | --- | --- | --- |
| Cognitive_factor  =~  Picture_Vocabulary | 1.000 | 0.000 | NA | 0.699 |
| Cognitive_factor  =~  Flanker | 0.582 | 0.020 | *p<0.001* | 0.406 |
| Cognitive_factor  =~  Oral_Reading_Recognition | 1.056 | 0.022 | *p<0.001* | 0.738 |
| Cognitive_factor  =~  Rey_Auditory_Verbal_Learning | 0.753 | 0.018 | *p<0.001* | 0.527 |
| Cognitive_factor  =~  Little_Man | 0.651 | 0.019 | *p<0.001* | 0.455 |

*Supplementary Figure 2. Path diagrams of a measurement model of cognitive performance based on five cognitive tasks with and without adding age as a predictor. All loadings showed are standardized and statistically significant according to the Wald test.* 

**Individual models estimating cognitive factor from one region in one metric** 

Note: Every regions is bilaterally averaged. The results reported here are based on the validation sample (85%).

**Cortical Thickness**

*Supplementary Table 6. Regression estimates for the model estimating how cortical thickness of one region predict the cognitive factor*

| Path | Estimate | SE | *p* | Standardized Estimate |
| --- | --- | --- | --- | --- |
| Cognitive_factor    ~    bankssts_ct | 0.052 | 0.010 | *p<0.001* | 0.062 |
| Cognitive_factor    ~    caudalanteriorcingulate_ct | -0.049 | 0.010 | *p<0.001* | -0.059 |
| Cognitive_factor    ~    caudalmiddlefrontal_ct | 0.030 | 0.009 | 0.001 | 0.038 |
| Cognitive_factor    ~    cuneus_ct | 0.088 | 0.009 | *p<0.001* | 0.114 |
| Cognitive_factor    ~    entorhinal_ct | 0.049 | 0.009 | *p<0.001* | 0.061 |
| Cognitive_factor    ~    fusiform_ct | 0.077 | 0.009 | *p<0.001* | 0.100 |
| Cognitive_factor    ~    inferiorparietal_ct | 0.032 | 0.009 | *p<0.001* | 0.042 |
| Cognitive_factor    ~    inferiortemporal_ct | 0.065 | 0.009 | *p<0.001* | 0.085 |
| Cognitive_factor    ~    isthmuscingulate_ct | 0.003 | 0.010 | 0.778 | 0.003 |
| Cognitive_factor    ~    lateraloccipital_ct | 0.103 | 0.009 | *p<0.001* | 0.139 |
| Cognitive_factor    ~    lateralorbitofrontal_ct | 0.020 | 0.009 | 0.027 | 0.026 |
| Cognitive_factor    ~    lingual_ct | 0.109 | 0.009 | *p<0.001* | 0.145 |
| Cognitive_factor    ~    medialorbitofrontal_ct | -0.042 | 0.009 | *p<0.001* | -0.053 |
| Cognitive_factor    ~    middletemporal_ct | 0.058 | 0.009 | *p<0.001* | 0.076 |
| Cognitive_factor    ~    parahippocampal_ct | 0.098 | 0.009 | *p<0.001* | 0.127 |
| Cognitive_factor    ~    paracentral_ct | 0.055 | 0.009 | *p<0.001* | 0.072 |
| Cognitive_factor    ~    parsopercularis_ct | -0.026 | 0.009 | 0.006 | -0.032 |
| Cognitive_factor    ~    parsorbitalis_ct | -0.012 | 0.010 | 0.238 | -0.014 |
| Cognitive_factor    ~    parstriangularis_ct | -0.021 | 0.009 | 0.029 | -0.026 |
| Cognitive_factor    ~    pericalcarine_ct | 0.086 | 0.009 | *p<0.001* | 0.113 |
| Cognitive_factor    ~    postcentral_ct | 0.066 | 0.009 | *p<0.001* | 0.087 |
| Cognitive_factor    ~    posteriorcingulate_ct | -0.011 | 0.010 | 0.268 | -0.014 |
| Cognitive_factor    ~    precentral_ct | 0.081 | 0.009 | *p<0.001* | 0.108 |
| Cognitive_factor    ~    precuneus_ct | 0.027 | 0.009 | 0.002 | 0.036 |
| Cognitive_factor    ~    rostralanteriorcingulate_ct | -0.044 | 0.010 | *p<0.001* | -0.052 |
| Cognitive_factor    ~    rostralmiddlefrontal_ct | 0.004 | 0.009 | 0.661 | 0.005 |
| Cognitive_factor    ~    superiorfrontal_ct | -0.011 | 0.009 | 0.220 | -0.014 |
| Cognitive_factor    ~    superiorparietal_ct | 0.032 | 0.009 | *p<0.001* | 0.044 |
| Cognitive_factor    ~    superiortemporal_ct | 0.044 | 0.009 | *p<0.001* | 0.059 |
| Cognitive_factor    ~    supramarginal_ct | 0.045 | 0.009 | *p<0.001* | 0.059 |
| Cognitive_factor    ~    frontalpole_ct | -0.051 | 0.010 | *p<0.001* | -0.061 |
| Cognitive_factor    ~    temporalpole_ct | 0.057 | 0.009 | *p<0.001* | 0.071 |
| Cognitive_factor    ~    transversetemporal_ct | 0.053 | 0.009 | *p<0.001* | 0.066 |
| Cognitive_factor    ~    insula_ct | 0.026 | 0.009 | 0.006 | 0.032 |

*Supplementary Figure 3. P-value of the regression parameter for how cortical thickness in each region predicts cognitive performance models estimating how the cortical thickness (arrow shown in Figure of each region of interest predict the cognitive factor  2C, main manuscript)*

*Supplementary Figure 4. Standardized parameter estimates of how the cortical thickness of each region of interest predict the cognitive factor* 

 

**Surface Area**

*Supplementary Table 7. Regression estimates for the model estimating how surface area of one region predict the cognitive factor*

| Path | Estimate | SE | *p* | Standardized Estimate |
| --- | --- | --- | --- | --- |
| Cognitive_factor    ~    bankssts_sa | 0.123 | 0.010 | *p<0.001* | 0.152 |
| Cognitive_factor    ~    caudalanteriorcingulate_sa | 0.172 | 0.011 | *p<0.001* | 0.196 |
| Cognitive_factor    ~    caudalmiddlefrontal_sa | 0.170 | 0.010 | *p<0.001* | 0.218 |
| Cognitive_factor    ~    cuneus_sa | 0.099 | 0.009 | *p<0.001* | 0.133 |
| Cognitive_factor    ~    entorhinal_sa | 0.134 | 0.009 | *p<0.001* | 0.170 |
| Cognitive_factor    ~    fusiform_sa | 0.193 | 0.009 | *p<0.001* | 0.255 |
| Cognitive_factor    ~    inferiorparietal_sa | 0.141 | 0.009 | *p<0.001* | 0.185 |
| Cognitive_factor    ~    inferiortemporal_sa | 0.201 | 0.009 | *p<0.001* | 0.265 |
| Cognitive_factor    ~    isthmuscingulate_sa | 0.118 | 0.011 | *p<0.001* | 0.153 |
| Cognitive_factor    ~    lateraloccipital_sa | 0.147 | 0.009 | *p<0.001* | 0.196 |
| Cognitive_factor    ~    lateralorbitofrontal_sa | 0.181 | 0.009 | *p<0.001* | 0.244 |
| Cognitive_factor    ~    lingual_sa | 0.107 | 0.009 | *p<0.001* | 0.145 |
| Cognitive_factor    ~    medialorbitofrontal_sa | 0.176 | 0.009 | *p<0.001* | 0.230 |
| Cognitive_factor    ~    middletemporal_sa | 0.174 | 0.009 | *p<0.001* | 0.236 |
| Cognitive_factor    ~    parahippocampal_sa | 0.123 | 0.009 | *p<0.001* | 0.157 |
| Cognitive_factor    ~    paracentral_sa | 0.127 | 0.009 | *p<0.001* | 0.162 |
| Cognitive_factor    ~    parsopercularis_sa | 0.154 | 0.010 | *p<0.001* | 0.193 |
| Cognitive_factor    ~    parsorbitalis_sa | 0.168 | 0.010 | *p<0.001* | 0.219 |
| Cognitive_factor    ~    parstriangularis_sa | 0.116 | 0.009 | *p<0.001* | 0.147 |
| Cognitive_factor    ~    pericalcarine_sa | 0.067 | 0.009 | *p<0.001* | 0.092 |
| Cognitive_factor    ~    postcentral_sa | 0.150 | 0.009 | *p<0.001* | 0.199 |
| Cognitive_factor    ~    posteriorcingulate_sa | 0.157 | 0.011 | *p<0.001* | 0.195 |
| Cognitive_factor    ~    precentral_sa | 0.173 | 0.009 | *p<0.001* | 0.230 |
| Cognitive_factor    ~    precuneus_sa | 0.159 | 0.009 | *p<0.001* | 0.216 |
| Cognitive_factor    ~    rostralanteriorcingulate_sa | 0.179 | 0.010 | *p<0.001* | 0.224 |
| Cognitive_factor    ~    rostralmiddlefrontal_sa | 0.161 | 0.009 | *p<0.001* | 0.215 |
| Cognitive_factor    ~    superiorfrontal_sa | 0.177 | 0.009 | *p<0.001* | 0.239 |
| Cognitive_factor    ~    superiorparietal_sa | 0.142 | 0.009 | *p<0.001* | 0.190 |
| Cognitive_factor    ~    superiortemporal_sa | 0.154 | 0.009 | *p<0.001* | 0.207 |
| Cognitive_factor    ~    supramarginal_sa | 0.141 | 0.009 | *p<0.001* | 0.181 |
| Cognitive_factor    ~    frontalpole_sa | 0.186 | 0.010 | *p<0.001* | 0.233 |
| Cognitive_factor    ~    temporalpole_sa | 0.115 | 0.009 | *p<0.001* | 0.147 |
| Cognitive_factor    ~    transversetemporal_sa | 0.111 | 0.010 | *p<0.001* | 0.142 |
| Cognitive_factor    ~    insula_sa | 0.158 | 0.009 | *p<0.001* | 0.211 |

*Supplementary Figure 5. P-value of the regression parameter for how surface area in each region predicts cognitive performance (arrow shown in Figure 2C, main manuscript)*

*Supplementary Figure 6. Standardized parameter estimates of how the surface area of each region of interest predict the cognitive factor* 

**Grey Matter Volume**

*Supplementary Table 8. Regression estimates for the model estimating how grey matter volume of one region predict the cognitive factor*

| Path | Estimate | SE | *p* | Standardized Estimate |
| --- | --- | --- | --- | --- |
| Cognitive_factor    ~    bankssts_gmv | 0.136 | 0.010 | *p<0.001* | 0.165 |
| Cognitive_factor    ~    caudalanteriorcingulate_gmv | 0.158 | 0.011 | *p<0.001* | 0.171 |
| Cognitive_factor    ~    caudalmiddlefrontal_gmv | 0.165 | 0.009 | *p<0.001* | 0.209 |
| Cognitive_factor    ~    cuneus_gmv | 0.118 | 0.009 | *p<0.001* | 0.157 |
| Cognitive_factor    ~    entorhinal_gmv | 0.152 | 0.010 | *p<0.001* | 0.191 |
| Cognitive_factor    ~    fusiform_gmv | 0.200 | 0.009 | *p<0.001* | 0.261 |
| Cognitive_factor    ~    inferiorparietal_gmv | 0.151 | 0.009 | *p<0.001* | 0.196 |
| Cognitive_factor    ~    inferiortemporal_gmv | 0.203 | 0.009 | *p<0.001* | 0.265 |
| Cognitive_factor    ~    isthmuscingulate_gmv | 0.117 | 0.010 | *p<0.001* | 0.150 |
| Cognitive_factor    ~    lateraloccipital_gmv | 0.184 | 0.009 | *p<0.001* | 0.245 |
| Cognitive_factor    ~    lateralorbitofrontal_gmv | 0.185 | 0.009 | *p<0.001* | 0.251 |
| Cognitive_factor    ~    lingual_gmv | 0.133 | 0.009 | *p<0.001* | 0.178 |
| Cognitive_factor    ~    medialorbitofrontal_gmv | 0.138 | 0.009 | *p<0.001* | 0.178 |
| Cognitive_factor    ~    middletemporal_gmv | 0.205 | 0.009 | *p<0.001* | 0.275 |
| Cognitive_factor    ~    parahippocampal_gmv | 0.166 | 0.010 | *p<0.001* | 0.208 |
| Cognitive_factor    ~    paracentral_gmv | 0.142 | 0.009 | *p<0.001* | 0.179 |
| Cognitive_factor    ~    parsopercularis_gmv | 0.134 | 0.010 | *p<0.001* | 0.165 |
| Cognitive_factor    ~    parsorbitalis_gmv | 0.159 | 0.010 | *p<0.001* | 0.202 |
| Cognitive_factor    ~    parstriangularis_gmv | 0.099 | 0.009 | *p<0.001* | 0.123 |
| Cognitive_factor    ~    pericalcarine_gmv | 0.090 | 0.009 | *p<0.001* | 0.123 |
| Cognitive_factor    ~    postcentral_gmv | 0.168 | 0.009 | *p<0.001* | 0.221 |
| Cognitive_factor    ~    posteriorcingulate_gmv | 0.153 | 0.010 | *p<0.001* | 0.189 |
| Cognitive_factor    ~    precentral_gmv | 0.205 | 0.009 | *p<0.001* | 0.269 |
| Cognitive_factor    ~    precuneus_gmv | 0.159 | 0.009 | *p<0.001* | 0.216 |
| Cognitive_factor    ~    rostralanteriorcingulate_gmv | 0.166 | 0.010 | *p<0.001* | 0.200 |
| Cognitive_factor    ~    rostralmiddlefrontal_gmv | 0.152 | 0.009 | *p<0.001* | 0.201 |
| Cognitive_factor    ~    superiorfrontal_gmv | 0.174 | 0.009 | *p<0.001* | 0.233 |
| Cognitive_factor    ~    superiorparietal_gmv | 0.148 | 0.009 | *p<0.001* | 0.197 |
| Cognitive_factor    ~    superiortemporal_gmv | 0.170 | 0.009 | *p<0.001* | 0.225 |
| Cognitive_factor    ~    supramarginal_gmv | 0.158 | 0.009 | *p<0.001* | 0.201 |
| Cognitive_factor    ~    frontalpole_gmv | 0.114 | 0.010 | *p<0.001* | 0.140 |
| Cognitive_factor    ~    temporalpole_gmv | 0.129 | 0.010 | *p<0.001* | 0.160 |
| Cognitive_factor    ~    transversetemporal_gmv | 0.142 | 0.010 | *p<0.001* | 0.179 |
| Cognitive_factor    ~    insula_gmv | 0.156 | 0.009 | *p<0.001* | 0.213 |

*Supplementary Figure 7. P-value of the regression parameter for how grey matter volume in each region predicts cognitive performance (arrow shown in Figure 2C, main manuscript)*

*Supplementary Figure 8. Standardized parameter estimates of how the grey matter volume of each region of interest predict the cognitive factor* 

**Fractional Anisotropy**

*Supplementary Table 9. Regression estimates for the model estimating how fractional anisotropy of one region predict the cognitive factor*

| Path | Estimate | SE | *p* | Standardized Estimate |
| --- | --- | --- | --- | --- |
| Cognitive_factor    ~    fornix_fa | 0.063 | 0.009 | *p<0.001* | 0.085 |
| Cognitive_factor    ~    cingulatecingulum_fa | 0.001 | 0.009 | 0.889 | 0.002 |
| Cognitive_factor    ~    parahippocampalcingulum_fa | 0.077 | 0.009 | *p<0.001* | 0.103 |
| Cognitive_factor    ~    corticospinalpyramidal_fa | 0.068 | 0.009 | *p<0.001* | 0.093 |
| Cognitive_factor    ~    anteriorthalamicradiations_fa | 0.032 | 0.009 | *p<0.001* | 0.043 |
| Cognitive_factor    ~    uncinate_fa | 0.033 | 0.009 | *p<0.001* | 0.044 |
| Cognitive_factor    ~    inferiorlongitudinalfasiculus_fa | 0.085 | 0.010 | *p<0.001* | 0.114 |
| Cognitive_factor    ~    inferiorfrontooccipitalfasiculus_fa | 0.067 | 0.009 | *p<0.001* | 0.092 |
| Cognitive_factor    ~    forcepsmajor_fa | 0.064 | 0.009 | *p<0.001* | 0.093 |
| Cognitive_factor    ~    forcepsminor_fa | -0.007 | 0.008 | 0.400 | -0.010 |
| Cognitive_factor    ~    corpuscallosum_fa | 0.040 | 0.009 | *p<0.001* | 0.059 |
| Cognitive_factor    ~    superiorlongitudinalfasiculus_fa | 0.102 | 0.009 | *p<0.001* | 0.141 |
| Cognitive_factor    ~    temporalsuperiorlongitudinalfasiculus_fa | 0.094 | 0.009 | *p<0.001* | 0.128 |
| Cognitive_factor    ~    parietalsuperiorlongitudinalfasiculus_fa | 0.104 | 0.009 | *p<0.001* | 0.142 |
| Cognitive_factor    ~    superiorcorticostriate_fa | 0.054 | 0.009 | *p<0.001* | 0.073 |
| Cognitive_factor    ~    superiorcorticostriatefrontalcortex_fa | 0.057 | 0.009 | *p<0.001* | 0.078 |
| Cognitive_factor    ~    superiorcorticostriateparietalcortex_fa | 0.044 | 0.009 | *p<0.001* | 0.059 |
| Cognitive_factor    ~    striatalinferiorfrontalcortex_fa | 0.014 | 0.009 | 0.121 | 0.019 |
| Cognitive_factor    ~    inferiorfrontalsuperiorfrontalcortex_fa | 0.066 | 0.009 | *p<0.001* | 0.090 |
| Cognitive_factor    ~    fornix_exfimbria_fa | 0.015 | 0.009 | 0.102 | 0.020 |

**Mean Diffusivity**

*Supplementary Table 10. Regression estimates for the model estimating how mean diffusivity of one region predict the cognitive factor*

| Path | Estimate | SE | *p* | Standardized Estimate |
| --- | --- | --- | --- | --- |
| Cognitive_factor    ~    fornix_md | -0.021 | 0.010 | 0.038 | -0.029 |
| Cognitive_factor    ~    cingulatecingulum_md | -0.020 | 0.010 | 0.033 | -0.025 |
| Cognitive_factor    ~    parahippocampalcingulum_md | -0.024 | 0.009 | 0.005 | -0.033 |
| Cognitive_factor    ~    corticospinalpyramidal_md | -0.014 | 0.008 | 0.068 | -0.019 |
| Cognitive_factor    ~    anteriorthalamicradiations_md | 0.007 | 0.008 | 0.386 | 0.010 |
| Cognitive_factor    ~    uncinate_md | -0.006 | 0.007 | 0.404 | -0.008 |
| Cognitive_factor    ~    inferiorlongitudinalfasiculus_md | -0.021 | 0.009 | 0.015 | -0.028 |
| Cognitive_factor    ~    inferiorfrontooccipitalfasiculus_md | 0.003 | 0.009 | 0.711 | 0.004 |
| Cognitive_factor    ~    forcepsmajor_md | -0.027 | 0.010 | 0.007 | -0.039 |
| Cognitive_factor    ~    forcepsminor_md | 0.021 | 0.008 | 0.012 | 0.031 |
| Cognitive_factor    ~    corpuscallosum_md | -0.013 | 0.009 | 0.131 | -0.019 |
| Cognitive_factor    ~    superiorlongitudinalfasiculus_md | -0.040 | 0.009 | *p<0.001* | -0.056 |
| Cognitive_factor    ~    temporalsuperiorlongitudinalfasiculus_md | -0.032 | 0.009 | *p<0.001* | -0.045 |
| Cognitive_factor    ~    parietalsuperiorlongitudinalfasiculus_md | -0.044 | 0.009 | *p<0.001* | -0.062 |
| Cognitive_factor    ~    superiorcorticostriate_md | -0.024 | 0.008 | 0.004 | -0.033 |
| Cognitive_factor    ~    superiorcorticostriatefrontalcortex_md | -0.015 | 0.008 | 0.062 | -0.022 |
| Cognitive_factor    ~    superiorcorticostriateparietalcortex_md | -0.030 | 0.008 | *p<0.001* | -0.042 |
| Cognitive_factor    ~    striatalinferiorfrontalcortex_md | 0.003 | 0.009 | 0.760 | 0.003 |
| Cognitive_factor    ~    inferiorfrontalsuperiorfrontalcortex_md | -0.017 | 0.009 | 0.044 | -0.024 |
| Cognitive_factor    ~    fornix_exfimbria_md | -0.007 | 0.010 | 0.481 | -0.010 |

**White Matter Volume**

*Supplementary Table 11. Regression estimates for the model estimating how white matter volume of one region predict the cognitive factor*

| Path | Estimate | SE | *p* | Standardized Estimate |
| --- | --- | --- | --- | --- |
| Cognitive_factor    ~    fornix_wmv | 0.219 | 0.010 | *p<0.001* | 0.303 |
| Cognitive_factor    ~    cingulatecingulum_wmv | 0.124 | 0.009 | *p<0.001* | 0.169 |
| Cognitive_factor    ~    parahippocampalcingulum_wmv | 0.155 | 0.010 | *p<0.001* | 0.209 |
| Cognitive_factor    ~    corticospinalpyramidal_wmv | 0.198 | 0.010 | *p<0.001* | 0.274 |
| Cognitive_factor    ~    anteriorthalamicradiations_wmv | 0.153 | 0.009 | *p<0.001* | 0.215 |
| Cognitive_factor    ~    uncinate_wmv | 0.136 | 0.009 | *p<0.001* | 0.187 |
| Cognitive_factor    ~    inferiorlongitudinalfasiculus_wmv | 0.166 | 0.010 | *p<0.001* | 0.226 |
| Cognitive_factor    ~    inferiorfrontooccipitalfasiculus_wmv | 0.174 | 0.010 | *p<0.001* | 0.240 |
| Cognitive_factor    ~    forcepsmajor_wmv | 0.161 | 0.009 | *p<0.001* | 0.230 |
| Cognitive_factor    ~    forcepsminor_wmv | 0.146 | 0.009 | *p<0.001* | 0.209 |
| Cognitive_factor    ~    corpuscallosum_wmv | 0.179 | 0.009 | *p<0.001* | 0.256 |
| Cognitive_factor    ~    superiorlongitudinalfasiculus_wmv | 0.191 | 0.010 | *p<0.001* | 0.261 |
| Cognitive_factor    ~    temporalsuperiorlongitudinalfasiculus_wmv | 0.197 | 0.010 | *p<0.001* | 0.266 |
| Cognitive_factor    ~    parietalsuperiorlongitudinalfasiculus_wmv | 0.183 | 0.010 | *p<0.001* | 0.251 |
| Cognitive_factor    ~    superiorcorticostriate_wmv | 0.172 | 0.009 | *p<0.001* | 0.237 |
| Cognitive_factor    ~    superiorcorticostriatefrontalcortex_wmv | 0.169 | 0.009 | *p<0.001* | 0.234 |
| Cognitive_factor    ~    superiorcorticostriateparietalcortex_wmv | 0.172 | 0.009 | *p<0.001* | 0.233 |
| Cognitive_factor    ~    striatalinferiorfrontalcortex_wmv | 0.149 | 0.009 | *p<0.001* | 0.203 |
| Cognitive_factor    ~    inferiorfrontalsuperiorfrontalcortex_wmv | 0.181 | 0.009 | *p<0.001* | 0.247 |
| Cognitive_factor    ~    fornix_exfimbria_wmv | 0.201 | 0.010 | *p<0.001* | 0.268 |

**Models per metric estimating cognitive factor from several regions in one metric** 

For every metric, we reported the table for the models with all the regions included as predictors and the models with only the regions that survived the regularization (the one that we used for the analyses).

**Cortical Thickness (model with all the regions)**

*Supplementary Table 12. Regression estimates for the model estimating how cortical thickness of all the regions predict the cognitive factor*

| Path | Estimate | SE | *p* | Standardized Estimate |
| --- | --- | --- | --- | --- |
| Cognitive_factor    ~    bankssts_ct | 0.018 | 0.013 | 0.164 | 0.021 |
| Cognitive_factor    ~    caudalanteriorcingulate_ct | -0.049 | 0.012 | *p<0.001* | -0.058 |
| Cognitive_factor    ~    caudalmiddlefrontal_ct | 0.025 | 0.016 | 0.124 | 0.032 |
| Cognitive_factor    ~    cuneus_ct | 0.021 | 0.014 | 0.120 | 0.028 |
| Cognitive_factor    ~    entorhinal_ct | 0.006 | 0.010 | 0.560 | 0.008 |
| Cognitive_factor    ~    fusiform_ct | 0.007 | 0.015 | 0.641 | 0.009 |
| Cognitive_factor    ~    inferiorparietal_ct | -0.053 | 0.018 | 0.003 | -0.069 |
| Cognitive_factor    ~    inferiortemporal_ct | 0.023 | 0.014 | 0.090 | 0.030 |
| Cognitive_factor    ~    isthmuscingulate_ct | -0.008 | 0.010 | 0.436 | -0.010 |
| Cognitive_factor    ~    lateraloccipital_ct | 0.091 | 0.015 | *p<0.001* | 0.123 |
| Cognitive_factor    ~    lateralorbitofrontal_ct | 0.017 | 0.013 | 0.184 | 0.022 |
| Cognitive_factor    ~    lingual_ct | 0.062 | 0.013 | *p<0.001* | 0.082 |
| Cognitive_factor    ~    medialorbitofrontal_ct | -0.036 | 0.012 | 0.004 | -0.044 |
| Cognitive_factor    ~    middletemporal_ct | 0.030 | 0.015 | 0.046 | 0.039 |
| Cognitive_factor    ~    parahippocampal_ct | 0.076 | 0.010 | *p<0.001* | 0.098 |
| Cognitive_factor    ~    paracentral_ct | 0.002 | 0.015 | 0.882 | 0.003 |
| Cognitive_factor    ~    parsopercularis_ct | -0.087 | 0.014 | *p<0.001* | -0.107 |
| Cognitive_factor    ~    parsorbitalis_ct | -0.005 | 0.013 | 0.670 | -0.007 |
| Cognitive_factor    ~    parstriangularis_ct | -0.045 | 0.014 | 0.002 | -0.055 |
| Cognitive_factor    ~    pericalcarine_ct | -0.007 | 0.012 | 0.562 | -0.009 |
| Cognitive_factor    ~    postcentral_ct | 0.055 | 0.013 | *p<0.001* | 0.072 |
| Cognitive_factor    ~    posteriorcingulate_ct | 0.010 | 0.013 | 0.429 | 0.012 |
| Cognitive_factor    ~    precentral_ct | 0.116 | 0.016 | *p<0.001* | 0.152 |
| Cognitive_factor    ~    precuneus_ct | -0.024 | 0.015 | 0.112 | -0.032 |
| Cognitive_factor    ~    rostralanteriorcingulate_ct | -0.035 | 0.012 | 0.004 | -0.042 |
| Cognitive_factor    ~    rostralmiddlefrontal_ct | 0.036 | 0.017 | 0.033 | 0.047 |
| Cognitive_factor    ~    superiorfrontal_ct | -0.074 | 0.017 | *p<0.001* | -0.101 |
| Cognitive_factor    ~    superiorparietal_ct | -0.087 | 0.018 | *p<0.001* | -0.117 |
| Cognitive_factor    ~    superiortemporal_ct | -0.015 | 0.015 | 0.321 | -0.020 |
| Cognitive_factor    ~    supramarginal_ct | -0.009 | 0.017 | 0.608 | -0.011 |
| Cognitive_factor    ~    frontalpole_ct | -0.029 | 0.012 | 0.013 | -0.034 |
| Cognitive_factor    ~    temporalpole_ct | 0.022 | 0.012 | 0.064 | 0.027 |
| Cognitive_factor    ~    transversetemporal_ct | 0.020 | 0.012 | 0.085 | 0.025 |
| Cognitive_factor    ~    insula_ct | 0.014 | 0.012 | 0.219 | 0.018 |

*Supplementary Figure 9. P-value of the models estimating how the cortical thickness of each region of interest predict the cognitive factor*

*Supplementary Figure 10. Standardized parameter estimates of how cortical thickness of each region of interest together predict the cognitive factor* 

*Supplementary Table 13. Comparison of the model with free parameters and the model with parameters constrained to a same value for the cortical thickness model with all the regions. AIC/BIC are two information criterions. Pr(>Chisq): p-value of the chi-square ratio test.*

|  | Degree of freedom | AIC | BIC | Difference in degree of freedom | Pr (>Chisq) |
| --- | --- | --- | --- | --- | --- |
| Free model | 141 | 130770.4 | 131123.7 | NA | NA |
| Constrained model | 174 | 131325.0 | 131440.3 | 33 | *p<0.001* |

**Cortical Thickness (model with the regularized regions)**

*Supplementary Table 14. Regression estimates for the model estimating how cortical thickness of the regularized regions predict the cognitive factor*

| Path | Estimate | SE | *p* | Standardized Estimate |
| --- | --- | --- | --- | --- |
| Cognitive_factor    ~    caudalanteriorcingulate_ct | -0.053 | 0.012 | *p<0.001* | -0.063 |
| Cognitive_factor    ~    caudalmiddlefrontal_ct | 0.074 | 0.015 | *p<0.001* | 0.095 |
| Cognitive_factor    ~    fusiform_ct | 0.048 | 0.014 | *p<0.001* | 0.062 |
| Cognitive_factor    ~    isthmuscingulate_ct | -0.012 | 0.010 | 0.235 | -0.015 |
| Cognitive_factor    ~    lateraloccipital_ct | 0.109 | 0.014 | *p<0.001* | 0.148 |
| Cognitive_factor    ~    lateralorbitofrontal_ct | 0.021 | 0.013 | 0.101 | 0.027 |
| Cognitive_factor    ~    medialorbitofrontal_ct | -0.044 | 0.012 | *p<0.001* | -0.055 |
| Cognitive_factor    ~    parahippocampal_ct | 0.081 | 0.010 | *p<0.001* | 0.104 |
| Cognitive_factor    ~    paracentral_ct | 0.047 | 0.014 | 0.001 | 0.060 |
| Cognitive_factor    ~    parsopercularis_ct | -0.088 | 0.013 | *p<0.001* | -0.108 |
| Cognitive_factor    ~    parsorbitalis_ct | -0.019 | 0.013 | 0.122 | -0.024 |
| Cognitive_factor    ~    pericalcarine_ct | 0.032 | 0.011 | 0.002 | 0.042 |
| Cognitive_factor    ~    postcentral_ct | 0.036 | 0.012 | 0.003 | 0.048 |
| Cognitive_factor    ~    posteriorcingulate_ct | 0.001 | 0.013 | 0.949 | 0.001 |
| Cognitive_factor    ~    precuneus_ct | -0.027 | 0.015 | 0.073 | -0.036 |
| Cognitive_factor    ~    rostralanteriorcingulate_ct | -0.035 | 0.012 | 0.005 | -0.041 |
| Cognitive_factor    ~    rostralmiddlefrontal_ct | 0.010 | 0.016 | 0.512 | 0.014 |
| Cognitive_factor    ~    superiorfrontal_ct | -0.082 | 0.017 | *p<0.001* | -0.111 |
| Cognitive_factor    ~    superiorparietal_ct | -0.083 | 0.016 | *p<0.001* | -0.113 |
| Cognitive_factor    ~    superiortemporal_ct | 0.000 | 0.014 | 0.995 | 0.000 |
| Cognitive_factor    ~    transversetemporal_ct | 0.024 | 0.012 | 0.042 | 0.029 |
| Cognitive_factor    ~    insula_ct | 0.028 | 0.012 | 0.015 | 0.035 |

 

*Supplementary Figure 11. P-value of the models estimating how the cortical thickness of each regularized region of interest together predict the cognitive factor* 

*Supplementary Figure 12. Standardized parameter estimates of how the cortical thickness of each regularized region of interest together predict the cognitive factor* 

*Supplementary Table 15. Comparison of the model with free parameters and the model with parameters constrained to a same value for the cortical thickness model with the regularized regions. AIC/BIC are two information criterions. Pr(>Chisq): p-value of the chi-square ratio test.*

|  | Degree of freedom | AIC | BIC | Difference in degree of freedom | Pr (>Chisq) |
| --- | --- | --- | --- | --- | --- |
| Free model | 93 | 130882.5 | 131149.2 | NA | NA |
| Constrained model | 114 | 131340.6 | 131456.0 | 21 | *p<0.001* |

**Surface Area (model with all the regions)**

*Supplementary Table 16. Regression estimates for the model estimating how surface area of all the regions predict the cognitive factor*

| Path | Estimate | SE | *p* | Standardized Estimate |
| --- | --- | --- | --- | --- |
| Cognitive_factor    ~    bankssts_sa | -0.038 | 0.016 | 0.014 | -0.047 |
| Cognitive_factor    ~    caudalanteriorcingulate_sa | 0.041 | 0.017 | 0.018 | 0.046 |
| Cognitive_factor    ~    caudalmiddlefrontal_sa | 0.032 | 0.015 | 0.036 | 0.041 |
| Cognitive_factor    ~    cuneus_sa | -0.025 | 0.018 | 0.151 | -0.033 |
| Cognitive_factor    ~    entorhinal_sa | 0.071 | 0.012 | *p<0.001* | 0.089 |
| Cognitive_factor    ~    fusiform_sa | 0.054 | 0.017 | 0.001 | 0.071 |
| Cognitive_factor    ~    inferiorparietal_sa | -0.028 | 0.015 | 0.070 | -0.035 |
| Cognitive_factor    ~    inferiortemporal_sa | 0.090 | 0.020 | *p<0.001* | 0.118 |
| Cognitive_factor    ~    isthmuscingulate_sa | -0.040 | 0.015 | 0.006 | -0.051 |
| Cognitive_factor    ~    lateraloccipital_sa | 0.010 | 0.015 | 0.514 | 0.013 |
| Cognitive_factor    ~    lateralorbitofrontal_sa | 0.059 | 0.017 | 0.001 | 0.079 |
| Cognitive_factor    ~    lingual_sa | 0.030 | 0.017 | 0.077 | 0.039 |
| Cognitive_factor    ~    medialorbitofrontal_sa | 0.001 | 0.016 | 0.967 | 0.001 |
| Cognitive_factor    ~    middletemporal_sa | 0.013 | 0.023 | 0.568 | 0.018 |
| Cognitive_factor    ~    parahippocampal_sa | -0.001 | 0.012 | 0.955 | -0.001 |
| Cognitive_factor    ~    paracentral_sa | -0.040 | 0.014 | 0.004 | -0.051 |
| Cognitive_factor    ~    parsopercularis_sa | 0.045 | 0.014 | 0.002 | 0.056 |
| Cognitive_factor    ~    parsorbitalis_sa | 0.035 | 0.016 | 0.032 | 0.045 |
| Cognitive_factor    ~    parstriangularis_sa | -0.062 | 0.016 | *p<0.001* | -0.078 |
| Cognitive_factor    ~    pericalcarine_sa | -0.004 | 0.018 | 0.828 | -0.005 |
| Cognitive_factor    ~    postcentral_sa | -0.043 | 0.017 | 0.011 | -0.057 |
| Cognitive_factor    ~    posteriorcingulate_sa | 0.005 | 0.017 | 0.743 | 0.007 |
| Cognitive_factor    ~    precentral_sa | 0.029 | 0.017 | 0.084 | 0.039 |
| Cognitive_factor    ~    precuneus_sa | 0.020 | 0.016 | 0.202 | 0.027 |
| Cognitive_factor    ~    rostralanteriorcingulate_sa | 0.008 | 0.016 | 0.634 | 0.010 |
| Cognitive_factor    ~    rostralmiddlefrontal_sa | -0.013 | 0.016 | 0.421 | -0.017 |
| Cognitive_factor    ~    superiorfrontal_sa | 0.024 | 0.018 | 0.195 | 0.032 |
| Cognitive_factor    ~    superiorparietal_sa | 0.021 | 0.015 | 0.160 | 0.028 |
| Cognitive_factor    ~    superiortemporal_sa | 0.016 | 0.021 | 0.442 | 0.021 |
| Cognitive_factor    ~    supramarginal_sa | -0.008 | 0.015 | 0.620 | -0.010 |
| Cognitive_factor    ~    frontalpole_sa | 0.050 | 0.014 | *p<0.001* | 0.062 |
| Cognitive_factor    ~    temporalpole_sa | -0.096 | 0.014 | *p<0.001* | -0.121 |
| Cognitive_factor    ~    transversetemporal_sa | -0.033 | 0.015 | 0.029 | -0.041 |
| Cognitive_factor    ~    insula_sa | 0.039 | 0.015 | 0.007 | 0.052 |

*Supplementary Figure 13. P-value of the models estimating how the surface area of each region of interest predict the cognitive factor*

*Supplementary Figure 14. Standardized parameter estimates of how the surface area of each region of interest together predict the cognitive factor* 

*Supplementary Table 17. Comparison of the model with free parameters and the model with parameters constrained to a same value for the surface area model with all the regions. AIC/BIC are two information criterions. Pr(>Chisq): p-value of the chi-square ratio test.*

|  | Degree of freedom | AIC | BIC | Difference in degree of freedom | Pr (>Chisq) |
| --- | --- | --- | --- | --- | --- |
| Free model | 141 | 130584.1 | 130937.4 | NA | NA |
| Constrained model | 174 | 130790.7 | 130906.1 | 33 | *p<0.001* |

**Surface Area (model with the regularized regions)**

*Supplementary Table 18. Regression estimates for the model estimating how surface area of the regularized regions predict the cognitive factor*

| Path | Estimate | SE | *p* | Standardized Estimate |
| --- | --- | --- | --- | --- |
| Cognitive_factor    ~    caudalanteriorcingulate_sa | 0.309 | 0.130 | 0.017 | 0.042 |
| Cognitive_factor    ~    cuneus_sa | -0.153 | 0.142 | 0.281 | -0.025 |
| Cognitive_factor    ~    entorhinal_sa | 0.615 | 0.094 | *p<0.001* | 0.095 |
| Cognitive_factor    ~    fusiform_sa | 0.417 | 0.134 | 0.002 | 0.067 |
| Cognitive_factor    ~    inferiorparietal_sa | -0.297 | 0.113 | 0.008 | -0.047 |
| Cognitive_factor    ~    inferiortemporal_sa | 0.840 | 0.164 | *p<0.001* | 0.135 |
| Cognitive_factor    ~    isthmuscingulate_sa | -0.307 | 0.114 | 0.007 | -0.049 |
| Cognitive_factor    ~    lateraloccipital_sa | 0.076 | 0.121 | 0.531 | 0.012 |
| Cognitive_factor    ~    lingual_sa | 0.270 | 0.136 | 0.047 | 0.044 |
| Cognitive_factor    ~    medialorbitofrontal_sa | 0.325 | 0.118 | 0.006 | 0.052 |
| Cognitive_factor    ~    middletemporal_sa | 0.008 | 0.178 | 0.965 | 0.001 |
| Cognitive_factor    ~    parahippocampal_sa | -0.019 | 0.097 | 0.847 | -0.003 |
| Cognitive_factor    ~    paracentral_sa | -0.268 | 0.109 | 0.014 | -0.041 |
| Cognitive_factor    ~    parsopercularis_sa | 0.191 | 0.096 | 0.046 | 0.029 |
| Cognitive_factor    ~    parsorbitalis_sa | 0.267 | 0.111 | 0.016 | 0.042 |
| Cognitive_factor    ~    pericalcarine_sa | -0.058 | 0.149 | 0.697 | -0.010 |
| Cognitive_factor    ~    postcentral_sa | -0.312 | 0.124 | 0.012 | -0.051 |
| Cognitive_factor    ~    precentral_sa | 0.530 | 0.123 | *p<0.001* | 0.086 |
| Cognitive_factor    ~    precuneus_sa | 0.300 | 0.112 | 0.008 | 0.050 |
| Cognitive_factor    ~    rostralanteriorcingulate_sa | 0.161 | 0.132 | 0.224 | 0.024 |
| Cognitive_factor    ~    rostralmiddlefrontal_sa | 0.016 | 0.111 | 0.886 | 0.003 |
| Cognitive_factor    ~    superiortemporal_sa | 0.048 | 0.164 | 0.767 | 0.008 |
| Cognitive_factor    ~    temporalpole_sa | -0.778 | 0.110 | *p<0.001* | -0.120 |
| Cognitive_factor    ~    transversetemporal_sa | -0.264 | 0.121 | 0.029 | -0.041 |
| Cognitive_factor    ~    insula_sa | 0.326 | 0.111 | 0.003 | 0.053 |

*Supplementary Figure 15. P-value of the models estimating how the surface area of each regularized region of interest together predict the cognitive factor* 

*Supplementary Figure 16. Standardized parameter estimates of how the surface area of each regularized region of interest together predict the cognitive factor* 

*Supplementary Table 19. Comparison of the model with free parameters and the model with parameters constrained to a same value for the surface area model with the regularized regions. AIC/BIC are two information criterions. Pr(>Chisq): p-value of the chi-square ratio test.*

|  | Degree of freedom | AIC | BIC | Difference in degree of freedom | Pr (>Chisq) |
| --- | --- | --- | --- | --- | --- |
| Free model | 77 | 130722.0 | 130959.9 | NA | NA |
| Constrained model | 94 | 130831.8 | 130947.1 | 17 | *p<0.001* |

**Grey Matter Volume (model with all the regions)**

*Supplementary Table 20. Regression estimates for the model estimating how grey matter volume of all the regions predict the cognitive factor*

| Path | Estimate | SE | *p* | Standardized Estimate |
| --- | --- | --- | --- | --- |
| Cognitive_factor    ~    bankssts_gmv | -0.038 | 0.014 | 0.008 | -0.045 |
| Cognitive_factor    ~    caudalanteriorcingulate_gmv | 0.029 | 0.015 | 0.063 | 0.031 |
| Cognitive_factor    ~    caudalmiddlefrontal_gmv | 0.019 | 0.013 | 0.152 | 0.024 |
| Cognitive_factor    ~    cuneus_gmv | -0.012 | 0.016 | 0.445 | -0.016 |
| Cognitive_factor    ~    entorhinal_gmv | 0.047 | 0.012 | *p<0.001* | 0.058 |
| Cognitive_factor    ~    fusiform_gmv | 0.054 | 0.014 | *p<0.001* | 0.070 |
| Cognitive_factor    ~    inferiorparietal_gmv | -0.028 | 0.014 | 0.050 | -0.036 |
| Cognitive_factor    ~    inferiortemporal_gmv | 0.035 | 0.015 | 0.020 | 0.045 |
| Cognitive_factor    ~    isthmuscingulate_gmv | -0.064 | 0.014 | *p<0.001* | -0.080 |
| Cognitive_factor    ~    lateraloccipital_gmv | 0.047 | 0.014 | 0.001 | 0.061 |
| Cognitive_factor    ~    lateralorbitofrontal_gmv | 0.064 | 0.017 | *p<0.001* | 0.086 |
| Cognitive_factor    ~    lingual_gmv | 0.010 | 0.014 | 0.473 | 0.014 |
| Cognitive_factor    ~    medialorbitofrontal_gmv | -0.074 | 0.015 | *p<0.001* | -0.095 |
| Cognitive_factor    ~    middletemporal_gmv | 0.134 | 0.016 | *p<0.001* | 0.178 |
| Cognitive_factor    ~    parahippocampal_gmv | 0.063 | 0.011 | *p<0.001* | 0.077 |
| Cognitive_factor    ~    paracentral_gmv | -0.030 | 0.013 | 0.025 | -0.037 |
| Cognitive_factor    ~    parsopercularis_gmv | 0.013 | 0.013 | 0.299 | 0.016 |
| Cognitive_factor    ~    parsorbitalis_gmv | 0.014 | 0.014 | 0.324 | 0.017 |
| Cognitive_factor    ~    parstriangularis_gmv | -0.035 | 0.014 | 0.010 | -0.043 |
| Cognitive_factor    ~    pericalcarine_gmv | 0.009 | 0.015 | 0.555 | 0.012 |
| Cognitive_factor    ~    postcentral_gmv | -0.022 | 0.014 | 0.106 | -0.029 |
| Cognitive_factor    ~    posteriorcingulate_gmv | -0.024 | 0.014 | 0.095 | -0.029 |
| Cognitive_factor    ~    precentral_gmv | 0.082 | 0.015 | *p<0.001* | 0.107 |
| Cognitive_factor    ~    precuneus_gmv | 0.005 | 0.016 | 0.769 | 0.006 |
| Cognitive_factor    ~    rostralanteriorcingulate_gmv | -0.006 | 0.015 | 0.691 | -0.007 |
| Cognitive_factor    ~    rostralmiddlefrontal_gmv | -0.013 | 0.014 | 0.363 | -0.017 |
| Cognitive_factor    ~    superiorfrontal_gmv | 0.011 | 0.016 | 0.502 | 0.015 |
| Cognitive_factor    ~    superiorparietal_gmv | 0.015 | 0.014 | 0.294 | 0.020 |
| Cognitive_factor    ~    superiortemporal_gmv | -0.010 | 0.016 | 0.546 | -0.013 |
| Cognitive_factor    ~    supramarginal_gmv | -0.006 | 0.014 | 0.668 | -0.008 |
| Cognitive_factor    ~    frontalpole_gmv | -0.008 | 0.012 | 0.499 | -0.010 |
| Cognitive_factor    ~    temporalpole_gmv | 0.002 | 0.012 | 0.860 | 0.003 |
| Cognitive_factor    ~    transversetemporal_gmv | 0.025 | 0.013 | 0.063 | 0.031 |
| Cognitive_factor    ~    insula_gmv | 0.005 | 0.014 | 0.729 | 0.007 |

*Supplementary Figure 17. P-value of the models estimating how the grey matter volume of each region of interest predict the cognitive factor*

*Supplementary Figure 18. Standardized parameter estimates of how the grey matter volume of each region of interest together predict the cognitive factor* 

*Supplementary Table 21. Comparison of the model with free parameters and the model with parameters constrained to a same value for the grey matter volume model with all the regions. AIC/BIC are two information criterions. Pr(>Chisq): p-value of the chi-square ratio test.*

|  | Degree of freedom | AIC | BIC | Difference in degree of freedom | Pr (>Chisq) |
| --- | --- | --- | --- | --- | --- |
| Free model | 141 | 130436.9 | 130790.2 | NA | NA |
| Constrained model | 174 | 130700.0 | 130815.4 | 33 | *p<0.001* |

**Grey Matter Volume (model with the regularized regions)**

*Supplementary Table 22. Regression estimates for the model estimating how grey matter volume of the regularized regions predict the cognitive factor*

| Path | Estimate | SE | *p* | Standardized Estimate |
| --- | --- | --- | --- | --- |
| Cognitive_factor    ~    entorhinal_gmv | 0.041 | 0.011 | *p<0.001* | 0.051 |
| Cognitive_factor    ~    fusiform_gmv | 0.056 | 0.014 | *p<0.001* | 0.073 |
| Cognitive_factor    ~    inferiorparietal_gmv | -0.002 | 0.012 | 0.859 | -0.003 |
| Cognitive_factor    ~    lateraloccipital_gmv | 0.060 | 0.013 | *p<0.001* | 0.080 |
| Cognitive_factor    ~    parahippocampal_gmv | 0.065 | 0.011 | *p<0.001* | 0.081 |
| Cognitive_factor    ~    paracentral_gmv | -0.018 | 0.012 | 0.132 | -0.022 |
| Cognitive_factor    ~    pericalcarine_gmv | -0.002 | 0.010 | 0.848 | -0.002 |
| Cognitive_factor    ~    precentral_gmv | 0.106 | 0.013 | *p<0.001* | 0.139 |
| Cognitive_factor    ~    precuneus_gmv | -0.003 | 0.013 | 0.826 | -0.004 |
| Cognitive_factor    ~    supramarginal_gmv | 0.010 | 0.013 | 0.438 | 0.013 |
| Cognitive_factor    ~    insula_gmv | 0.014 | 0.012 | 0.237 | 0.019 |

*Supplementary Figure 19. P-value of the models estimating how the grey matter volume of each regularized region of interest together predict the cognitive factor* 

*Supplementary Figure 20. Standardized parameter estimates of how the grey matter volume of each regularized region of interest together predict the cognitive factor* 

*Supplementary Table 23. Comparison of the model with free parameters and the model with parameters constrained to a same value for the grey matter volume model with the regularized regions. AIC/BIC are two information criterions. Pr(>Chisq): p-value of the chi-square ratio test.*

|  | Degree of freedom | AIC | BIC | Difference in degree of freedom | Pr (>Chisq) |
| --- | --- | --- | --- | --- | --- |
| Free model | 49 | 130585.3 | 130772.7 | NA | NA |
| Constrained model | 59 | 130659.9 | 130775.2 | 10 | *p<0.001* |

**Fractional Anisotropy (model with all the regions)**

*Supplementary Table 24. Regression estimates for the model estimating how fractional anisotropy of all the regions predict the cognitive factor*

| Path | Estimate | SE | *p* | Standardized Estimate |
| --- | --- | --- | --- | --- |
| Cognitive_factor    ~    fornix_fa | 0.092 | 0.016 | *p<0.001* | 0.120 |
| Cognitive_factor    ~    cingulatecingulum_fa | -0.091 | 0.012 | *p<0.001* | -0.119 |
| Cognitive_factor    ~    parahippocampalcingulum_fa | 0.046 | 0.012 | *p<0.001* | 0.061 |
| Cognitive_factor    ~    corticospinalpyramidal_fa | 0.084 | 0.017 | *p<0.001* | 0.113 |
| Cognitive_factor    ~    anteriorthalamicradiations_fa | 0.008 | 0.013 | 0.545 | 0.011 |
| Cognitive_factor    ~    uncinate_fa | -0.047 | 0.017 | 0.007 | -0.062 |
| Cognitive_factor    ~    inferiorlongitudinalfasiculus_fa | 0.021 | 0.016 | 0.181 | 0.027 |
| Cognitive_factor    ~    inferiorfrontooccipitalfasiculus_fa | 0.053 | 0.018 | 0.003 | 0.071 |
| Cognitive_factor    ~    forcepsmajor_fa | 0.038 | 0.018 | 0.035 | 0.054 |
| Cognitive_factor    ~    forcepsminor_fa | -0.107 | 0.020 | *p<0.001* | -0.153 |
| Cognitive_factor    ~    corpuscallosum_fa | 0.012 | 0.031 | 0.696 | 0.017 |
| Cognitive_factor    ~    superiorlongitudinalfasiculus_fa | 0.316 | 0.136 | 0.020 | 0.424 |
| Cognitive_factor    ~    temporalsuperiorlongitudinalfasiculus_fa | -0.195 | 0.073 | 0.007 | -0.260 |
| Cognitive_factor    ~    parietalsuperiorlongitudinalfasiculus_fa | 0.035 | 0.079 | 0.659 | 0.046 |
| Cognitive_factor    ~    superiorcorticostriate_fa | 0.879 | 0.116 | *p<0.001* | 1.162 |
| Cognitive_factor    ~    superiorcorticostriatefrontalcortex_fa | -0.243 | 0.051 | *p<0.001* | -0.323 |
| Cognitive_factor    ~    superiorcorticostriateparietalcortex_fa | -0.732 | 0.081 | *p<0.001* | -0.958 |
| Cognitive_factor    ~    striatalinferiorfrontalcortex_fa | -0.080 | 0.016 | *p<0.001* | -0.104 |
| Cognitive_factor    ~    inferiorfrontalsuperiorfrontalcortex_fa | 0.013 | 0.019 | 0.486 | 0.017 |
| Cognitive_factor    ~    fornix_exfimbria_fa | -0.071 | 0.016 | *p<0.001* | -0.090 |

*Supplementary Table 25. Comparison of the model with free parameters and the model with parameters constrained to a same value for the fractional anisotropy model with all the regions. AIC/BIC are two information criterions. Pr(>Chisq): p-value of the chi-square ratio test.*

|  | Degree of freedom | AIC | BIC | Difference in degree of freedom | Pr (>Chisq) |
| --- | --- | --- | --- | --- | --- |
| Free model | 85 | 123523.5 | 123774.0 | NA | NA |
| Constrained model | 104 | 123960.7 | 124075.2 | 19 | *p<0.001* |

**Fractional Anisotropy (model with the regularized regions)**

*Supplementary Table 26. Regression estimates for the model estimating how fractional anisotropy of the regularized regions predict the cognitive factor*

| Path | Estimate | SE | *p* | Standardized Estimate |
| --- | --- | --- | --- | --- |
| Cognitive_factor    ~    forcepsminor_fa | -0.153 | 0.017 | *p<0.001* | -0.221 |
| Cognitive_factor    ~    corpuscallosum_fa | 0.076 | 0.020 | *p<0.001* | 0.111 |
| Cognitive_factor    ~    cingulatecingulum_fa | -0.070 | 0.012 | *p<0.001* | -0.093 |
| Cognitive_factor    ~    corticospinalpyramidal_fa | 0.037 | 0.012 | 0.002 | 0.051 |
| Cognitive_factor    ~    anteriorthalamicradiations_fa | 0.001 | 0.011 | 0.946 | 0.001 |
| Cognitive_factor    ~    inferiorfrontooccipitalfasiculus_fa | 0.047 | 0.013 | *p<0.001* | 0.064 |
| Cognitive_factor    ~    temporalsuperiorlongitudinalfasiculus_fa | 0.117 | 0.014 | *p<0.001* | 0.160 |

*Supplementary Table 27. Comparison of the model with free parameters and the model with parameters constrained to a same value for the fractional anisotropy model with the regularized regions. AIC/BIC are two information criterions. Pr(>Chisq): p-value of the chi-square ratio test.*

|  | Degree of freedom | AIC | BIC | Difference in degree of freedom | Pr (>Chisq) |
| --- | --- | --- | --- | --- | --- |
| Free model | 33 | 123787.5 | 123944.9 | NA | NA |
| Constrained model | 39 | 124019.9 | 124134.4 | 6 | *p<0.001* |

**Mean Diffusivity (model with all the regions)**

*Supplementary Table 28. Regression estimates for the model estimating how mean diffusivity of all the regions predict the cognitive factor*

| Path | Estimate | SE | *p* | Standardized Estimate |
| --- | --- | --- | --- | --- |
| Cognitive_factor    ~    fornix_md | -0.070 | 0.023 | 0.002 | -0.095 |
| Cognitive_factor    ~    cingulatecingulum_md | 0.022 | 0.016 | 0.175 | 0.025 |
| Cognitive_factor    ~    parahippocampalcingulum_md | -0.029 | 0.015 | 0.049 | -0.039 |
| Cognitive_factor    ~    corticospinalpyramidal_md | 0.009 | 0.016 | 0.569 | 0.012 |
| Cognitive_factor    ~    anteriorthalamicradiations_md | -0.003 | 0.018 | 0.864 | -0.004 |
| Cognitive_factor    ~    uncinate_md | -0.018 | 0.019 | 0.361 | -0.024 |
| Cognitive_factor    ~    inferiorlongitudinalfasiculus_md | -0.061 | 0.021 | 0.003 | -0.079 |
| Cognitive_factor    ~    inferiorfrontooccipitalfasiculus_md | 0.119 | 0.024 | *p<0.001* | 0.153 |
| Cognitive_factor    ~    forcepsmajor_md | -0.013 | 0.018 | 0.456 | -0.019 |
| Cognitive_factor    ~    forcepsminor_md | 0.085 | 0.018 | *p<0.001* | 0.123 |
| Cognitive_factor    ~    corpuscallosum_md | -0.060 | 0.026 | 0.019 | -0.084 |
| Cognitive_factor    ~    superiorlongitudinalfasiculus_md | -0.647 | 0.195 | 0.001 | -0.865 |
| Cognitive_factor    ~    temporalsuperiorlongitudinalfasiculus_md | 0.386 | 0.103 | *p<0.001* | 0.517 |
| Cognitive_factor    ~    parietalsuperiorlongitudinalfasiculus_md | 0.179 | 0.109 | 0.102 | 0.240 |
| Cognitive_factor    ~    superiorcorticostriate_md | 0.286 | 0.149 | 0.055 | 0.374 |
| Cognitive_factor    ~    superiorcorticostriatefrontalcortex_md | 0.008 | 0.070 | 0.907 | 0.011 |
| Cognitive_factor    ~    superiorcorticostriateparietalcortex_md | -0.306 | 0.103 | 0.003 | -0.400 |
| Cognitive_factor    ~    striatalinferiorfrontalcortex_md | -0.044 | 0.025 | 0.078 | -0.055 |
| Cognitive_factor    ~    inferiorfrontalsuperiorfrontalcortex_md | 0.063 | 0.031 | 0.038 | 0.084 |
| Cognitive_factor    ~    fornix_exfimbria_md | 0.075 | 0.021 | *p<0.001* | 0.101 |

*Supplementary Table 29. Comparison of the model with free parameters and the model with parameters constrained to a same value for the mean diffusivity model with all the regions. AIC/BIC are two information criterions. Pr(>Chisq): p-value of the chi-square ratio test.*

|  | Degree of freedom | AIC | BIC | Difference in degree of freedom | Pr (>Chisq) |
| --- | --- | --- | --- | --- | --- |
| Free model | 85 | 123896.3 | 124146.7 | NA | NA |
| Constrained model | 104 | 124029.2 | 124143.7 | 19 | *p<0.001* |

**Mean Diffusivity (model with the regularized regions)**

*Supplementary Table 30. Regression estimates for the model estimating how mean diffusivity of the regularized regions predict the cognitive factor*

| Path | Estimate | SE | *p* | Standardized Estimate |
| --- | --- | --- | --- | --- |
| Cognitive_factor    ~    forcepsminor_md | 0.072 | 0.016 | *p<0.001* | 0.104 |
| Cognitive_factor    ~    corpuscallosum_md | -0.054 | 0.018 | 0.003 | -0.076 |
| Cognitive_factor    ~    parahippocampalcingulum_md | -0.037 | 0.011 | 0.001 | -0.050 |
| Cognitive_factor    ~    anteriorthalamicradiations_md | 0.031 | 0.016 | 0.047 | 0.043 |
| Cognitive_factor    ~    superiorcorticostriate_md | -0.032 | 0.016 | 0.038 | -0.042 |

*Supplementary Table 31. Comparison of the model with free parameters and the model with parameters constrained to a same value for the mean diffusivity model with the regularized regions. AIC/BIC are two information criterions. Pr(>Chisq): p-value of the chi-square ratio test.*

|  | Degree of freedom | AIC | BIC | Difference in degree of freedom | Pr (>Chisq) |
| --- | --- | --- | --- | --- | --- |
| Free model | 25 | 123984.6 | 124127.8 | NA | NA |
| Constrained model | 29 | 124034.6 | 124149.1 | 4 | *p<0.001* |

**White Matter Volume (model with all the regions)**

*Supplementary Table 32. Regression estimates for the model estimating how white matter volume of all the regions predict the cognitive factor*

| Path | Estimate | SE | *p* | Standardized Estimate |
| --- | --- | --- | --- | --- |
| Cognitive_factor    ~    fornix_wmv | 0.174 | 0.028 | *p<0.001* | 0.240 |
| Cognitive_factor    ~    cingulatecingulum_wmv | -0.084 | 0.016 | *p<0.001* | -0.112 |
| Cognitive_factor    ~    parahippocampalcingulum_wmv | 0.002 | 0.013 | 0.879 | 0.003 |
| Cognitive_factor    ~    corticospinalpyramidal_wmv | 0.210 | 0.024 | *p<0.001* | 0.287 |
| Cognitive_factor    ~    anteriorthalamicradiations_wmv | 0.074 | 0.024 | 0.002 | 0.103 |
| Cognitive_factor    ~    uncinate_wmv | -0.029 | 0.016 | 0.073 | -0.039 |
| Cognitive_factor    ~    inferiorlongitudinalfasiculus_wmv | 0.020 | 0.017 | 0.245 | 0.026 |
| Cognitive_factor    ~    inferiorfrontooccipitalfasiculus_wmv | 0.015 | 0.027 | 0.569 | 0.021 |
| Cognitive_factor    ~    forcepsmajor_wmv | -0.027 | 0.019 | 0.158 | -0.038 |
| Cognitive_factor    ~    forcepsminor_wmv | -0.079 | 0.026 | 0.002 | -0.112 |
| Cognitive_factor    ~    corpuscallosum_wmv | 0.095 | 0.040 | 0.017 | 0.134 |
| Cognitive_factor    ~    superiorlongitudinalfasiculus_wmv | -0.020 | 0.081 | 0.809 | -0.027 |
| Cognitive_factor    ~    temporalsuperiorlongitudinalfasiculus_wmv | 0.113 | 0.054 | 0.037 | 0.151 |
| Cognitive_factor    ~    parietalsuperiorlongitudinalfasiculus_wmv | -0.017 | 0.043 | 0.691 | -0.023 |
| Cognitive_factor    ~    superiorcorticostriate_wmv | -0.082 | 0.087 | 0.348 | -0.111 |
| Cognitive_factor    ~    superiorcorticostriatefrontalcortex_wmv | -0.153 | 0.051 | 0.003 | -0.209 |
| Cognitive_factor    ~    superiorcorticostriateparietalcortex_wmv | -0.009 | 0.056 | 0.870 | -0.012 |
| Cognitive_factor    ~    striatalinferiorfrontalcortex_wmv | -0.050 | 0.019 | 0.008 | -0.067 |
| Cognitive_factor    ~    inferiorfrontalsuperiorfrontalcortex_wmv | 0.101 | 0.023 | *p<0.001* | 0.136 |
| Cognitive_factor    ~    fornix_exfimbria_wmv | -0.034 | 0.027 | 0.204 | -0.045 |

*Supplementary Table 33. Comparison of the model with free parameters and the model with parameters constrained to a same value for the white matter volume model with all the regions. AIC/BIC are two information criterions. Pr(>Chisq): p-value of the chi-square ratio test.*

|  | Degree of freedom | AIC | BIC | Difference in degree of freedom | Pr (>Chisq) |
| --- | --- | --- | --- | --- | --- |
| Free model | 85 | 123233.9 | 123484.4 | NA | NA |
| Constrained model | 104 | 123509.8 | 123624.3 | 19 | *p<0.001* |

**White Matter Volume (model with the regularized regions)**

*Supplementary Table 34. Regression estimates for the model estimating how white matter volume of the regularized regions predict the cognitive factor*

| Path | Estimate | SE | *p* | Standardized Estimate |
| --- | --- | --- | --- | --- |
| Cognitive_factor    ~    forcepsminor_wmv | -0.007 | 0.019 | 0.696 | -0.010 |
| Cognitive_factor    ~    cingulatecingulum_wmv | -0.080 | 0.015 | *p<0.001* | -0.108 |
| Cognitive_factor    ~    parahippocampalcingulum_wmv | 0.054 | 0.011 | *p<0.001* | 0.073 |
| Cognitive_factor    ~    corticospinalpyramidal_wmv | 0.153 | 0.017 | *p<0.001* | 0.210 |
| Cognitive_factor    ~    anteriorthalamicradiations_wmv | -0.023 | 0.022 | 0.288 | -0.032 |
| Cognitive_factor    ~    uncinate_wmv | -0.011 | 0.015 | 0.470 | -0.015 |
| Cognitive_factor    ~    inferiorlongitudinalfasiculus_wmv | 0.038 | 0.016 | 0.018 | 0.051 |
| Cognitive_factor    ~    inferiorfrontooccipitalfasiculus_wmv | 0.005 | 0.024 | 0.821 | 0.007 |
| Cognitive_factor    ~    superiorlongitudinalfasiculus_wmv | 0.151 | 0.036 | *p<0.001* | 0.205 |
| Cognitive_factor    ~    parietalsuperiorlongitudinalfasiculus_wmv | -0.052 | 0.035 | 0.134 | -0.071 |

*Supplementary Table 35. Comparison of the model with free parameters and the model with parameters constrained to a same value for the white matter volume model with the regularized regions. AIC/BIC are two information criterions. Pr(>Chisq): p-value of the chi-square ratio test.*

|  | Degree of freedom | AIC | BIC | Difference in degree of freedom | Pr (>Chisq) |
| --- | --- | --- | --- | --- | --- |
| Free model | 45 | 123411.4 | 123590.3 | NA | NA |
| Constrained model | 54 | 123560.3 | 123674.8 | 9 | *p<0.001* |

**Models per tissue estimating cognitive factor from several regions in three metrics** 

For grey and white matter, we reported the tables for the models with all the regions included as predictors and the models with only the regions that survived the regularization (the one that we used for the analyses).

**Grey matter metrics (model with all the regions)**

*Supplementary Table 36. Regression estimates for the model estimating how cortical thickness, surface area and grey matter volume of all the regions predict the cognitive factor*

| Path | Estimate | SE | *p* | Standardized Estimate |
| --- | --- | --- | --- | --- |
| Cognitive_factor    ~    bankssts_ct | -0.002 | 0.018 | 0.911 | -0.002 |
| Cognitive_factor    ~    caudalanteriorcingulate_ct | -0.045 | 0.019 | 0.015 | -0.052 |
| Cognitive_factor    ~    caudalmiddlefrontal_ct | 0.067 | 0.029 | 0.022 | 0.085 |
| Cognitive_factor    ~    cuneus_ct | 0.010 | 0.030 | 0.753 | 0.012 |
| Cognitive_factor    ~    entorhinal_ct | 0.021 | 0.019 | 0.273 | 0.025 |
| Cognitive_factor    ~    fusiform_ct | 0.009 | 0.025 | 0.723 | 0.011 |
| Cognitive_factor    ~    inferiorparietal_ct | -0.037 | 0.032 | 0.249 | -0.047 |
| Cognitive_factor    ~    inferiortemporal_ct | 0.060 | 0.029 | 0.039 | 0.076 |
| Cognitive_factor    ~    isthmuscingulate_ct | 0.016 | 0.019 | 0.404 | 0.019 |
| Cognitive_factor    ~    lateraloccipital_ct | 0.025 | 0.037 | 0.500 | 0.033 |
| Cognitive_factor    ~    lateralorbitofrontal_ct | -0.079 | 0.022 | *p<0.001* | -0.098 |
| Cognitive_factor    ~    lingual_ct | 0.063 | 0.032 | 0.046 | 0.081 |
| Cognitive_factor    ~    medialorbitofrontal_ct | 0.054 | 0.023 | 0.020 | 0.066 |
| Cognitive_factor    ~    middletemporal_ct | -0.059 | 0.034 | 0.080 | -0.076 |
| Cognitive_factor    ~    parahippocampal_ct | 0.037 | 0.028 | 0.184 | 0.047 |
| Cognitive_factor    ~    paracentral_ct | 0.011 | 0.027 | 0.697 | 0.013 |
| Cognitive_factor    ~    parsopercularis_ct | -0.044 | 0.027 | 0.100 | -0.053 |
| Cognitive_factor    ~    parsorbitalis_ct | 0.014 | 0.023 | 0.544 | 0.017 |
| Cognitive_factor    ~    parstriangularis_ct | -0.043 | 0.027 | 0.117 | -0.051 |
| Cognitive_factor    ~    pericalcarine_ct | 0.100 | 0.029 | 0.001 | 0.128 |
| Cognitive_factor    ~    postcentral_ct | 0.048 | 0.036 | 0.181 | 0.063 |
| Cognitive_factor    ~    posteriorcingulate_ct | -0.048 | 0.022 | 0.028 | -0.057 |
| Cognitive_factor    ~    precentral_ct | 0.173 | 0.038 | *p<0.001* | 0.223 |
| Cognitive_factor    ~    precuneus_ct | -0.064 | 0.026 | 0.013 | -0.083 |
| Cognitive_factor    ~    rostralanteriorcingulate_ct | -0.053 | 0.020 | 0.006 | -0.061 |
| Cognitive_factor    ~    rostralmiddlefrontal_ct | 0.114 | 0.030 | *p<0.001* | 0.146 |
| Cognitive_factor    ~    superiorfrontal_ct | -0.004 | 0.032 | 0.908 | -0.005 |
| Cognitive_factor    ~    superiorparietal_ct | -0.098 | 0.034 | 0.004 | -0.130 |
| Cognitive_factor    ~    superiortemporal_ct | -0.074 | 0.035 | 0.035 | -0.095 |
| Cognitive_factor    ~    supramarginal_ct | 0.051 | 0.032 | 0.106 | 0.065 |
| Cognitive_factor    ~    frontalpole_ct | -0.001 | 0.022 | 0.979 | -0.001 |
| Cognitive_factor    ~    temporalpole_ct | -0.042 | 0.021 | 0.048 | -0.051 |
| Cognitive_factor    ~    transversetemporal_ct | -0.056 | 0.019 | 0.003 | -0.067 |
| Cognitive_factor    ~    insula_ct | -0.047 | 0.027 | 0.079 | -0.058 |
| Cognitive_factor    ~    bankssts_sa | -0.060 | 0.046 | 0.189 | -0.072 |
| Cognitive_factor    ~    caudalanteriorcingulate_sa | -0.025 | 0.054 | 0.642 | -0.028 |
| Cognitive_factor    ~    caudalmiddlefrontal_sa | 0.219 | 0.070 | 0.002 | 0.275 |
| Cognitive_factor    ~    cuneus_sa | -0.034 | 0.048 | 0.475 | -0.045 |
| Cognitive_factor    ~    entorhinal_sa | 0.022 | 0.032 | 0.493 | 0.027 |
| Cognitive_factor    ~    fusiform_sa | 0.101 | 0.056 | 0.069 | 0.131 |
| Cognitive_factor    ~    inferiorparietal_sa | 0.002 | 0.069 | 0.973 | 0.003 |
| Cognitive_factor    ~    inferiortemporal_sa | 0.289 | 0.065 | *p<0.001* | 0.376 |
| Cognitive_factor    ~    isthmuscingulate_sa | 0.007 | 0.050 | 0.895 | 0.008 |
| Cognitive_factor    ~    lateraloccipital_sa | -0.097 | 0.066 | 0.144 | -0.126 |
| Cognitive_factor    ~    lateralorbitofrontal_sa | -0.137 | 0.054 | 0.012 | -0.180 |
| Cognitive_factor    ~    lingual_sa | 0.115 | 0.058 | 0.048 | 0.152 |
| Cognitive_factor    ~    medialorbitofrontal_sa | 0.221 | 0.049 | *p<0.001* | 0.283 |
| Cognitive_factor    ~    middletemporal_sa | -0.225 | 0.062 | *p<0.001* | -0.298 |
| Cognitive_factor    ~    parahippocampal_sa | -0.062 | 0.038 | 0.106 | -0.077 |
| Cognitive_factor    ~    paracentral_sa | 0.072 | 0.053 | 0.176 | 0.090 |
| Cognitive_factor    ~    parsopercularis_sa | 0.150 | 0.071 | 0.034 | 0.183 |
| Cognitive_factor    ~    parsorbitalis_sa | -0.020 | 0.045 | 0.652 | -0.026 |
| Cognitive_factor    ~    parstriangularis_sa | -0.037 | 0.065 | 0.567 | -0.046 |
| Cognitive_factor    ~    pericalcarine_sa | 0.215 | 0.050 | *p<0.001* | 0.288 |
| Cognitive_factor    ~    postcentral_sa | -0.032 | 0.068 | 0.639 | -0.041 |
| Cognitive_factor    ~    posteriorcingulate_sa | -0.112 | 0.062 | 0.072 | -0.136 |
| Cognitive_factor    ~    precentral_sa | 0.143 | 0.068 | 0.035 | 0.185 |
| Cognitive_factor    ~    precuneus_sa | -0.078 | 0.066 | 0.233 | -0.104 |
| Cognitive_factor    ~    rostralanteriorcingulate_sa | -0.129 | 0.049 | 0.008 | -0.158 |
| Cognitive_factor    ~    rostralmiddlefrontal_sa | 0.307 | 0.065 | *p<0.001* | 0.401 |
| Cognitive_factor    ~    superiorfrontal_sa | -0.043 | 0.077 | 0.577 | -0.057 |
| Cognitive_factor    ~    superiorparietal_sa | -0.027 | 0.067 | 0.685 | -0.035 |
| Cognitive_factor    ~    superiortemporal_sa | -0.107 | 0.065 | 0.098 | -0.141 |
| Cognitive_factor    ~    supramarginal_sa | 0.028 | 0.072 | 0.694 | 0.036 |
| Cognitive_factor    ~    frontalpole_sa | 0.031 | 0.027 | 0.263 | 0.037 |
| Cognitive_factor    ~    temporalpole_sa | -0.128 | 0.027 | *p<0.001* | -0.159 |
| Cognitive_factor    ~    transversetemporal_sa | -0.155 | 0.034 | *p<0.001* | -0.193 |
| Cognitive_factor    ~    insula_sa | -0.090 | 0.054 | 0.096 | -0.117 |
| Cognitive_factor    ~    bankssts_gmv | 0.028 | 0.047 | 0.551 | 0.033 |
| Cognitive_factor    ~    caudalanteriorcingulate_gmv | 0.068 | 0.055 | 0.218 | 0.072 |
| Cognitive_factor    ~    caudalmiddlefrontal_gmv | -0.194 | 0.067 | 0.004 | -0.240 |
| Cognitive_factor    ~    cuneus_gmv | 0.016 | 0.057 | 0.775 | 0.021 |
| Cognitive_factor    ~    entorhinal_gmv | 0.014 | 0.029 | 0.637 | 0.017 |
| Cognitive_factor    ~    fusiform_gmv | -0.047 | 0.054 | 0.383 | -0.060 |
| Cognitive_factor    ~    inferiorparietal_gmv | -0.004 | 0.067 | 0.957 | -0.005 |
| Cognitive_factor    ~    inferiortemporal_gmv | -0.187 | 0.062 | 0.003 | -0.240 |
| Cognitive_factor    ~    isthmuscingulate_gmv | -0.057 | 0.049 | 0.246 | -0.071 |
| Cognitive_factor    ~    lateraloccipital_gmv | 0.116 | 0.071 | 0.102 | 0.151 |
| Cognitive_factor    ~    lateralorbitofrontal_gmv | 0.158 | 0.051 | 0.002 | 0.210 |
| Cognitive_factor    ~    lingual_gmv | -0.131 | 0.065 | 0.042 | -0.171 |
| Cognitive_factor    ~    medialorbitofrontal_gmv | -0.243 | 0.050 | *p<0.001* | -0.306 |
| Cognitive_factor    ~    middletemporal_gmv | 0.271 | 0.056 | *p<0.001* | 0.356 |
| Cognitive_factor    ~    parahippocampal_gmv | 0.087 | 0.042 | 0.038 | 0.107 |
| Cognitive_factor    ~    paracentral_gmv | -0.093 | 0.054 | 0.084 | -0.114 |
| Cognitive_factor    ~    parsopercularis_gmv | -0.117 | 0.067 | 0.080 | -0.142 |
| Cognitive_factor    ~    parsorbitalis_gmv | 0.032 | 0.042 | 0.445 | 0.040 |
| Cognitive_factor    ~    parstriangularis_gmv | 0.004 | 0.062 | 0.945 | 0.005 |
| Cognitive_factor    ~    pericalcarine_gmv | -0.243 | 0.056 | *p<0.001* | -0.324 |
| Cognitive_factor    ~    postcentral_gmv | -0.033 | 0.069 | 0.635 | -0.042 |
| Cognitive_factor    ~    posteriorcingulate_gmv | 0.084 | 0.060 | 0.162 | 0.100 |
| Cognitive_factor    ~    precentral_gmv | -0.095 | 0.066 | 0.150 | -0.123 |
| Cognitive_factor    ~    precuneus_gmv | 0.064 | 0.064 | 0.319 | 0.085 |
| Cognitive_factor    ~    rostralanteriorcingulate_gmv | 0.119 | 0.048 | 0.013 | 0.140 |
| Cognitive_factor    ~    rostralmiddlefrontal_gmv | -0.290 | 0.063 | *p<0.001* | -0.376 |
| Cognitive_factor    ~    superiorfrontal_gmv | 0.081 | 0.068 | 0.234 | 0.106 |
| Cognitive_factor    ~    superiorparietal_gmv | 0.062 | 0.067 | 0.355 | 0.080 |
| Cognitive_factor    ~    superiortemporal_gmv | 0.155 | 0.061 | 0.012 | 0.201 |
| Cognitive_factor    ~    supramarginal_gmv | -0.046 | 0.068 | 0.493 | -0.058 |
| Cognitive_factor    ~    frontalpole_gmv | -0.001 | 0.029 | 0.970 | -0.001 |
| Cognitive_factor    ~    temporalpole_gmv | 0.105 | 0.029 | *p<0.001* | 0.128 |
| Cognitive_factor    ~    transversetemporal_gmv | 0.143 | 0.032 | *p<0.001* | 0.177 |
| Cognitive_factor    ~    insula_gmv | 0.106 | 0.054 | 0.048 | 0.142 |

*Supplementary Figure 21. P-value of the models estimating how the grey matter metrics of each region of interest together predict the cognitive factor* 

*Supplementary Figure 22. Standardized parameter estimates of how the grey matter metrics of each region of interest together predict the cognitive factor* 

*Supplementary Table 37. Comparison of the model with free parameters and the model with parameters constrained to a same value for the grey matter model with all the regions. AIC/BIC are two information criterions. Pr(>Chisq): p-value of the chi-square ratio test.*

|  | Degree of freedom | AIC | BIC | Difference in degree of freedom | Pr (>Chisq) |
| --- | --- | --- | --- | --- | --- |
| Free model | 413 | 129985.5 | 130829.1 | NA | NA |
| Constrained model | 514 | 130728.2 | 130843.5 | 101 | *p<0.001* |

**Grey matter metrics (model with the regularized regions)**

*Supplementary Table 38. Regression estimates for the model estimating how cortical thickness, surface area and grey matter volume of the regularized regions predict the cognitive factor*

| Path | Estimate | SE | *p* | Standardized Estimate |
| --- | --- | --- | --- | --- |
| Cognitive_factor    ~    caudalanteriorcingulate_ct | -0.051 | 0.010 | *p<0.001* | -0.060 |
| Cognitive_factor    ~    caudalmiddlefrontal_ct | 0.040 | 0.015 | 0.008 | 0.051 |
| Cognitive_factor    ~    entorhinal_ct | 0.019 | 0.011 | 0.083 | 0.024 |
| Cognitive_factor    ~    fusiform_ct | -0.002 | 0.014 | 0.889 | -0.003 |
| Cognitive_factor    ~    inferiorparietal_ct | -0.011 | 0.017 | 0.535 | -0.014 |
| Cognitive_factor    ~    lateraloccipital_ct | 0.103 | 0.015 | *p<0.001* | 0.138 |
| Cognitive_factor    ~    lateralorbitofrontal_ct | -0.021 | 0.012 | 0.081 | -0.027 |
| Cognitive_factor    ~    lingual_ct | 0.005 | 0.013 | 0.688 | 0.007 |
| Cognitive_factor    ~    middletemporal_ct | 0.091 | 0.014 | *p<0.001* | 0.117 |
| Cognitive_factor    ~    parahippocampal_ct | 0.082 | 0.012 | *p<0.001* | 0.105 |
| Cognitive_factor    ~    parsopercularis_ct | -0.081 | 0.013 | *p<0.001* | -0.099 |
| Cognitive_factor    ~    pericalcarine_ct | 0.004 | 0.012 | 0.716 | 0.006 |
| Cognitive_factor    ~    superiorfrontal_ct | 0.064 | 0.025 | 0.010 | 0.085 |
| Cognitive_factor    ~    superiorparietal_ct | -0.054 | 0.016 | 0.001 | -0.072 |
| Cognitive_factor    ~    frontalpole_ct | -0.023 | 0.011 | 0.041 | -0.027 |
| Cognitive_factor    ~    temporalpole_ct | 0.033 | 0.014 | 0.017 | 0.040 |
| Cognitive_factor    ~    insula_ct | -0.009 | 0.011 | 0.433 | -0.011 |
| Cognitive_factor    ~    caudalmiddlefrontal_sa | 0.027 | 0.014 | 0.053 | 0.035 |
| Cognitive_factor    ~    entorhinal_sa | 0.037 | 0.012 | 0.002 | 0.047 |
| Cognitive_factor    ~    inferiorparietal_sa | -0.011 | 0.015 | 0.443 | -0.014 |
| Cognitive_factor    ~    isthmuscingulate_sa | -0.050 | 0.013 | *p<0.001* | -0.063 |
| Cognitive_factor    ~    lateraloccipital_sa | 0.040 | 0.014 | 0.003 | 0.052 |
| Cognitive_factor    ~    medialorbitofrontal_sa | 0.022 | 0.014 | 0.120 | 0.029 |
| Cognitive_factor    ~    middletemporal_sa | 0.121 | 0.017 | *p<0.001* | 0.162 |
| Cognitive_factor    ~    paracentral_sa | -0.063 | 0.028 | 0.024 | -0.079 |
| Cognitive_factor    ~    postcentral_sa | -0.040 | 0.016 | 0.013 | -0.053 |
| Cognitive_factor    ~    precentral_sa | 0.054 | 0.017 | 0.001 | 0.071 |
| Cognitive_factor    ~    precuneus_sa | 0.062 | 0.037 | 0.091 | 0.084 |
| Cognitive_factor    ~    superiorfrontal_sa | 0.152 | 0.052 | 0.004 | 0.203 |
| Cognitive_factor    ~    superiorparietal_sa | 0.048 | 0.015 | 0.001 | 0.063 |
| Cognitive_factor    ~    superiortemporal_sa | 0.012 | 0.020 | 0.536 | 0.016 |
| Cognitive_factor    ~    transversetemporal_sa | -0.009 | 0.015 | 0.563 | -0.011 |
| Cognitive_factor    ~    insula_sa | 0.025 | 0.015 | 0.089 | 0.033 |
| Cognitive_factor    ~    bankssts_gmv | -0.030 | 0.015 | 0.040 | -0.036 |
| Cognitive_factor    ~    cuneus_gmv | -0.011 | 0.012 | 0.363 | -0.014 |
| Cognitive_factor    ~    parahippocampal_gmv | 0.028 | 0.012 | 0.022 | 0.035 |
| Cognitive_factor    ~    paracentral_gmv | 0.043 | 0.027 | 0.118 | 0.053 |
| Cognitive_factor    ~    parstriangularis_gmv | -0.017 | 0.012 | 0.140 | -0.021 |
| Cognitive_factor    ~    precuneus_gmv | -0.068 | 0.037 | 0.068 | -0.091 |
| Cognitive_factor    ~    rostralmiddlefrontal_gmv | 0.010 | 0.014 | 0.466 | 0.013 |
| Cognitive_factor    ~    superiorfrontal_gmv | -0.110 | 0.046 | 0.017 | -0.146 |
| Cognitive_factor    ~    temporalpole_gmv | -0.001 | 0.014 | 0.920 | -0.002 |

*Supplementary Figure 23. P-value of the models estimating how the grey matter metrics of each regularized region of interest together predict the cognitive factor* 

*Supplementary Figure 24. Standardized parameter estimates of how the grey matter metrics of each regularized region of interest together predict the cognitive factor* 

*Supplementary Table 39. Comparison of the model with free parameters and the model with parameters constrained to a same value for the grey matter model with the regularized regions. AIC/BIC are two information criterions. Pr(>Chisq): p-value of the chi-square ratio test.*

|  | Degree of freedom | AIC | BIC | Difference in degree of freedom | Pr (>Chisq) |
| --- | --- | --- | --- | --- | --- |
| Free model | 173 | 130312.9 | 130723.9 | NA | NA |
| Constrained model | 214 | 130750.2 | 130865.6 | 41 | *p<0.001* |

**White matter metrics (model with all the regions)**

*Supplementary Table 40. Regression estimates for the model estimating how fractional anisotropy, mean diffusivity and white matter volume of all the regions predict the cognitive factor*

| Path | Estimate | SE | *p* | Standardized Estimate |
| --- | --- | --- | --- | --- |
| Cognitive_factor    ~    fornix_fa | 0.023 | 0.019 | 0.216 | 0.030 |
| Cognitive_factor    ~    cingulatecingulum_fa | -0.095 | 0.015 | *p<0.001* | -0.121 |
| Cognitive_factor    ~    parahippocampalcingulum_fa | 0.013 | 0.013 | 0.312 | 0.017 |
| Cognitive_factor    ~    corticospinalpyramidal_fa | 0.061 | 0.019 | 0.002 | 0.080 |
| Cognitive_factor    ~    anteriorthalamicradiations_fa | 0.038 | 0.017 | 0.021 | 0.050 |
| Cognitive_factor    ~    uncinate_fa | -0.071 | 0.020 | *p<0.001* | -0.092 |
| Cognitive_factor    ~    inferiorlongitudinalfasiculus_fa | 0.018 | 0.019 | 0.332 | 0.023 |
| Cognitive_factor    ~    inferiorfrontooccipitalfasiculus_fa | 0.079 | 0.023 | 0.001 | 0.103 |
| Cognitive_factor    ~    forcepsmajor_fa | 0.004 | 0.022 | 0.858 | 0.006 |
| Cognitive_factor    ~    forcepsminor_fa | -0.056 | 0.025 | 0.023 | -0.078 |
| Cognitive_factor    ~    corpuscallosum_fa | -0.044 | 0.038 | 0.246 | -0.061 |
| Cognitive_factor    ~    superiorlongitudinalfasiculus_fa | -0.171 | 0.143 | 0.230 | -0.225 |
| Cognitive_factor    ~    temporalsuperiorlongitudinalfasiculus_fa | 0.079 | 0.077 | 0.299 | 0.104 |
| Cognitive_factor    ~    parietalsuperiorlongitudinalfasiculus_fa | 0.230 | 0.085 | 0.007 | 0.301 |
| Cognitive_factor    ~    superiorcorticostriate_fa | 0.542 | 0.139 | *p<0.001* | 0.700 |
| Cognitive_factor    ~    superiorcorticostriatefrontalcortex_fa | -0.146 | 0.064 | 0.023 | -0.189 |
| Cognitive_factor    ~    superiorcorticostriateparietalcortex_fa | -0.448 | 0.093 | *p<0.001* | -0.573 |
| Cognitive_factor    ~    striatalinferiorfrontalcortex_fa | -0.054 | 0.018 | 0.002 | -0.068 |
| Cognitive_factor    ~    inferiorfrontalsuperiorfrontalcortex_fa | 0.064 | 0.022 | 0.004 | 0.083 |
| Cognitive_factor    ~    fornix_exfimbria_fa | -0.047 | 0.018 | 0.009 | -0.059 |
| Cognitive_factor    ~    fornix_md | -0.005 | 0.026 | 0.844 | -0.007 |
| Cognitive_factor    ~    cingulatecingulum_md | 0.015 | 0.017 | 0.354 | 0.018 |
| Cognitive_factor    ~    parahippocampalcingulum_md | -0.012 | 0.015 | 0.445 | -0.015 |
| Cognitive_factor    ~    corticospinalpyramidal_md | 0.111 | 0.019 | *p<0.001* | 0.145 |
| Cognitive_factor    ~    anteriorthalamicradiations_md | 0.065 | 0.017 | *p<0.001* | 0.085 |
| Cognitive_factor    ~    uncinate_md | -0.068 | 0.022 | 0.002 | -0.089 |
| Cognitive_factor    ~    inferiorlongitudinalfasiculus_md | -0.004 | 0.023 | 0.852 | -0.005 |
| Cognitive_factor    ~    inferiorfrontooccipitalfasiculus_md | 0.065 | 0.027 | 0.018 | 0.081 |
| Cognitive_factor    ~    forcepsmajor_md | 0.023 | 0.023 | 0.328 | 0.032 |
| Cognitive_factor    ~    forcepsminor_md | 0.060 | 0.023 | 0.008 | 0.084 |
| Cognitive_factor    ~    corpuscallosum_md | -0.078 | 0.032 | 0.013 | -0.107 |
| Cognitive_factor    ~    superiorlongitudinalfasiculus_md | -0.116 | 0.202 | 0.564 | -0.151 |
| Cognitive_factor    ~    temporalsuperiorlongitudinalfasiculus_md | 0.132 | 0.106 | 0.212 | 0.171 |
| Cognitive_factor    ~    parietalsuperiorlongitudinalfasiculus_md | -0.062 | 0.116 | 0.592 | -0.080 |
| Cognitive_factor    ~    superiorcorticostriate_md | -0.009 | 0.164 | 0.957 | -0.011 |
| Cognitive_factor    ~    superiorcorticostriatefrontalcortex_md | -0.040 | 0.074 | 0.583 | -0.051 |
| Cognitive_factor    ~    superiorcorticostriateparietalcortex_md | -0.106 | 0.114 | 0.353 | -0.134 |
| Cognitive_factor    ~    striatalinferiorfrontalcortex_md | -0.061 | 0.027 | 0.023 | -0.074 |
| Cognitive_factor    ~    inferiorfrontalsuperiorfrontalcortex_md | 0.101 | 0.032 | 0.002 | 0.131 |
| Cognitive_factor    ~    fornix_exfimbria_md | 0.022 | 0.025 | 0.376 | 0.029 |
| Cognitive_factor    ~    fornix_wmv | 0.237 | 0.032 | 0.000 | 0.322 |
| Cognitive_factor    ~    cingulatecingulum_wmv | -0.021 | 0.019 | 0.271 | -0.028 |
| Cognitive_factor    ~    parahippocampalcingulum_wmv | 0.039 | 0.013 | 0.004 | 0.051 |
| Cognitive_factor    ~    corticospinalpyramidal_wmv | 0.198 | 0.024 | *p<0.001* | 0.266 |
| Cognitive_factor    ~    anteriorthalamicradiations_wmv | 0.018 | 0.027 | 0.493 | 0.025 |
| Cognitive_factor    ~    uncinate_wmv | -0.007 | 0.018 | 0.698 | -0.009 |
| Cognitive_factor    ~    inferiorlongitudinalfasiculus_wmv | -0.003 | 0.021 | 0.873 | -0.004 |
| Cognitive_factor    ~    inferiorfrontooccipitalfasiculus_wmv | -0.054 | 0.032 | 0.089 | -0.073 |
| Cognitive_factor    ~    forcepsmajor_wmv | 0.000 | 0.021 | 0.996 | 0.000 |
| Cognitive_factor    ~    forcepsminor_wmv | -0.046 | 0.026 | 0.084 | -0.064 |
| Cognitive_factor    ~    corpuscallosum_wmv | 0.082 | 0.044 | 0.060 | 0.114 |
| Cognitive_factor    ~    superiorlongitudinalfasiculus_wmv | -0.111 | 0.084 | 0.187 | -0.147 |
| Cognitive_factor    ~    temporalsuperiorlongitudinalfasiculus_wmv | 0.147 | 0.056 | 0.009 | 0.193 |
| Cognitive_factor    ~    parietalsuperiorlongitudinalfasiculus_wmv | 0.009 | 0.047 | 0.849 | 0.012 |
| Cognitive_factor    ~    superiorcorticostriate_wmv | -0.085 | 0.087 | 0.331 | -0.114 |
| Cognitive_factor    ~    superiorcorticostriatefrontalcortex_wmv | -0.158 | 0.053 | 0.003 | -0.212 |
| Cognitive_factor    ~    superiorcorticostriateparietalcortex_wmv | -0.008 | 0.057 | 0.885 | -0.011 |
| Cognitive_factor    ~    striatalinferiorfrontalcortex_wmv | 0.030 | 0.021 | 0.162 | 0.039 |
| Cognitive_factor    ~    inferiorfrontalsuperiorfrontalcortex_wmv | 0.076 | 0.024 | 0.002 | 0.101 |
| Cognitive_factor    ~    fornix_exfimbria_wmv | -0.065 | 0.031 | 0.036 | -0.084 |

*Supplementary Table 41. Comparison of the model with free parameters and the model with parameters constrained to a same value for the white matter model with all the regions. AIC/BIC are two information criterions. Pr(>Chisq): p-value of the chi-square ratio test.*

|  | Degree of freedom | AIC | BIC | Difference in degree of freedom | Pr (>Chisq) |
| --- | --- | --- | --- | --- | --- |
| Free model | 245 | 122718.7 | 123255.4 | NA | NA |
| Constrained model | 304 | 123613.8 | 123728.3 | 59 | *p<0.001* |

**White matter metrics (model with the regularized regions)**

*Supplementary Table 42. Regression estimates for the model estimating how fractional anisotropy, mean diffusivity and white matter volume of the regularized regions predict the cognitive factor*

| Path | Estimate | SE | *p* | Standardized Estimate |
| --- | --- | --- | --- | --- |
| Cognitive_factor    ~    cingulatecingulum_fa | -0.106 | 0.012 | *p<0.001* | -0.137 |
| Cognitive_factor    ~    parahippocampalcingulum_fa | 0.018 | 0.012 | 0.130 | 0.024 |
| Cognitive_factor    ~    uncinate_fa | -0.089 | 0.019 | *p<0.001* | -0.116 |
| Cognitive_factor    ~    inferiorfrontooccipitalfasiculus_fa | 0.045 | 0.018 | 0.013 | 0.060 |
| Cognitive_factor    ~    temporalsuperiorlongitudinalfasiculus_fa | 0.120 | 0.016 | *p<0.001* | 0.158 |
| Cognitive_factor    ~    inferiorfrontalsuperiorfrontalcortex_fa | 0.068 | 0.016 | *p<0.001* | 0.088 |
| Cognitive_factor    ~    fornix_exfimbria_fa | -0.009 | 0.011 | 0.420 | -0.011 |
| Cognitive_factor    ~    uncinate_md | -0.028 | 0.017 | 0.095 | -0.037 |
| Cognitive_factor    ~    inferiorfrontooccipitalfasiculus_md | -0.007 | 0.021 | 0.740 | -0.009 |
| Cognitive_factor    ~    forcepsmajor_md | 0.028 | 0.014 | 0.046 | 0.039 |
| Cognitive_factor    ~    forcepsminor_md | 0.093 | 0.012 | *p<0.001* | 0.132 |
| Cognitive_factor    ~    striatalinferiorfrontalcortex_md | -0.030 | 0.020 | 0.135 | -0.037 |
| Cognitive_factor    ~    fornix_wmv | 0.185 | 0.015 | *p<0.001* | 0.254 |
| Cognitive_factor    ~    anteriorthalamicradiations_wmv | -0.015 | 0.022 | 0.494 | -0.021 |
| Cognitive_factor    ~    uncinate_wmv | 0.006 | 0.017 | 0.728 | 0.008 |
| Cognitive_factor    ~    inferiorlongitudinalfasiculus_wmv | 0.009 | 0.017 | 0.596 | 0.012 |
| Cognitive_factor    ~    inferiorfrontooccipitalfasiculus_wmv | 0.010 | 0.027 | 0.707 | 0.014 |
| Cognitive_factor    ~    forcepsmajor_wmv | 0.006 | 0.019 | 0.746 | 0.008 |
| Cognitive_factor    ~    forcepsminor_wmv | -0.039 | 0.024 | 0.107 | -0.055 |
| Cognitive_factor    ~    corpuscallosum_wmv | 0.055 | 0.036 | 0.131 | 0.077 |
| Cognitive_factor    ~    superiorlongitudinalfasiculus_wmv | 0.046 | 0.037 | 0.214 | 0.062 |
| Cognitive_factor    ~    parietalsuperiorlongitudinalfasiculus_wmv | -0.022 | 0.035 | 0.531 | -0.030 |
| Cognitive_factor    ~    inferiorfrontalsuperiorfrontalcortex_wmv | 0.025 | 0.020 | 0.199 | 0.034 |

*Supplementary Table 43. Standardized parameter estimates of how the white matter metrics of each regularized region of interest together predict the cognitive factor*

| Path | FA | MD | WMV |
| --- | --- | --- | --- |
| Cognitive_factor    ~    anteriorthalamicradiations | - | - | -0.021 |
| Cognitive_factor    ~    cingulatecingulum | -0.137 | - | - |
| Cognitive_factor    ~    corpuscallosum | - | - | 0.077 |
| Cognitive_factor    ~    forcepsmajor | - | 0.039 | 0.008 |
| Cognitive_factor    ~    forcepsminor | - | 0.132 | -0.055 |
| Cognitive_factor    ~    fornix | - | - | 0.254 |
| Cognitive_factor    ~    fornix_exfimbria | -0.011 | - | - |
| Cognitive_factor    ~    inferiorfrontalsuperiorfrontalcortex | 0.088 | - | 0.034 |
| Cognitive_factor    ~    inferiorfrontooccipitalfasiculus | 0.060 | -0.009 | 0.014 |
| Cognitive_factor    ~    inferiorlongitudinalfasiculus | - | - | 0.012 |
| Cognitive_factor    ~    parahippocampalcingulum | 0.024 | - | - |
| Cognitive_factor    ~    parietalsuperiorlongitudinalfasiculus | - | - | -0.030 |
| Cognitive_factor    ~    striatalinferiorfrontalcortex | - | -0.037 | - |
| Cognitive_factor    ~    superiorlongitudinalfasiculus | - | - | 0.062 |
| Cognitive_factor    ~    temporalsuperiorlongitudinalfasiculus | 0.158 | - | - |
| Cognitive_factor    ~    uncinate | -0.116 | -0.037 | 0.008 |

*Supplementary Table 44. Comparison of the model with free parameters and the model with parameters constrained to a same value for the white matter model with the regularized regions. AIC/BIC are two information criterions. Pr(>Chisq): p-value of the chi-square ratio test.*

|  | Degree of freedom | AIC | BIC | Difference in degree of freedom | Pr (>Chisq) |
| --- | --- | --- | --- | --- | --- |
| Free model | 97 | 123031.8 | 123303.7 | NA | NA |
| Constrained model | 119 | 123497.1 | 123611.6 | 22 | *p<0.001* |

**Models with grey and white matter metrics estimating cognitive factor from several regions in the six metrics** 

For the grey and white matter model, we reported the table for the model with all the regions and the model with only the regions that survived the regularization included as predictors (the one that we used for the analyses).

**Grey and white matter metrics (model with all the regions)**

*Supplementary Table 45. Regression estimates for the model estimating how cortical thickness, surface area, grey matter volume, fractional anisotropy, mean diffusivity and white matter volume of all the regions predict the cognitive factor*

| Path | Estimate | SE | *p* | Standardized Estimate |
| --- | --- | --- | --- | --- |
| Cognitive_factor    ~    bankssts_ct | 0.002 | 0.018 | 0.916 | 0.002 |
| Cognitive_factor    ~    caudalanteriorcingulate_ct | -0.027 | 0.018 | 0.136 | -0.032 |
| Cognitive_factor    ~    caudalmiddlefrontal_ct | 0.015 | 0.030 | 0.622 | 0.018 |
| Cognitive_factor    ~    cuneus_ct | 0.007 | 0.031 | 0.812 | 0.009 |
| Cognitive_factor    ~    entorhinal_ct | 0.014 | 0.020 | 0.485 | 0.016 |
| Cognitive_factor    ~    fusiform_ct | -0.003 | 0.026 | 0.920 | -0.003 |
| Cognitive_factor    ~    inferiorparietal_ct | -0.022 | 0.032 | 0.486 | -0.028 |
| Cognitive_factor    ~    inferiortemporal_ct | 0.042 | 0.030 | 0.152 | 0.052 |
| Cognitive_factor    ~    isthmuscingulate_ct | 0.022 | 0.020 | 0.283 | 0.026 |
| Cognitive_factor    ~    lateraloccipital_ct | -0.007 | 0.038 | 0.845 | -0.010 |
| Cognitive_factor    ~    lateralorbitofrontal_ct | -0.076 | 0.023 | 0.001 | -0.092 |
| Cognitive_factor    ~    lingual_ct | 0.061 | 0.032 | 0.055 | 0.078 |
| Cognitive_factor    ~    medialorbitofrontal_ct | 0.040 | 0.024 | 0.097 | 0.047 |
| Cognitive_factor    ~    middletemporal_ct | -0.033 | 0.034 | 0.335 | -0.041 |
| Cognitive_factor    ~    parahippocampal_ct | 0.028 | 0.029 | 0.331 | 0.035 |
| Cognitive_factor    ~    paracentral_ct | 0.015 | 0.028 | 0.581 | 0.019 |
| Cognitive_factor    ~    parsopercularis_ct | -0.038 | 0.027 | 0.163 | -0.045 |
| Cognitive_factor    ~    parsorbitalis_ct | 0.006 | 0.024 | 0.806 | 0.007 |
| Cognitive_factor    ~    parstriangularis_ct | -0.050 | 0.027 | 0.068 | -0.059 |
| Cognitive_factor    ~    pericalcarine_ct | 0.049 | 0.030 | 0.096 | 0.063 |
| Cognitive_factor    ~    postcentral_ct | 0.013 | 0.037 | 0.735 | 0.016 |
| Cognitive_factor    ~    posteriorcingulate_ct | -0.029 | 0.023 | 0.206 | -0.034 |
| Cognitive_factor    ~    precentral_ct | 0.166 | 0.039 | *p<0.001* | 0.208 |
| Cognitive_factor    ~    precuneus_ct | -0.053 | 0.026 | 0.045 | -0.067 |
| Cognitive_factor    ~    rostralanteriorcingulate_ct | -0.038 | 0.020 | 0.059 | -0.043 |
| Cognitive_factor    ~    rostralmiddlefrontal_ct | 0.115 | 0.031 | *p<0.001* | 0.144 |
| Cognitive_factor    ~    superiorfrontal_ct | -0.007 | 0.033 | 0.827 | -0.009 |
| Cognitive_factor    ~    superiorparietal_ct | -0.061 | 0.036 | 0.084 | -0.080 |
| Cognitive_factor    ~    superiortemporal_ct | -0.072 | 0.036 | 0.043 | -0.091 |
| Cognitive_factor    ~    supramarginal_ct | 0.052 | 0.032 | 0.101 | 0.065 |
| Cognitive_factor    ~    frontalpole_ct | 0.004 | 0.022 | 0.868 | 0.004 |
| Cognitive_factor    ~    temporalpole_ct | -0.038 | 0.022 | 0.077 | -0.045 |
| Cognitive_factor    ~    transversetemporal_ct | -0.043 | 0.020 | 0.027 | -0.052 |
| Cognitive_factor    ~    insula_ct | -0.023 | 0.028 | 0.399 | -0.028 |
| Cognitive_factor    ~    bankssts_sa | -0.059 | 0.046 | 0.202 | -0.070 |
| Cognitive_factor    ~    caudalanteriorcingulate_sa | 0.018 | 0.048 | 0.703 | 0.020 |
| Cognitive_factor    ~    caudalmiddlefrontal_sa | 0.069 | 0.073 | 0.343 | 0.086 |
| Cognitive_factor    ~    cuneus_sa | -0.035 | 0.049 | 0.472 | -0.046 |
| Cognitive_factor    ~    entorhinal_sa | 0.009 | 0.033 | 0.784 | 0.011 |
| Cognitive_factor    ~    fusiform_sa | 0.061 | 0.057 | 0.289 | 0.079 |
| Cognitive_factor    ~    inferiorparietal_sa | 0.056 | 0.070 | 0.421 | 0.072 |
| Cognitive_factor    ~    inferiortemporal_sa | 0.231 | 0.065 | *p<0.001* | 0.299 |
| Cognitive_factor    ~    isthmuscingulate_sa | -0.001 | 0.053 | 0.978 | -0.002 |
| Cognitive_factor    ~    lateraloccipital_sa | -0.143 | 0.068 | 0.034 | -0.186 |
| Cognitive_factor    ~    lateralorbitofrontal_sa | -0.108 | 0.055 | 0.048 | -0.142 |
| Cognitive_factor    ~    lingual_sa | 0.113 | 0.060 | 0.057 | 0.149 |
| Cognitive_factor    ~    medialorbitofrontal_sa | 0.169 | 0.051 | 0.001 | 0.215 |
| Cognitive_factor    ~    middletemporal_sa | -0.168 | 0.063 | 0.008 | -0.222 |
| Cognitive_factor    ~    parahippocampal_sa | -0.060 | 0.039 | 0.129 | -0.074 |
| Cognitive_factor    ~    paracentral_sa | 0.070 | 0.055 | 0.205 | 0.086 |
| Cognitive_factor    ~    parsopercularis_sa | 0.151 | 0.071 | 0.032 | 0.184 |
| Cognitive_factor    ~    parsorbitalis_sa | -0.019 | 0.046 | 0.683 | -0.024 |
| Cognitive_factor    ~    parstriangularis_sa | -0.084 | 0.067 | 0.212 | -0.103 |
| Cognitive_factor    ~    pericalcarine_sa | 0.123 | 0.052 | 0.018 | 0.164 |
| Cognitive_factor    ~    postcentral_sa | -0.042 | 0.070 | 0.551 | -0.054 |
| Cognitive_factor    ~    posteriorcingulate_sa | -0.097 | 0.073 | 0.184 | -0.116 |
| Cognitive_factor    ~    precentral_sa | 0.149 | 0.070 | 0.032 | 0.192 |
| Cognitive_factor    ~    precuneus_sa | -0.023 | 0.066 | 0.727 | -0.031 |
| Cognitive_factor    ~    rostralanteriorcingulate_sa | -0.118 | 0.050 | 0.018 | -0.144 |
| Cognitive_factor    ~    rostralmiddlefrontal_sa | 0.261 | 0.068 | *p<0.001* | 0.339 |
| Cognitive_factor    ~    superiorfrontal_sa | -0.007 | 0.078 | 0.932 | -0.009 |
| Cognitive_factor    ~    superiorparietal_sa | 0.012 | 0.069 | 0.862 | 0.016 |
| Cognitive_factor    ~    superiortemporal_sa | -0.095 | 0.066 | 0.150 | -0.124 |
| Cognitive_factor    ~    supramarginal_sa | 0.050 | 0.074 | 0.497 | 0.063 |
| Cognitive_factor    ~    frontalpole_sa | 0.022 | 0.028 | 0.432 | 0.026 |
| Cognitive_factor    ~    temporalpole_sa | -0.120 | 0.027 | *p<0.001* | -0.148 |
| Cognitive_factor    ~    transversetemporal_sa | -0.122 | 0.034 | *p<0.001* | -0.151 |
| Cognitive_factor    ~    insula_sa | -0.057 | 0.056 | 0.303 | -0.074 |
| Cognitive_factor    ~    bankssts_gmv | 0.033 | 0.047 | 0.485 | 0.039 |
| Cognitive_factor    ~    caudalanteriorcingulate_gmv | 0.014 | 0.050 | 0.785 | 0.014 |
| Cognitive_factor    ~    caudalmiddlefrontal_gmv | -0.041 | 0.070 | 0.561 | -0.050 |
| Cognitive_factor    ~    cuneus_gmv | 0.021 | 0.058 | 0.721 | 0.027 |
| Cognitive_factor    ~    entorhinal_gmv | 0.009 | 0.030 | 0.755 | 0.012 |
| Cognitive_factor    ~    fusiform_gmv | -0.030 | 0.056 | 0.590 | -0.038 |
| Cognitive_factor    ~    inferiorparietal_gmv | -0.067 | 0.068 | 0.321 | -0.085 |
| Cognitive_factor    ~    inferiortemporal_gmv | -0.160 | 0.063 | 0.011 | -0.204 |
| Cognitive_factor    ~    isthmuscingulate_gmv | -0.055 | 0.052 | 0.290 | -0.068 |
| Cognitive_factor    ~    lateraloccipital_gmv | 0.161 | 0.072 | 0.026 | 0.209 |
| Cognitive_factor    ~    lateralorbitofrontal_gmv | 0.150 | 0.052 | 0.004 | 0.198 |
| Cognitive_factor    ~    lingual_gmv | -0.135 | 0.066 | 0.041 | -0.175 |
| Cognitive_factor    ~    medialorbitofrontal_gmv | -0.161 | 0.052 | 0.002 | -0.201 |
| Cognitive_factor    ~    middletemporal_gmv | 0.196 | 0.057 | 0.001 | 0.257 |
| Cognitive_factor    ~    parahippocampal_gmv | 0.058 | 0.043 | 0.176 | 0.071 |
| Cognitive_factor    ~    paracentral_gmv | -0.077 | 0.055 | 0.164 | -0.093 |
| Cognitive_factor    ~    parsopercularis_gmv | -0.114 | 0.067 | 0.090 | -0.137 |
| Cognitive_factor    ~    parsorbitalis_gmv | 0.038 | 0.043 | 0.372 | 0.047 |
| Cognitive_factor    ~    parstriangularis_gmv | 0.048 | 0.064 | 0.449 | 0.058 |
| Cognitive_factor    ~    pericalcarine_gmv | -0.150 | 0.058 | 0.010 | -0.199 |
| Cognitive_factor    ~    postcentral_gmv | -0.017 | 0.071 | 0.809 | -0.022 |
| Cognitive_factor    ~    posteriorcingulate_gmv | 0.065 | 0.069 | 0.351 | 0.077 |
| Cognitive_factor    ~    precentral_gmv | -0.109 | 0.068 | 0.107 | -0.140 |
| Cognitive_factor    ~    precuneus_gmv | 0.014 | 0.065 | 0.830 | 0.018 |
| Cognitive_factor    ~    rostralanteriorcingulate_gmv | 0.119 | 0.048 | 0.014 | 0.139 |
| Cognitive_factor    ~    rostralmiddlefrontal_gmv | -0.245 | 0.065 | *p<0.001* | -0.315 |
| Cognitive_factor    ~    superiorfrontal_gmv | 0.056 | 0.069 | 0.418 | 0.073 |
| Cognitive_factor    ~    superiorparietal_gmv | 0.011 | 0.069 | 0.873 | 0.014 |
| Cognitive_factor    ~    superiortemporal_gmv | 0.147 | 0.063 | 0.019 | 0.190 |
| Cognitive_factor    ~    supramarginal_gmv | -0.070 | 0.069 | 0.311 | -0.087 |
| Cognitive_factor    ~    frontalpole_gmv | -0.007 | 0.029 | 0.820 | -0.008 |
| Cognitive_factor    ~    temporalpole_gmv | 0.097 | 0.030 | 0.001 | 0.116 |
| Cognitive_factor    ~    transversetemporal_gmv | 0.110 | 0.033 | 0.001 | 0.136 |
| Cognitive_factor    ~    insula_gmv | 0.084 | 0.055 | 0.127 | 0.111 |
| Cognitive_factor    ~    fornix_fa | 0.019 | 0.019 | 0.332 | 0.024 |
| Cognitive_factor    ~    cingulatecingulum_fa | -0.044 | 0.016 | 0.006 | -0.056 |
| Cognitive_factor    ~    parahippocampalcingulum_fa | -0.001 | 0.013 | 0.955 | -0.001 |
| Cognitive_factor    ~    corticospinalpyramidal_fa | 0.048 | 0.020 | 0.015 | 0.062 |
| Cognitive_factor    ~    anteriorthalamicradiations_fa | 0.034 | 0.017 | 0.048 | 0.043 |
| Cognitive_factor    ~    uncinate_fa | -0.056 | 0.020 | 0.005 | -0.071 |
| Cognitive_factor    ~    inferiorlongitudinalfasiculus_fa | 0.015 | 0.019 | 0.413 | 0.019 |
| Cognitive_factor    ~    inferiorfrontooccipitalfasiculus_fa | 0.071 | 0.024 | 0.003 | 0.092 |
| Cognitive_factor    ~    forcepsmajor_fa | 0.020 | 0.023 | 0.375 | 0.028 |
| Cognitive_factor    ~    forcepsminor_fa | -0.026 | 0.024 | 0.289 | -0.036 |
| Cognitive_factor    ~    corpuscallosum_fa | -0.086 | 0.040 | 0.030 | -0.118 |
| Cognitive_factor    ~    superiorlongitudinalfasiculus_fa | -0.076 | 0.142 | 0.594 | -0.099 |
| Cognitive_factor    ~    temporalsuperiorlongitudinalfasiculus_fa | 0.040 | 0.077 | 0.602 | 0.052 |
| Cognitive_factor    ~    parietalsuperiorlongitudinalfasiculus_fa | 0.124 | 0.084 | 0.142 | 0.161 |
| Cognitive_factor    ~    superiorcorticostriate_fa | 0.365 | 0.132 | 0.006 | 0.469 |
| Cognitive_factor    ~    superiorcorticostriatefrontalcortex_fa | -0.088 | 0.059 | 0.132 | -0.114 |
| Cognitive_factor    ~    superiorcorticostriateparietalcortex_fa | -0.320 | 0.091 | *p<0.001* | -0.407 |
| Cognitive_factor    ~    striatalinferiorfrontalcortex_fa | -0.047 | 0.018 | 0.008 | -0.059 |
| Cognitive_factor    ~    inferiorfrontalsuperiorfrontalcortex_fa | 0.055 | 0.022 | 0.013 | 0.071 |
| Cognitive_factor    ~    fornix_exfimbria_fa | -0.050 | 0.018 | 0.005 | -0.062 |
| Cognitive_factor    ~    fornix_md | -0.016 | 0.024 | 0.497 | -0.021 |
| Cognitive_factor    ~    cingulatecingulum_md | 0.007 | 0.016 | 0.654 | 0.008 |
| Cognitive_factor    ~    parahippocampalcingulum_md | -0.026 | 0.015 | 0.085 | -0.034 |
| Cognitive_factor    ~    corticospinalpyramidal_md | 0.096 | 0.019 | *p<0.001* | 0.124 |
| Cognitive_factor    ~    anteriorthalamicradiations_md | 0.041 | 0.017 | 0.016 | 0.054 |
| Cognitive_factor    ~    uncinate_md | -0.042 | 0.021 | 0.043 | -0.055 |
| Cognitive_factor    ~    inferiorlongitudinalfasiculus_md | -0.006 | 0.023 | 0.801 | -0.007 |
| Cognitive_factor    ~    inferiorfrontooccipitalfasiculus_md | 0.031 | 0.027 | 0.247 | 0.039 |
| Cognitive_factor    ~    forcepsmajor_md | 0.060 | 0.021 | 0.004 | 0.083 |
| Cognitive_factor    ~    forcepsminor_md | 0.067 | 0.023 | 0.003 | 0.093 |
| Cognitive_factor    ~    corpuscallosum_md | -0.120 | 0.032 | *p<0.001* | -0.163 |
| Cognitive_factor    ~    superiorlongitudinalfasiculus_md | -0.166 | 0.204 | 0.415 | -0.214 |
| Cognitive_factor    ~    temporalsuperiorlongitudinalfasiculus_md | 0.149 | 0.107 | 0.163 | 0.192 |
| Cognitive_factor    ~    parietalsuperiorlongitudinalfasiculus_md | -0.023 | 0.116 | 0.844 | -0.029 |
| Cognitive_factor    ~    superiorcorticostriate_md | 0.119 | 0.160 | 0.457 | 0.149 |
| Cognitive_factor    ~    superiorcorticostriatefrontalcortex_md | -0.147 | 0.073 | 0.044 | -0.185 |
| Cognitive_factor    ~    superiorcorticostriateparietalcortex_md | -0.123 | 0.111 | 0.266 | -0.155 |
| Cognitive_factor    ~    striatalinferiorfrontalcortex_md | -0.035 | 0.026 | 0.171 | -0.043 |
| Cognitive_factor    ~    inferiorfrontalsuperiorfrontalcortex_md | 0.134 | 0.032 | *p<0.001* | 0.172 |
| Cognitive_factor    ~    fornix_exfimbria_md | 0.022 | 0.024 | 0.365 | 0.029 |
| Cognitive_factor    ~    fornix_wmv | 0.134 | 0.034 | *p<0.001* | 0.182 |
| Cognitive_factor    ~    cingulatecingulum_wmv | -0.006 | 0.021 | 0.776 | -0.008 |
| Cognitive_factor    ~    parahippocampalcingulum_wmv | 0.041 | 0.014 | 0.004 | 0.053 |
| Cognitive_factor    ~    corticospinalpyramidal_wmv | 0.194 | 0.024 | *p<0.001* | 0.260 |
| Cognitive_factor    ~    anteriorthalamicradiations_wmv | 0.028 | 0.028 | 0.306 | 0.038 |
| Cognitive_factor    ~    uncinate_wmv | -0.001 | 0.019 | 0.972 | -0.001 |
| Cognitive_factor    ~    inferiorlongitudinalfasiculus_wmv | -0.010 | 0.022 | 0.662 | -0.013 |
| Cognitive_factor    ~    inferiorfrontooccipitalfasiculus_wmv | -0.073 | 0.032 | 0.025 | -0.097 |
| Cognitive_factor    ~    forcepsmajor_wmv | 0.011 | 0.022 | 0.601 | 0.016 |
| Cognitive_factor    ~    forcepsminor_wmv | -0.108 | 0.028 | *p<0.001* | -0.150 |
| Cognitive_factor    ~    corpuscallosum_wmv | 0.094 | 0.046 | 0.038 | 0.131 |
| Cognitive_factor    ~    superiorlongitudinalfasiculus_wmv | -0.110 | 0.084 | 0.188 | -0.146 |
| Cognitive_factor    ~    temporalsuperiorlongitudinalfasiculus_wmv | 0.125 | 0.056 | 0.025 | 0.164 |
| Cognitive_factor    ~    parietalsuperiorlongitudinalfasiculus_wmv | 0.026 | 0.048 | 0.588 | 0.034 |
| Cognitive_factor    ~    superiorcorticostriate_wmv | 0.052 | 0.087 | 0.551 | 0.069 |
| Cognitive_factor    ~    superiorcorticostriatefrontalcortex_wmv | -0.219 | 0.053 | *p<0.001* | -0.292 |
| Cognitive_factor    ~    superiorcorticostriateparietalcortex_wmv | -0.070 | 0.057 | 0.218 | -0.092 |
| Cognitive_factor    ~    striatalinferiorfrontalcortex_wmv | 0.006 | 0.022 | 0.804 | 0.007 |
| Cognitive_factor    ~    inferiorfrontalsuperiorfrontalcortex_wmv | 0.005 | 0.026 | 0.851 | 0.007 |
| Cognitive_factor    ~    fornix_exfimbria_wmv | -0.005 | 0.032 | 0.880 | -0.006 |

*Supplementary Table 46. Comparison of the model with free parameters and the model with parameters constrained to a same value for the grey & white matter model with all the regions. AIC/BIC are two information criterions. Pr(>Chisq): p-value of the chi-square ratio test.*

|  | Degree of freedom | AIC | BIC | Difference in degree of freedom | Pr (>Chisq) |
| --- | --- | --- | --- | --- | --- |
| Free model | 653 | 122287 | 123554 | NA | NA |
| Constrained model | 814 | 123389 | 123503 | 161 | *p<0.001* |

**Grey and white matter metrics (model with the regularized regions)**

*Supplementary Table 47. Regression estimates for the model estimating how cortical thickness, surface area, grey matter volume, fractional anisotropy, mean diffusivity and white matter volume of the regularized regions predict the cognitive factor*

| Path | Estimate | SE | *p* | Standardized Estimate |
| --- | --- | --- | --- | --- |
| Cognitive_factor    ~    caudalmiddlefrontal_ct | 0.003 | 0.016 | 0.857 | 0.004 |
| Cognitive_factor    ~    fusiform_ct | 0.012 | 0.015 | 0.411 | 0.015 |
| Cognitive_factor    ~    lateralorbitofrontal_ct | -0.009 | 0.013 | 0.507 | -0.011 |
| Cognitive_factor    ~    medialorbitofrontal_ct | -0.037 | 0.012 | 0.003 | -0.044 |
| Cognitive_factor    ~    middletemporal_ct | 0.066 | 0.014 | *p<0.001* | 0.083 |
| Cognitive_factor    ~    parahippocampal_ct | 0.099 | 0.013 | *p<0.001* | 0.127 |
| Cognitive_factor    ~    paracentral_ct | 0.043 | 0.015 | 0.004 | 0.054 |
| Cognitive_factor    ~    parsorbitalis_ct | 0.017 | 0.013 | 0.186 | 0.021 |
| Cognitive_factor    ~    pericalcarine_ct | 0.026 | 0.013 | 0.044 | 0.034 |
| Cognitive_factor    ~    precuneus_ct | -0.055 | 0.014 | *p<0.001* | -0.072 |
| Cognitive_factor    ~    rostralanteriorcingulate_ct | -0.020 | 0.012 | 0.089 | -0.023 |
| Cognitive_factor    ~    rostralmiddlefrontal_ct | -0.017 | 0.017 | 0.317 | -0.021 |
| Cognitive_factor    ~    superiorfrontal_ct | -0.019 | 0.018 | 0.289 | -0.026 |
| Cognitive_factor    ~    superiortemporal_ct | -0.020 | 0.014 | 0.155 | -0.026 |
| Cognitive_factor    ~    insula_ct | 0.001 | 0.012 | 0.926 | 0.001 |
| Cognitive_factor    ~    bankssts_sa | -0.008 | 0.015 | 0.578 | -0.010 |
| Cognitive_factor    ~    cuneus_sa | -0.006 | 0.017 | 0.722 | -0.008 |
| Cognitive_factor    ~    entorhinal_sa | 0.008 | 0.011 | 0.499 | 0.010 |
| Cognitive_factor    ~    lateraloccipital_sa | 0.026 | 0.015 | 0.075 | 0.034 |
| Cognitive_factor    ~    parsorbitalis_sa | 0.041 | 0.014 | 0.003 | 0.053 |
| Cognitive_factor    ~    postcentral_sa | -0.029 | 0.017 | 0.076 | -0.039 |
| Cognitive_factor    ~    precentral_sa | 0.008 | 0.028 | 0.760 | 0.011 |
| Cognitive_factor    ~    superiortemporal_sa | 0.034 | 0.017 | 0.040 | 0.046 |
| Cognitive_factor    ~    fusiform_gmv | 0.051 | 0.016 | 0.001 | 0.066 |
| Cognitive_factor    ~    inferiorparietal_gmv | 0.002 | 0.014 | 0.886 | 0.003 |
| Cognitive_factor    ~    parahippocampal_gmv | 0.006 | 0.013 | 0.669 | 0.007 |
| Cognitive_factor    ~    paracentral_gmv | -0.013 | 0.013 | 0.321 | -0.016 |
| Cognitive_factor    ~    pericalcarine_gmv | -0.043 | 0.017 | 0.010 | -0.058 |
| Cognitive_factor    ~    precentral_gmv | 0.081 | 0.026 | 0.002 | 0.106 |
| Cognitive_factor    ~    precuneus_gmv | 0.006 | 0.014 | 0.685 | 0.008 |
| Cognitive_factor    ~    rostralmiddlefrontal_gmv | 0.008 | 0.014 | 0.581 | 0.010 |
| Cognitive_factor    ~    supramarginal_gmv | -0.006 | 0.015 | 0.670 | -0.008 |
| Cognitive_factor    ~    cingulatecingulum_fa | -0.069 | 0.012 | *p<0.001* | -0.089 |
| Cognitive_factor    ~    parahippocampalcingulum_fa | 0.021 | 0.012 | 0.086 | 0.028 |
| Cognitive_factor    ~    uncinate_fa | -0.077 | 0.017 | *p<0.001* | -0.101 |
| Cognitive_factor    ~    inferiorfrontooccipitalfasiculus_fa | 0.042 | 0.017 | 0.010 | 0.056 |
| Cognitive_factor    ~    temporalsuperiorlongitudinalfasiculus_fa | 0.105 | 0.017 | *p<0.001* | 0.138 |
| Cognitive_factor    ~    superiorcorticostriateparietalcortex_fa | 0.004 | 0.013 | 0.743 | 0.005 |
| Cognitive_factor    ~    fornix_exfimbria_fa | 0.003 | 0.011 | 0.764 | 0.004 |
| Cognitive_factor    ~    parahippocampalcingulum_md | 0.002 | 0.013 | 0.890 | 0.002 |
| Cognitive_factor    ~    inferiorlongitudinalfasiculus_md | -0.006 | 0.017 | 0.716 | -0.008 |
| Cognitive_factor    ~    forcepsmajor_md | 0.027 | 0.014 | 0.048 | 0.037 |
| Cognitive_factor    ~    temporalsuperiorlongitudinalfasiculus_md | -0.050 | 0.019 | 0.009 | -0.065 |
| Cognitive_factor    ~    striatalinferiorfrontalcortex_md | 0.024 | 0.016 | 0.153 | 0.029 |
| Cognitive_factor    ~    anteriorthalamicradiations_wmv | -0.040 | 0.017 | 0.017 | -0.055 |
| Cognitive_factor    ~    inferiorlongitudinalfasiculus_wmv | 0.046 | 0.016 | 0.005 | 0.062 |
| Cognitive_factor    ~    corpuscallosum_wmv | 0.020 | 0.023 | 0.391 | 0.028 |
| Cognitive_factor    ~    superiorlongitudinalfasiculus_wmv | 0.072 | 0.019 | *p<0.001* | 0.096 |
| Cognitive_factor    ~    superiorcorticostriateparietalcortex_wmv | 0.009 | 0.019 | 0.642 | 0.012 |
| Cognitive_factor    ~    inferiorfrontalsuperiorfrontalcortex_wmv | 0.011 | 0.019 | 0.548 | 0.015 |

*Supplementary Table 48. Standardized parameter estimates of how the grey & white matter metrics of each regularized region of interest together predict the cognitive factor*

| Path | CT | SA | GMV | FA | MD | WMV |
| --- | --- | --- | --- | --- | --- | --- |
| Cognitive_factor    ~    bankssts | **-** | -0.010 | **-** |  |  |  |
| Cognitive_factor    ~    caudalmiddlefrontal | 0.004 | **-** | **-** |  |  |  |
| Cognitive_factor    ~    cuneus | **-** | -0.008 | **-** |  |  |  |
| Cognitive_factor    ~    entorhinal | **-** | 0.010 | **-** |  |  |  |
| Cognitive_factor    ~    fusiform | 0.015 | **-** | 0.066 |  |  |  |
| Cognitive_factor    ~    inferiorparietal | **-** | **-** | 0.003 |  |  |  |
| Cognitive_factor    ~    insula | 0.001 | **-** | **-** |  |  |  |
| Cognitive_factor    ~    lateraloccipital | **-** | 0.034 | **-** |  |  |  |
| Cognitive_factor    ~    lateralorbitofrontal | -0.011 | **-** | **-** |  |  |  |
| Cognitive_factor    ~    medialorbitofrontal | -0.044 | **-** | **-** |  |  |  |
| Cognitive_factor    ~    middletemporal | 0.083 | **-** | **-** |  |  |  |
| Cognitive_factor    ~    paracentral | 0.054 | **-** | -0.016 |  |  |  |
| Cognitive_factor    ~    parahippocampal | 0.127 | **-** | 0.007 |  |  |  |
| Cognitive_factor    ~    parsorbitalis | 0.021 | 0.053 | **-** |  |  |  |
| Cognitive_factor    ~    pericalcarine | 0.034 | **-** | -0.058 |  |  |  |
| Cognitive_factor    ~    postcentral | **-** | -0.039 | **-** |  |  |  |
| Cognitive_factor    ~    precentral | **-** | 0.011 | 0.106 |  |  |  |
| Cognitive_factor    ~    precuneus | -0.072 | **-** | 0.008 |  |  |  |
| Cognitive_factor    ~    rostralanteriorcingulate | -0.023 | **-** | **-** |  |  |  |
| Cognitive_factor    ~    rostralmiddlefrontal | -0.021 | **-** | 0.010 |  |  |  |
| Cognitive_factor    ~    superiorfrontal | -0.026 | **-** | **-** |  |  |  |
| Cognitive_factor    ~    superiortemporal | -0.026 | 0.046 | **-** |  |  |  |
| Cognitive_factor    ~    supramarginal | **-** | **-** | -0.008 |  |  |  |
| Cognitive_factor    ~    anteriorthalamicradiations |  |  |  | **-** | **-** | -0.055 |
| Cognitive_factor    ~    cingulatecingulum |  |  |  | -0.089 | **-** | **-** |
| Cognitive_factor    ~    corpuscallosum |  |  |  | **-** | **-** | 0.028 |
| Cognitive_factor    ~    forcepsmajor |  |  |  | **-** | 0.037 | **-** |
| Cognitive_factor    ~    fornix_exfimbria |  |  |  | 0.004 | **-** | **-** |
| Cognitive_factor    ~    inferiorfrontalsuperiorfrontalcortex |  |  |  | - | **-** | 0.015 |
| Cognitive_factor    ~    inferiorfrontooccipitalfasiculus |  |  |  | 0.056 | **-** | **-** |
| Cognitive_factor    ~    inferiorlongitudinalfasiculus |  |  |  | **-** | -0.008 | 0.062 |
| Cognitive_factor    ~    parahippocampalcingulum |  |  |  | 0.028 | 0.002 | **-** |
| Cognitive_factor    ~    striatalinferiorfrontalcortex |  |  |  | **-** | 0.029 | **-** |
| Cognitive_factor    ~    superiorcorticostriateparietalcortex |  |  |  | 0.005 | **-** | 0.012 |
| Cognitive_factor    ~    superiorlongitudinalfasiculus |  |  |  | **-** | **-** | 0.096 |
| Cognitive_factor    ~    temporalsuperiorlongitudinalfasiculus |  |  |  | 0.138 | -0.065 | **-** |
| Cognitive_factor    ~    uncinate |  |  |  | -0.101 | **-** | **-** |

*Supplementary Table 49. Comparison of the model with free parameters and the model with parameters constrained to a same value for the grey & white matter model with the regularized regions. AIC/BIC are two information criterions. Pr(>Chisq): p-value of the chi-square ratio test.*

|  | Degree of freedom | AIC | BIC | Difference in degree of freedom | Pr (>Chisq) |
| --- | --- | --- | --- | --- | --- |
| Free model | 205 | 122955.0 | 123420.1 | NA | NA |
| Constrained model | 254 | 123470.8 | 123585.3 | 49 | *p<0.001* |

**Comparison between models with grey matter, white matter and grey & white matter metrics**

**Graph comparing the information criterions of the three models**

*Supplementary Figure 25. Aike information criteria of the three models: Grey Matter Model, White Matter Model and Grey & White Matter Model*

*Supplementary Figure 26. Bayesian information criteria of the three models: Grey Matter Model, White Matter Model and Grey & White Matter Model*

**Graph comparing the adjusted R-square of the three models**

*Supplementary Figure 27. Adjusted R-square of the three models: Grey Matter Model, White Matter Model and Grey & White Matter Model. The numbers on the bars represent the percentage of variance in cognitive performance explained by the models.*
